# Tight basis cycle representatives for persistent homology of large biological data sets

**DOI:** 10.1371/journal.pcbi.1010341

**Published:** 2023-05-30

**Authors:** Manu Aggarwal, Vipul Periwal

**Affiliations:** Laboratory of Biological Modeling/NIDDK, National Institutes of Health, Bethesda, Maryland, United States of America; McGill University Faculty of Science, CANADA

## Abstract

Persistent homology (PH) is a popular tool for topological data analysis that has found applications across diverse areas of research. It provides a rigorous method to compute robust topological features in discrete experimental observations that often contain various sources of uncertainties. Although powerful in theory, PH suffers from high computation cost that precludes its application to large data sets. Additionally, most analyses using PH are limited to computing the existence of nontrivial features. Precise localization of these features is not generally attempted because, by definition, localized representations are not unique and because of even higher computation cost. Such a precise location is a sine qua non for determining functional significance, especially in biological applications. Here, we provide a strategy and algorithms to compute tight representative boundaries around nontrivial robust features in large data sets. To showcase the efficiency of our algorithms and the precision of computed boundaries, we analyze the human genome and protein crystal structures. In the human genome, we found a surprising effect of the impairment of chromatin loop formation on loops through chromosome 13 and the sex chromosomes. We also found loops with long-range interactions between functionally related genes. In protein homologs with significantly different topology, we found voids attributable to ligand-interaction, mutation, and differences between species.

## Author summary

The relative arrangement of constituents in a biological system is often functionally significant. Persistent homology computes the existence of regions devoid of constituents that are surrounded by regions of high density, which we can think of as holes, that are robust to experimental uncertainties. An important question then is what purpose do these robust topological features serve in the underlying system? To investigate this, it is important to compute their precise locations. However, this computation suffers from high cost and non-uniqueness of representative boundaries of these holes. In this work, we developed a set of algorithms and a strategy that computes representative boundaries around holes with high precision in large data sets. We were able to process the human genome at a high resolution in a few minutes, a computation that extant algorithms could not attempt. We also determined locations of significant topological differences in crystal structures of protein homologous sequences. This work enables research into the functional significance of robust features in large biological data sets.

This is a *PLOS Computational Biology* Methods paper.

## Introduction

Quantitative observations of biological systems often lead to data sets that are discrete and have uncertainties [1]. The identification of robust features in such data sets is the impetus for the development of topological data analysis (TDA). Persistent homology (PH) is, perhaps, the most mathematically rigorous method developed for TDA. PH applies techniques developed in algebraic topology to find robust lacunae in discrete data sets. Most implementations of PH end their analysis at the stage where the existence of nontrivial homology cycles has been demonstrated. However, scientific interest in nontrivial cycles often requires finding specific locations for homology cycles. For example, the looping of chromatin strands of the genome is not random. It regulates gene expression by bringing regulators close to regulated genes that otherwise are far along the linear strands [2, 3]. Hence, determining the precise genomic location of loops is important. PH has found useful applications in areas as diverse as neuroscience [4], computational biology [5], natural language processing [6], the spread of contagions [7], cancer [8, 9], material science [10], computer graphics [11], among many others. However, its application is still limited because of the challenges of high computation cost and imprecise computations of locations of features.

**Fig 1:**
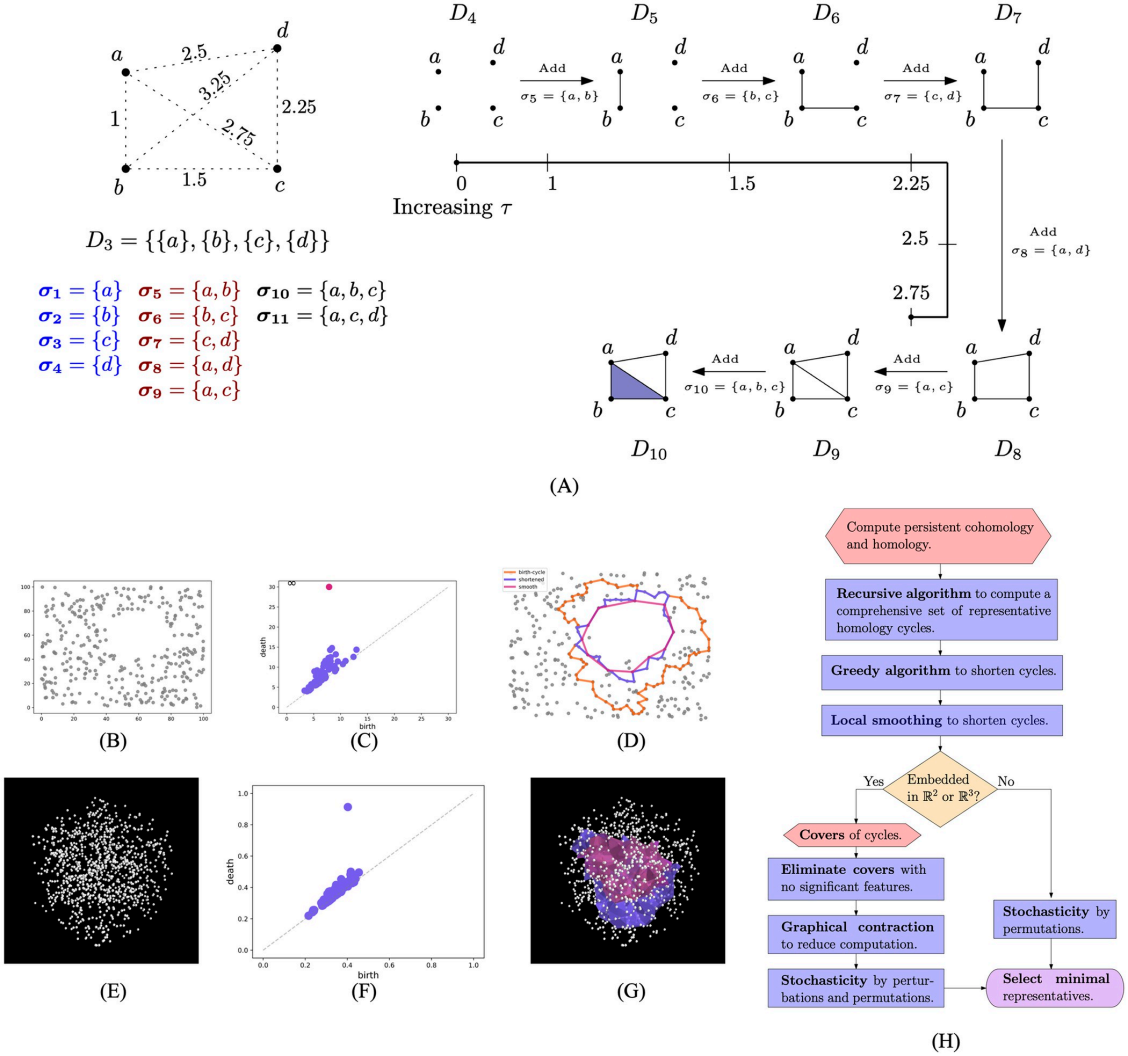
PH finds robust topological holes in discrete data sets. (A) A with 4 points and annotated pairwise distances. The simplicial complex (*D*_*i*_) at a spatial scale *τ* is defined as a set of simplices (*σ*_*i*_) with the diameter at most *τ*. At *τ* = 2.5, #holes in the simplicial complex increases from 0 to 1 when *σ*_8_ is added (*D*_8_). At *τ* = 2.75 #holes increase 1 to 2 when *σ*_9_ is added (*D*_9_). One hole gets filled in at *τ* = 2.75, when *σ*_10_ = {*a*, *b*, *c*} is added (*D*_10_), but one remains alive. (B) A noisy discrete data set in $R^{2}$ with one significant hole. (C) The births and death of holes are plotted as a persistence diagram. This example shows that only one hole has relatively high persistence. (D) Representative boundaries tighten as they are shortened by our algorithm. (E) A point-cloud in $R^{3}.$ No significant hole is visible. (F) PD shows that one feature has relatively high persistence. (G) Our algorithms improve geometric precision. The blue boundary is the birth-cycle from the recursive algorithm. The red boundary is the smooth cycle after applying the greedy shortening and smoothing algorithms. (H) Our strategy and our algorithms (highlighted) to compute a set of shortened homology representatives for large data sets.

Colloquially, we can think of the features that PH finds as ‘holes’ in the cloud of points that constitute a data set. Holes can also be interpreted as regions of lower density enclosed in regions of higher density. As one can surmise from the appearance of the term ‘density’, the features that PH finds are dependent on the scale of distances between neighboring data points. The use of an objective mathematically sound method, like PH, to compute the existence of holes is called for because the human eye is adept at pattern detection even when there is no pattern. For example, we can readily pick out a chain in a uniform distribution of constituents in a three-dimensional space [12]. On the other hand, we may be unable to find topologically significant holes in a 3D point-cloud by visual inspection (Fig 1E).

We briefly introduce terminology and background to explain the challenges in applying PH (see Table A in S1 Appendix for basic terminology). A *n*-simplex is a set of (*n* + 1) points (Fig A in S1 Text). A simplicial complex at a spatial scale of *τ* is defined as the set of all possible simplices with maximal pairwise distance at most *τ* between their points. Holes in a simplicial complex are computed as basis elements of homology groups of dimensions 1 and 2 of a simplicial complex, denoted by H_1_ and H_2_. They are also colloquially referred to as loops and voids (see Fig 1 for examples). If we construct simplicial complexes at different values of *τ*, increasing it from 0 to higher values, we get a sequence of simplicial complexes with potential changes in the homology bases of these complexes. A hole is said to be *born* at *τ* if a boundary is formed around it at that spatial scale, and it is said to *die* at *τ* if it gets *filled-in* (see Fig 1A). The *τ* values of the births and deaths of topological features or holes are recorded and plotted as a persistence diagram (PD). Every hole is classified by the range of values of *τ* across which it persists, called its *persistence* or *barcode* length and computed as death–birth. The key point is that the features with relatively high persistence are robust to larger experimental variability. PDs in Fig 1C and 1F show that examples in Fig 1B and 1E have one feature that is more robust to noise as compared to other features.

This multiscale construction and processing of high-dimensional simplices (0-, 1-, 2-, and 3-simplices to compute H_1_ and H_2_ PDs) incur a high computation cost. Test data sets used to benchmark PD computation are commonly limited to a few thousand points. Some of the different methods to compute PD are matrix reduction [13], Morse theory [14], and matroid theory [15]. Ripser is one of the most efficient software packages for computing PD [16] but it was unable to process human genome data sets because they contain millions of discrete points. In previous work, we developed Dory [17] and used it to compute the PD of human genome data sets within a few minutes using only 6 GB of memory. We also showed that its efficiency is not limited to these specific data sets since it used the least memory and run-time in almost all test data sets. A different approach to tackle the computational feasibility of PD is to approximate a topological structure with a large number of simplices by one with fewer simplices [18]. However, theorems justifying such a coarsening so as to not lose information about possibly functionally important features, are hard to come by. Moreover, adding sampling stochasticity to an approach that is trying to overcome data uncertainty is counter-intuitive.

Most implementations of PH are limited to computing PD and thereby demonstrating the existence and persistence of holes, but not specifying their locations. Therefore, they do not allow a scientific assessment of the functional significance of holes. Why is the location information elided? The location of a hole can be estimated by computing a representative boundary around it. However, by the very definition of simplicial homology, representative boundaries around topological features are not uniquely defined [19] so the computed boundary may be geometrically imprecise (see Fig 1D). Furthermore, computing candidate representative boundaries is expensive in both memory usage and run time. Hence, the development of efficient and scalable algorithms to compute PD and precise representative boundaries of persistent features is an active area of research.

One approach to computing a canonical set of representative boundaries has been to define minimal boundaries as solutions to an optimization problem. For example, a possible set of boundaries is one that minimizes the aggregated weight of boundaries at different spatial scales [20]. Solutions to such optimization problems are called *optimal homologous cycles*. Strategies for their efficient computation were introduced in [21]. However, computing optimal homologous cycles still is of order *O* (*n*^11^) for a data set with *n* points [22]. Hence, they are usually computed for small data sets. A comparison of different possible optimization functions and implementations is discussed in [23].

Our contribution in this work is a set of algorithms that can compute minimal representative boundaries around *significant* loops and voids in large data sets. Fig A in S1 Appendix shows the well-known matrix reduction algorithm to compute PH (see S1 Appendix for background and terminology). Fig 1H shows an outline of our strategy and highlights our algorithms to compute a minimal set of representatives. We classify a hole as significant if it has persistence larger than a threshold *ϵ* (a higher value means robust to larger variability in the data set) and is born at a spatial scale of at most *τ*_*u*_ (a lower value suggests holes with denser boundary). First, we developed a recursive algorithm that computes a comprehensive set of representative boundaries around all nontrivial holes (persistence > 0). Note that this is not always possible in extant algorithms because of high computation cost. We call these boundaries birth-cycles. Next, we developed a greedy algorithm that decreases the lengths of birth-cycles to give a set of shortened representative boundaries. Third, we implemented a local smoothing of the boundaries. Fig 1D and 1G show examples of shortened and smoothed H_1_ and H_2_ representatives. Note how they tighten around the respective holes when they are shortened. Fourth, the algorithm identifies sub-regions of a spatial embedding of the data set that contain significant features. Consequently, we use a divide-and-conquer strategy to compute PH of all significant features when it is not possible to do so for the full data set due to high computational cost. We also developed a graphical contraction that can decrease the number of sub-regions. Finally, we introduce stochasticity in two different ways, only using the implicit inherent random ordering of the standard PH algorithm without any subsampling, that help to overcome the greedy algorithm’s local minimum problem.

We applied our algorithms to two different biological data sets. First, we computed loops in various Hi-C data sets and analyzed their possible biological significance. We compared H_1_ loops with HiCCUPS, a commonly used algorithm to estimate chromatin loops [24]. Second, we computed voids in protein crystal structures and showed precise structural differences between protein homologs that have significantly different topology. Most analyses of protein 3D structures based on PH, use information from the PDs [25, 26] and do not consider representative boundaries. An analysis of protein structures that goes beyond PDs is given in [27]—operations on representative boundaries of topological features are used to explain changes in structural orientations of a specific protein. However, tight representatives are not considered in their work, which studies structures of proteins that have asymmetries and multiple complex sub-units.

## Results

### Genome-wide high resolution loops in human genome computed within a minute

We analyzed Hi-C data sets for control and auxin-treated human cells from [28], which showed that the addition of auxin led to a loss of chromatin loops. We observed a congruent trend for H_1_ loops at 1 kb resolution. Spatial distances between genomic bins were estimated as the multiplicative inverse of Hi-C interaction frequencies. We denote the resulting spatial correlation matrices by ${\hat{D}}^{c}$ and ${\hat{D}}^{a}$ for control and auxin-treated data sets, respectively. S1(A) Fig shows distributions of the estimates, ${\hat{d}}_{ij}^{c}$ and ${\hat{d}}_{ij}^{a},$ for bin distances |*i* − *j*| = 1, 2, 3, and 4. S1(B) Fig shows that their means and medians increase with bin distances. This is a sanity check for our estimates since we expect that in most cases the bins that are farther apart along the linear chromosome will also be farther in the folded chromosome. We defined a feature to be significant if it is born at a spatial scale less than or equal to *τ*_*u*_ = 100 and has persistence at least *ϵ* = 50. These choices are based on the fact that 100 is above the 75%-ile of estimates for bin distance of 1 and 150 is below the 25%-ile of estimates for bin distance of 2 (dashed lines in S1 Fig). Computing PH up to *τ* = *τ*_*u*_ + *ϵ* = 150 resulted in 3,206,797 and 2,701,615 valid unique edges in ${\hat{D}}^{c}$ and ${\hat{D}}^{a},$ respectively. Since there are 3088281 bins at 1 kb resolution, the total number of possible edges is $\left(\begin{array}{c} 3088281\\ 2 \end{array}\right)\approx 4.7\times 10^{12}$. Our algorithms computed PD and representative boundaries in around one minute using 3.1 GB of memory for ${\hat{D}}^{c}$ and 35 seconds with 3 GB for ${\hat{D}}^{a}$ (see Table 1).

**Table 1:** Our algorithms process large data sets within a minute. *n* is the number of points, *τ* = *τ*_*u*_ + *ϵ* is the threshold for PH computation, #H_1_ is the number of nontrivial H_1_ features, #cycles is the number of cycles born at a spatial scale less than or equal to *τ*_*u*_, T_tot_ is the time taken in seconds to compute the PD, birth-cycles, and shorten and smooth birth-cycles, while M_tot_ is the peak memory usage in GB.

	*n*	*τ*_*u*_	*ϵ*	*n*_*e*_	#H_1_	#cycles	T_tot_ (s)	M_tot_ (GB)
Control	3088281	100	50	3206797	375281	43855	67	3.1
Auxin-treated	3088281	100	50	2701615	148628	21363	35	3

#### Our algorithm significantly shortens representative boundaries around topologically significant features

S2(A) Fig shows that the median and 75%-ile boundary lengths decreased across multiple log scales (base 2) after being shortened by the greedy algorithm. S2(B) Fig shows that smoothing of the shortened cycles reduced some to degenerate cycles of length 2 (lowermost humps in the distribution of the smooth cycles lacking in the distribution of lengths of shortened cycles). Specifically, 902 cycles in control and 209 in auxin-treated were reduced to degenerate cycles. These were ignored in subsequent analysis.

#### Treatment with auxin results in loss of H_1_ loops

**Fig 2:**
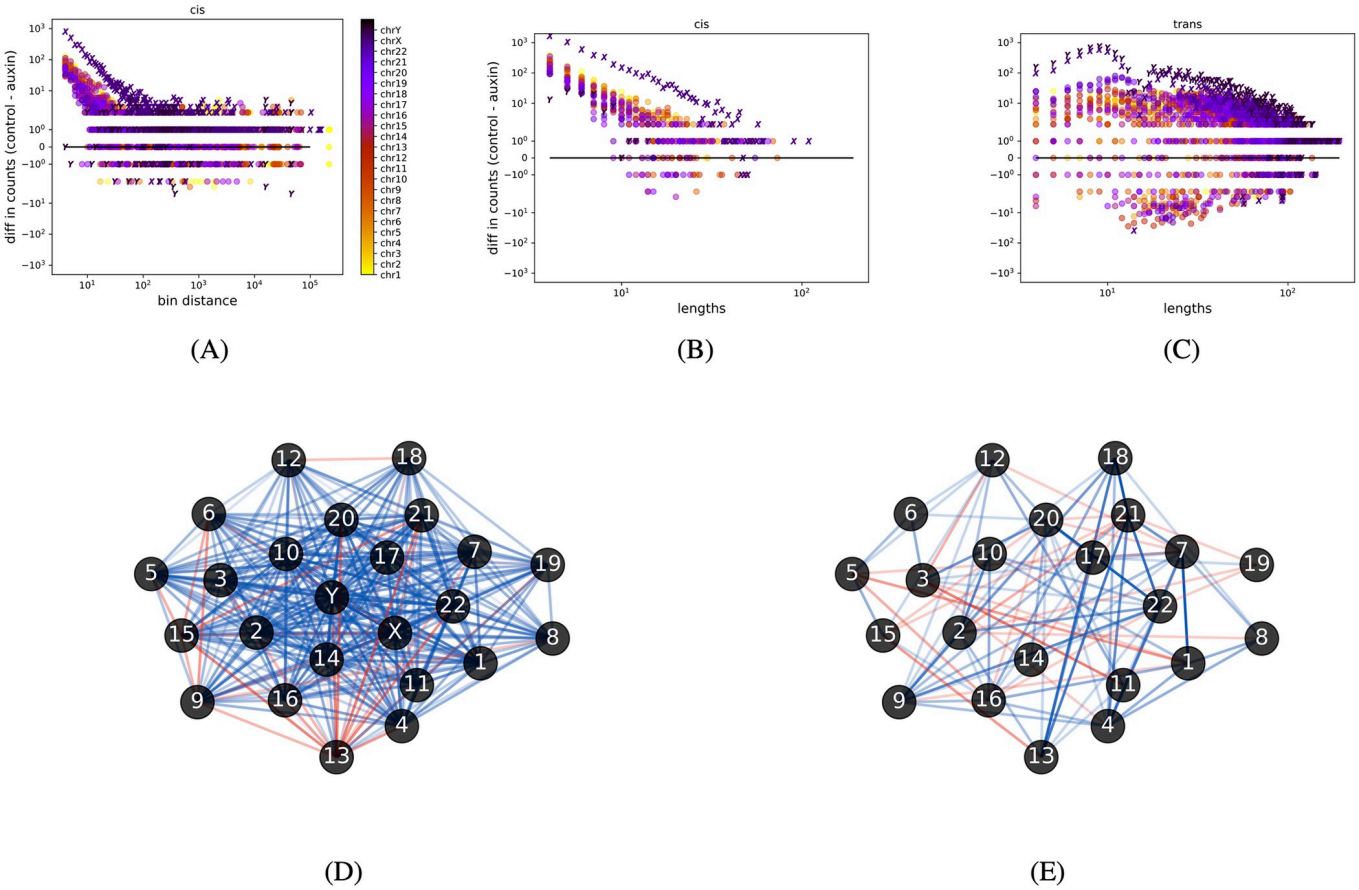
Both cis- and trans-cycles decrease in number upon treatment with auxin. (A) and (B) The number of cis-cycles decreased upon the addition of auxin, irrespective of cycle length and the maximal bin distance between contiguous bins. (C) The number of trans-cycles also decreased. (D) The opacity of edge (*c*_*i*_, *c*_*j*_) indicates a difference in the number of trans cycles between control and auxin-treated that go through chromosomes *c*_*i*_ and *c*_*j*_. Blue indicates that there are more trans cycles in control and red signifies that there are more in auxin-treated. Chromosome 13 stands out with many red edges. (E) Same as previous but without the sex chromosomes. There are now very few red edges at chromosome 13, indicating that auxin treatment leads to a larger number of trans-cycles that go through chromosome 13 and the sex chromosomes.

We computed 42695 H_1_ loops in control and 21137 in auxin-treated that are potentially topologically significant. Hence, auxin treatment resulted in a loss of around 50% of possibly significant H_1_ loops. For further analysis, we considered cis- and trans-cycles separately. If all bins in a H_1_ loop are on the same chromosome, we call it a cis-chromosome cycle or a cis-cycle. Otherwise, we call it a trans-chromosome cycle or a trans-cycle. Analysis of the computed cycles showed that the control data set has a higher number of cis-cycles in all chromosomes, irrespective of their length and maximal bin distance between contiguous bins in the cycle (see Fig 2A and 2B). We also note that chromosome X has a significantly higher number of cis- and trans-cycles as compared to autosomal chromosomes. This topological difference is an interesting coincidence because it is known that the sex chromosomes and autosomal chromosomes have various differences in their composition and function [29]. The differences in the number of cycles at bin-distances of up to 10^5^ in Fig 2A indicates that we found cis-cycles with contiguous bins that are 10^5^ bins apart along the linear chromosome. This provides evidence for long-range interactions. The number of trans-cycles also decreased upon treatment with auxin (Fig 2C). For every pair of chromosomes, we counted the number of trans-cycles that go through the pair. We plot the difference in the counts between the control and auxin treatment as a graph in Fig 2D. A red edge between a pair of chromosomes (*c*_*i*_, *c*_*j*_) implies that more trans-cycles are going through *c*_*i*_ and *c*_*j*_ in auxin-treated than in control, and blue edges indicate the opposite. The predominance of blue edges indicates that for almost all chromosome pairs there are more trans-cycles in control as compared to auxin-treated. An exception that stands out is chromosome 13 which has more red edges. We repeated this analysis without the sex chromosomes to analyze trans-cycles that go through only the autosomes. Fig 2E shows that such trans-cycles through chromosome 13 are mostly blue. Hence, the auxin-treated data set has more trans-cycles that go through chromosome 13 and the sex chromosomes. This might yield insights into functional relations between chromosome 13 and the sex chromosomes. For example, ambiguous genitalia is known to be associated with sex chromosome disorders. At least one case has been observed of ambiguous genitalia with the autosomal chromosome anomaly of ring chromosome 13 [30].

#### Functionally related genes interact along long-range H_1_ loops

**Fig 3:**
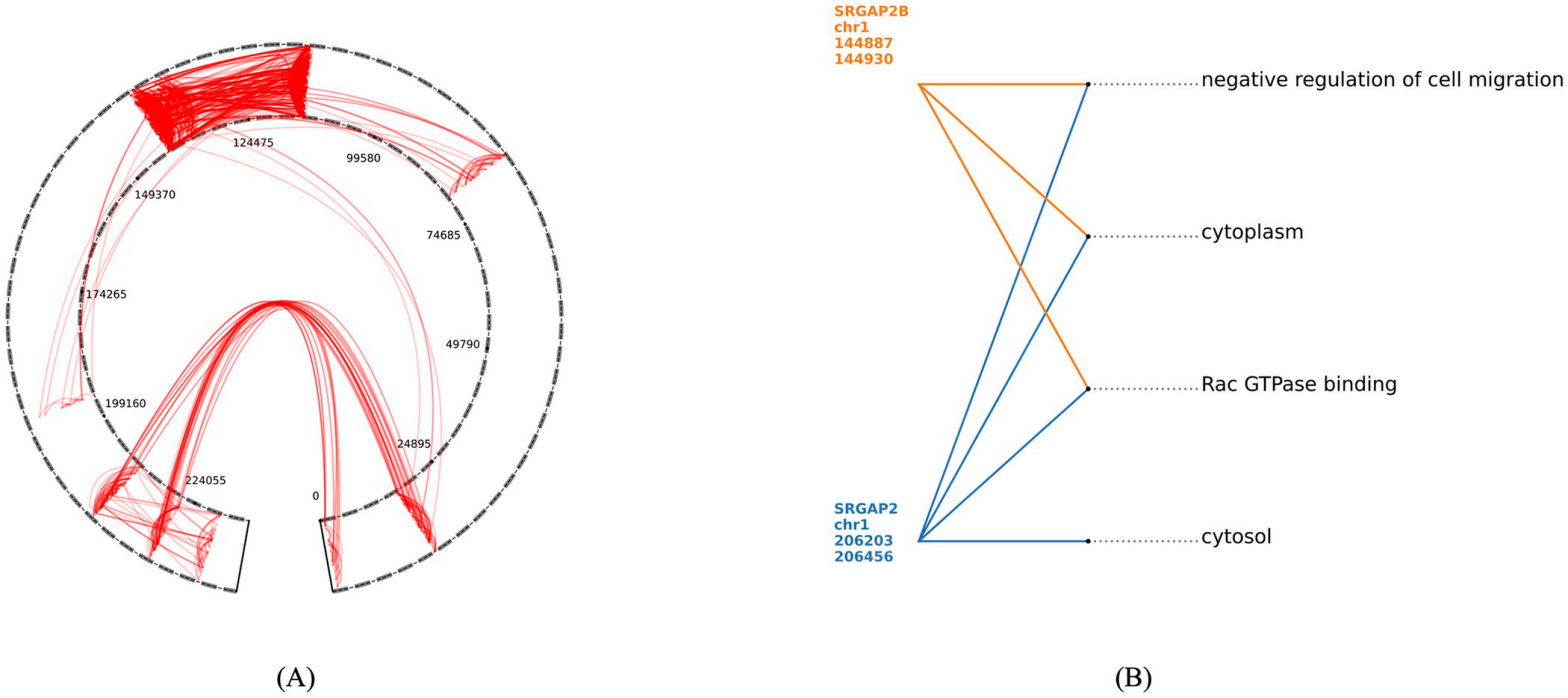
Long-range H_1_ loops in chr1. (A) All long-range interaction loops. (B) An example of functionally related genes on one of the long-range H_1_ loops. The numbers under each bin are the 1 kb genomic bins of chr1 which contain the gene.

Fig 3A shows H_1_ loops computed at 1 kb in chromosome 1 (chr1) with at least one pair of adjacent bins (*b*_*i*_, *b*_*j*_) such that the bins are very far along the linear chromosome, specifically, |*b*_*i*_ − *b*_*j*_| ≥ 10000 kb. Loops with such long-range interactions between bins that are very far along the linear chromosome might have functional significance. There were 189 such chromatin loops in chr1. Fig 3B shows an example of functionally related genes along one of the long-range H_1_ loops.

### Consistency and biological validation of the computed tight representatives in the human genome

Hi-C experiments were conducted by [31] under different protocols on fully differentiated human foreskin fibroblast (HFFc6) cells. The protocols differ in cross-linking agents—1% formaldehyde (FA) or 1% formaldehyde followed by incubation with 3mM disuccinimidyl glutarate (FA + DSG), and in combinations of nucleases for chromatin fragmentation (restriction enzyme)—DpnII, DdeI, and MNase. Table 2 shows the different protocols.

**Table 2:** Different Hi-C experiment protocols in this case study.

Exp. index	Cross-linking agent	Restriction enzyme
1	FA	DpnII
2	FA+DSG	DdeI
3	FA+DSG	DpnII+DdeI
4	FA+DSG	DpnII
5	FA+DSG	MNase

#### The majority of shortened H_1_ loops are computed consistently for experiments with similar protocols

H_1_ loops were computed for each experiment at three different sets of parameters with increasing birth-thresholds (see S1 Table). Experiments 2, 3, and 4 have the most similar protocols because they use the same combination of cross-linking agents and similarly sized restriction enzymes. For each of these experiments, at least 50% of computed shortened H_1_ loops had a loop within a bin-distance of 1 in the other two experiments (see S4(B), S4(C) and S4(D) Fig). This percentage was lower when compared to H_1_ loops in experiment 1, that does not use the extra cross-linking agent (see S4(A) Fig). Moreover, a larger percentage of shortened H_1_ loops computed for experiment 5 did not have a nearby loop in other experiments (see S4(E) Fig). This is because a larger number of loops are computed for this experiment which uses a shorter restriction enzyme. S4 Fig shows consistency for computation at the parameter set with the largest birth-thresholds. Results are similar at lower thresholds (see S5 Fig).

#### PH finds loops that are not found by HiCCUPS

**Fig 4:**
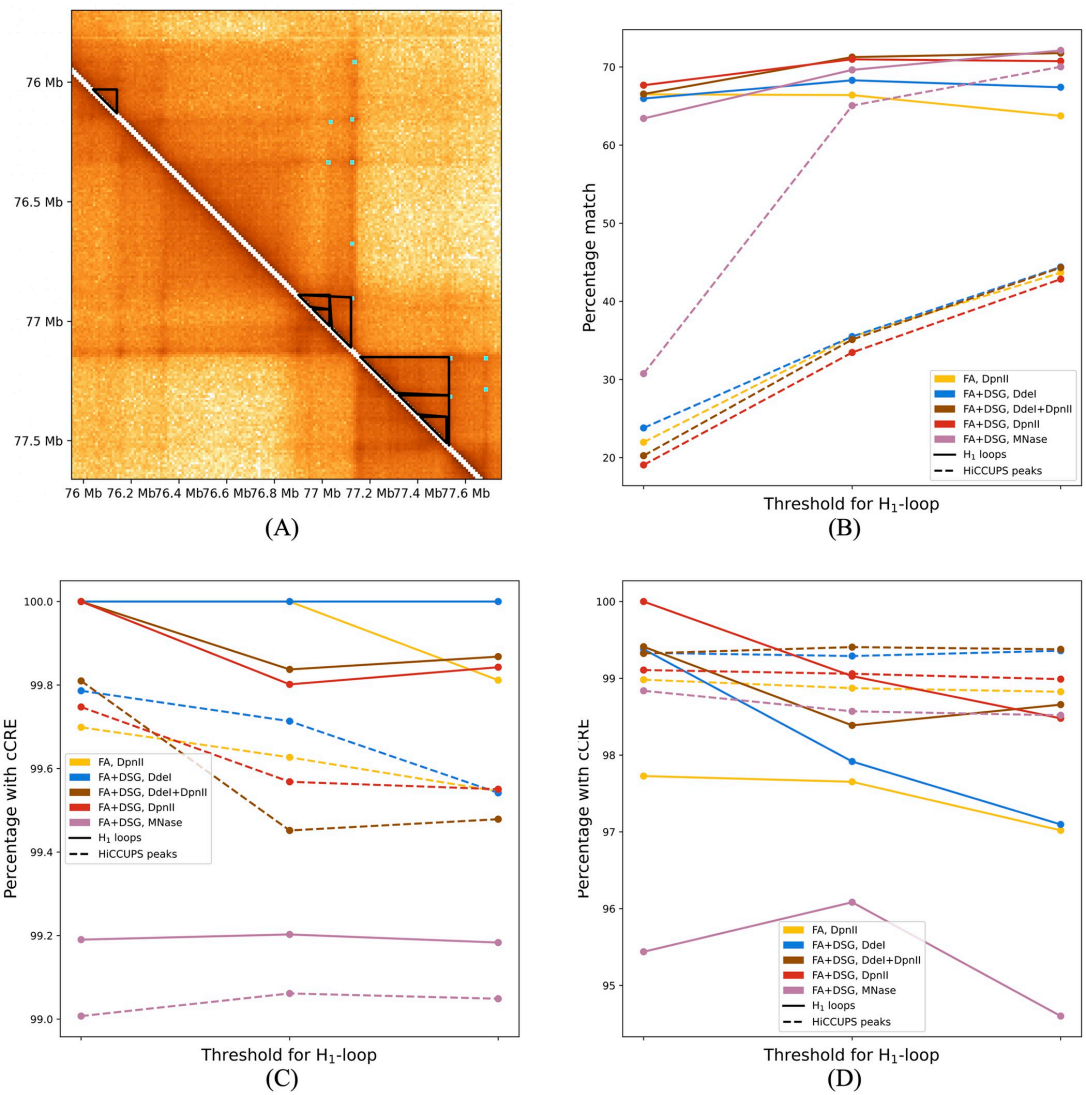
HiCCUPS peaks and H_1_ loops at three different birth-thresholds for chr1 at 10 kb resolution. (A) A subregion of chr1 showing H_1_ loops (black lines) and HiCCUPS peaks (teal dots) overlaid on heatmap of Hi-C contact matrix for experiment 1. Darker regions indicate higher balanced interaction frequency. (B) Percentages of H_1_ loops that match a HiCCUPS peak (solid lines) and of HiCCUPS peaks that match H_1_ loops (dashed lines). Around 30% of H_1_ loops are not found by HiCCUPS peaks. (C) and (D) Percentage of H_1_ loops and HiCCUPS peaks that have cCRE for matched (C) and unmatched (D). In all cases, the percentage of loops and peaks that have cCRE is high (> 95%), including H_1_ loops not found by HiCCUPS.

HiCCUPS reports pairs of genomic bins (*b*_*i*_, *b*_*j*_), called HiCCUPS peaks, representative of chromatin loops anchored at bins *b*_*i*_ and *b*_*j*_. Fig 4A shows a subregion of chr1 with H_1_ loops and HiCCUPS peaks overlaid on the heatmap of Hi-C interaction matrix of experiment 1 at 10 kb resolution. For all experiments and at all different birth-thresholds, around 60% to 70% of H_1_ loops match HiCCUPS peaks (see Fig 4B). Hence, around 30% of H_1_ loops are not computed by HiCCUPS. The percentage of HiCCUPS peaks that match H_1_ loops increases from 20% to 40% as the birth-threshold is increased. This is because there are HiCCUPS peaks with low balanced Hi-C interaction counts (see S6 Fig). Since spatial estimates are the multiplicative inverse of the balanced counts, H_1_ loops matching such HiCCUPS peaks can possibly be found only when birth-threshold is high.

#### Loops exclusive to PH contain cCREs and are enriched in histone markers

**Fig 5:**
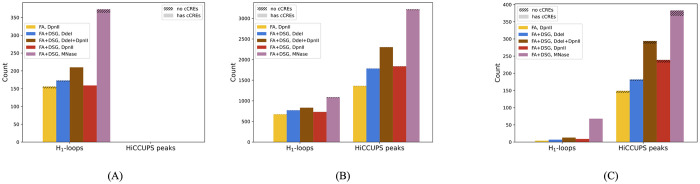
cCREs in all H_1_ loops (computed at largest of the three thresholds) and in HiCCUPS peaks classified by their maximum range of interaction. (A) Short-range (< 100 kb). PH finds many short-range loops, almost all of which contain cCREs. HiCCUPS does not find any short-range loops. (B) Mid-range (100 kb up to 1 Mb). Almost all of the mid-range H_1_ loops and HiCCUPS peaks contain cCREs. PH finds fewer mid-range loops. (C) Long-range (1 Mb and greater) All H_1_ long-range loops contain cCREs. There are a few HiCCUPS long-range peaks that do not contain cCREs.

**Fig 6:**
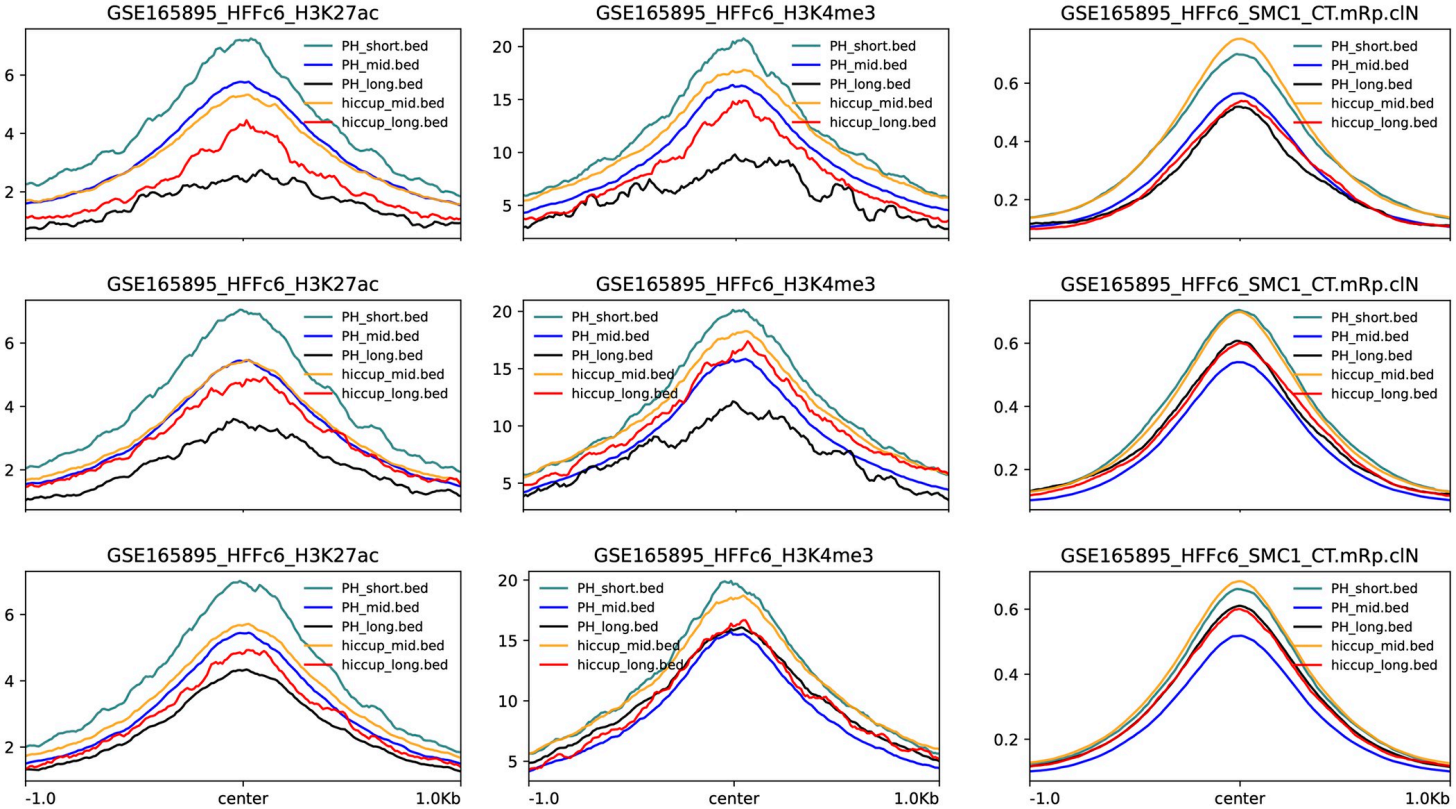
Mean biomarker enrichments in H_1_ loops (computed at largest of the three thresholds) and HiCCUPS peaks. Columns, left to right–H3K27ac, H3K4me3, and SMC1. Rows, top to bottom–experiments 1, 3, and 5. Legend–PH_short, PH_mid, and PH_long stand for short, mid, and long-range H_1_ loops. Short-range H_1_ loops show the highest enrichment in H3K27ac and H3K4me3 in all experiments.

Fig 4D shows that a high percentage (> 95%) of H_1_ loops that are not computed by HiCCUPS have cCREs. Further, loops and peaks were classified into short-range, mid-range, and long-range based on whether they facilitate interaction between bins that are less than 100 kb, at least 100 kb and less than 1 Mb, and greater than 1 Mb apart along the linear chromosome, respectively [32]. Fig 5A shows that HiCCUPS does not compute any short-range loops and almost all of these have cCREs. Additionally, genomic bins in these loops, but not in mid-range and long-range loops, have the highest enrichment in H3K27ac and H3K4me3 histone markers (see Fig 6). All of the long-range H_1_ loops have cCREs, whereas there are a few long-range HiCCUPS peaks that do not have cCREs (see Fig 5C).

#### H_1_ loops with multiple mid-range interactions have higher enrichment in SMC1

**Fig 7:**
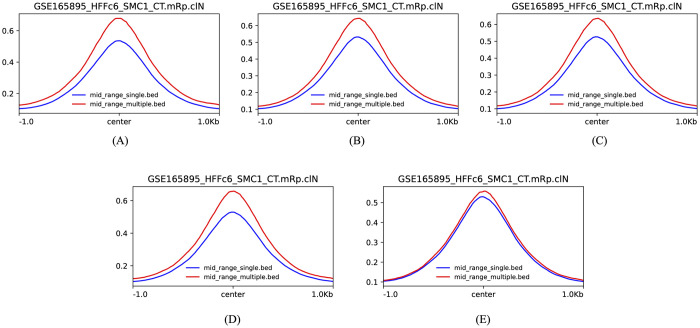
H_1_ loops with multiple mid-range interactions have higher enrichment in SMC1 as compared to those with single mid-range interactions in experiments 1 to 5.

H_1_ loops provide information about all bins in the loop. This enabled us to classify loops based on whether they had single or multiple mid-range interactions. Fig 7 shows that loops with multiple mid-range interactions have higher enrichment in SMC1.

#### Almost all H_1_ loops that are common to all experiments have cCREs

There were 106 short-range, 205 mid-range, and 0 long-range H_1_ loops common to all experiments. There was only one mid-range loop that did not contain cCCREs.

#### PH finds biologically relevant loops at higher resolutions

S7 Fig shows numbers of short-, mid-, and long-ranged H_1_ loops computed at different resolutions of 1, 5, and 10 kb in chr1 for experiment 5. The majority of loops in all but one category contain cCREs. Only short-range loops computed at 1 resolution have a majority without cCREs. Experiment 5 was picked for multiple resolution analysis because distributions of spatial estimates at lower bin-distances are more distinct as compared to other experiments (see S8 Fig).

### Protein homologs with significantly different topology

#### There are protein homologs with significantly different H_2_ topology

We computed PH for 174, 574 publicly available Protein Data Bank (PDB) entries with at least 10 and at most 20, 000 atoms in their backbone (N, C, C*α*, and O atoms). Dory [17] computed PH up to and including H_2_ efficiently, taking at most a few minutes for proteins with the most number of atoms in their backbone (S9(A) Fig). 12, 198 sequences had at least one significant H_2_ feature. Protein homologs were defined based on sequence similarity since it is generally presumed that sequences with high similarity did not arise independently and share a common ancestor [33]. We found 874 unique sets of homologous protein sequences such that each set has at least one sequence with a significant H_2_ feature. Pairwise comparisons of PDs found 25 sets of homologous sequences with significant differences in H_2_ topology within the set. PDs were compared by computing a distance between them that we call the L_0_ distance. S10 Fig shows that this metric agreed with the commonly used metric of Bottleneck distance to compare PDs, while being computationally faster by many orders of magnitude. S12 Fig shows all pairs of homologous sequences with significantly different topology.

#### Tight H_2_ representatives around distinct singular voids were computed within a few minutes

There were cumulatively 110 PDB entries in the 25 sets of homologous sequences from before. 71 of these had at least one significant void. The largest of these proteins had around 4000 atoms in its backbone. Our strategy reduced this to computing PH and H_2_ representative boundaries for smaller data sets, the largest having 800 points, and took from a few seconds to a few minutes to process each of these data sets (S9(B) Fig). Computing H_2_ boundaries makes it possible to plot the location of voids. It is visually evident from S13 Fig that all computed shortened representative boundaries for significant H_2_ features are around distinct singular voids.

#### Ligand-binding can lead to significant changes in H_2_ topology

**Fig 8:**
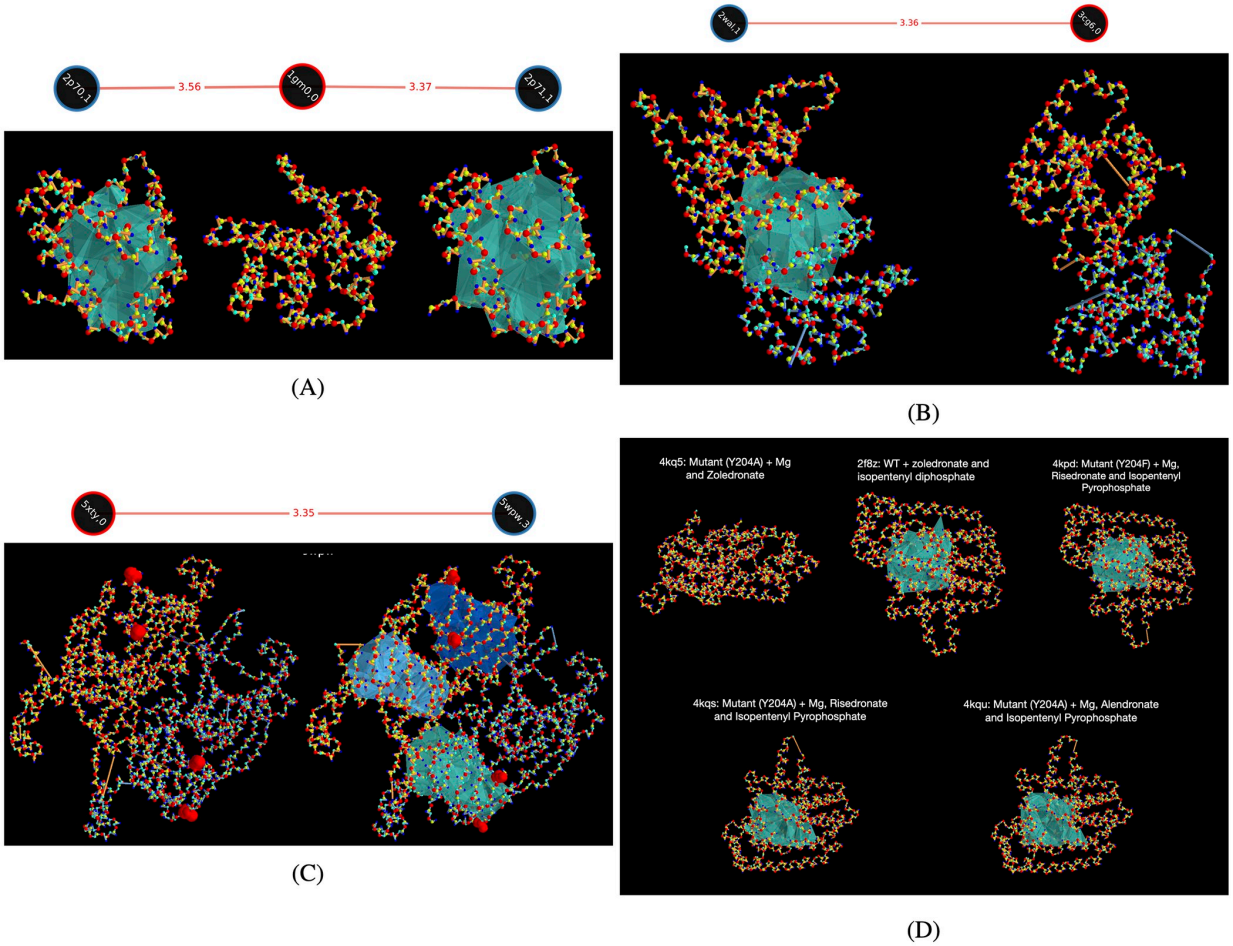
Examples of computed shortened H_2_ representatives in protein crystal structures. The numbers on the edges of the graphs show the L_0_ metric that we defined to quantify distance between two PDs. (A) 1gm0 is a form of pheromone-binding protein from *Bombyx mori* that has no significant voids. Its bound configurations—with bell pepper odorant (2p70) and with iodohexadecane (2p71)—have significant voids at the binding sites. (B) Dimeric configuration of GADD45g in humans has a void, but not in mice. (C) Two mutations (large red markers) in each of the two chains of protein Cocosin lead to the formation of three significant voids. (D) Homolog set corresponding to human Farnesyl Pyrophosphate Synthase (FPPS), an enzyme of the mevalonate pathway. The computed representatives are almost similar across the different PDB entries with similar structures, indicating consistency in the results of our optimization strategy to compute minimal representatives.

Pheromone-binding protein of *Bombyx mori* has no significant voids in its unbound form, but has voids in its bound states with bell pepper odorant (PBD 2p70) and iodohexadecane (PBD 2p71) (see Fig 8A). The binding-site is inside the void.

#### Homologs found in different species with different topology

The growth arrest and DNA damage (GADD45) genes are a highly conserved family of proteins (GADD45a, GADD45b, and GADD45g) that respond to stress in mammalian cells and have a crucial role in DNA repair. A dimeric conformation of GADD45g from humans (PBD 2wal) has a significant void, but the dimeric configuration reported for mice (PBD 3cg6) does not have any voids (see Fig 8B). This structural difference might lead to differences in gene expression. For example, a closely related gene in the same family, GADD45a, is up-regulated in humans but down-regulated in mice upon irradiation [34].

#### Mutations can significantly change topology

Cocosin, a protein in coconut fruit, is a possible food allergen. Its reported crystal structure (PDB 5xty) has no significant voids, but mutations in two residues (PDB 5wpw) result in formation of 3 significant voids (see Fig 8C).

#### Computed shortened H_2_ representative boundaries are consistent across sequences with similar structures

Even though our algorithm cannot theoretically guarantee canonical minimal representatives, it consistently locates voids with high precision. As empirical evidence, similar representative boundaries were computed for significant voids in wild-type and mutants of human Farnesyl Pyrophosphate Synthase (see Fig 8D). This indicates that the sole significant void found in these homolog sequences is at the same location with respect to the protein backbone.

## Discussion

PH is an immensely popular method for topological data analysis because it is based on clear mathematical foundations and it is computable with, essentially, basic linear algebra. The existence of nontrivial robust holes in noisy experimental observations, as computed by PH, has found applications across diverse areas of research. However, computing the locations of holes has not received much attention because of the non-uniqueness of their boundaries and the high computational cost. This information, in scientific contexts, is crucial for understanding the functional significance of nontrivial features. In this work, we provided a set of algorithms and a strategy to compute locations of nontrivial holes with high precision. Moreover, we were able to process large data sets with millions of points. We demonstrated this in two disparate areas of biology.

Our method is based on the established matrix reduction algorithm that reduces boundary or coboundary matrices to compute PH. The following is a summary of our contributions and the main ideas of our strategy: We use duality between cohomology and homology to reduce the computation cost of reducing the boundary matrix. We show that the reduction operations of the boundary matrix give a comprehensive set of representative boundaries in every scenario that we call birth-cycles. We developed a recursive algorithm that computes reduction operations for the boundary of any given simplex on the fly. This eliminated memory overhead since the entire matrix of reduction operations is never stored in memory. It was additionally optimized in both memory usage and run-time by selective storage of reduction operations of some simplices. It may be interesting to explore whether these simplices are related to critical simplices in Morse theory. The next major algorithm is the greedy shortening algorithm that updates the set of representative boundaries with shorter ones. This algorithm is optimized by division into different cases. Greedy shortening decreased the lengths of representatives significantly in our case studies, shortening them by multiple log-scales in some data sets. We also locally smooth these boundaries. If an embedding of the data set is available, we use the shortened and smoothed boundaries to find subsets of the complete data set that contain significant features. In our case studies, the number of points in these subsets was much less than the number of points in the full data set. This reduced the computational cost of subsequent analysis. We add stochasticity in two different ways to possibly find shorter boundaries in the embedding of these subsets. From the multiple computed sets of boundaries, we construct a set of minimal representatives. It is important to note here that our introductions of stochasticity were analytical tools, in the same way that randomization can improve the performance of sorting algorithms, and in no way was the data reduced or subsampled.

We suggest some alternative algorithm design choices that could be explored and may lead to improvements in our methodology. For example, we have defined the cover of an embedded set of points as the smallest hyper-rectangle that contains the points on it or inside it. A tighter cover of the points in the embedding might be to fit an oriented bounding box by computing eigenvectors of the covariance matrix of embedded points. However, it will incur a higher computational cost in comparison to the current choice of cover. As another example, the perturbation parameter of a cover is based on the number of significant features being the same across all of its perturbations. This is a weak check to ensure that the topology across perturbations does not change significantly. A stronger check could be to use the L_0_ distance between two PDs.

We list some technical limitations and possible resolutions as avenues for future work. First, the definition of covers depends on the availability of a spatial embedding. It is possible to define covers using only the pairwise distances. A comparison of computational benchmarks and results for different definitions of covers may be useful. Second, we assume that it is feasible to compute PH up to the maximum possible threshold for covers of shortened and smoothed boundaries. If that is not feasible, then we cannot compute representative homology boundaries for the cover(s). Instead, we can compute birth-cycles by computing PH up to and including the threshold of *τ* = *τ*_*u*_ + *ϵ* for all permutations of all perturbations. A strategy would have to be developed to construct a set of minimal representatives from the multiple sets of shortened and smoothed birth-cycles. Fourth, Dory can compute H_1_ and H_2_ only for the Vietoris-Rips filtration and for coefficients in $Z_{2}$.

To illustrate its applications in biology, we provided two case studies. First, we computed loops in the embedding of the human genome in the nucleus at the high resolution of 1 kb. We found that auxin affects trans-cycles that go through chromosome 13 and the sex chromosomes differently, as compared to all other chromosomes. We also found H_1_ loops with long-range interactions between functionally related genes. In the analysis of Hi-C data sets of five different protocols, we showed that loops found by PH and not by HiCCUPS, contain cCREs and are enriched in histone markers. Specifically, PH found many short-range loops, none of which were found by HiCCUPS, that showed the highest enrichment in H3K27ac and H3K4me3 across all different categories of H_1_ loops and HiCCUPS peaks. This comparison was conducted at 10 kb resolution because all unique HiCCUPS peaks computed at this resolution are reported as valid loops [31]. In contrast to HiCCUPS which only reports anchors of chromatin loops, PH gives information about all bins in a loop. This allowed us to determine H_1_ loops with multiple mid-range interactions. We found that such loops have higher enrichment in SMC1 as compared to loops with only single mid-range interactions. We computed H_1_ loops at different resolutions for the experiment with the shortest restriction enzyme and showed that even at higher resolutions of 1 and 5 kb, majority of mid-range and long-range H_1_ loops contain cCREs. Only short-range H_1_ loops at the highest 1 kb resolution had a majority without cCREs, which might be attributed to noisier Hi-C counts at higher resolution. We note that loops computed by PH depend upon the model used to estimate the spatial pairwise distance between genomic bins from raw Hi-C counts. Analogously, HiCCUPS peaks depend on the choice of the kernel to compute peak enrichment and other hyperparameters like max_loci_separation and clustering_radius. Second, we computed voids in protein homologs with a different number of significant H_2_ features. We highlight three examples that showed the difference in significant voids related to ligand interaction, mutation, and difference in species. We note that the existence of these topological differences between homologs may not lead to functional differences. Causal relationships between structural differences and function require experimental validation. A fourth highlighted example shows consistency and precision in the computed representatives across multiple protein structures in a homolog set. In conclusion, we believe that this work enables research into the functional significance of robust features in a plethora of large biological data sets.

## Methods

### Computing homology and representative boundaries

Two user-defined thresholds are defined, lower-limit on persistence and upper-limit on the birth of cycles, denoted by *ϵ* and *τ*_*u*_, respectively. We call *τ*_*u*_ as birth-threshold and *τ* = *τ*_*u*_ + *ϵ* as PH-threshold. A higher value of *ϵ* will restrict computation to more robust features. A lower value of *τ*_*u*_ will compute denser boundaries. Persistence is computed up to the spatial scale of *τ*. PH is computed using Dory that reduces the coboundary matrix efficiently for large data sets [17]. There exists a bijection between pivots of reduced coboundary matrix and of reduced boundary matrix [35]. Hence, the reduced coboundary matrix informs exactly which columns of the boundary need to be reduced (see Section 1 in S1 Text for details). The columns of the reduced boundary matrix form a set of representative boundaries. However, if there are features that do not die, then this will not be a comprehensive set of boundaries. Hence, we consider the set of reduction operations, which will always form a comprehensive set of representative boundaries. We call this the set of birth-cycles. Storing these during run-time can require a large amount of memory for large data-sets. We developed an algorithm that computes every cycle of this set on the fly without having to store any other cycle in memory (see Section 2 in S1 Text for details). Every computed cycle is directly written to a file.

### Shortening representative boundaries

After computing the birth-cycles, we shorten them in multiple stages as follows.

I Greedy shortening: We initialize the set of representative boundaries, $C=\{C_{i}\},$ as the set of all columns *V*(*σ*_*i*_) such that a nontrivial feature is born when *σ*_*i*_ is added to the simplicial complex. The length of a cycle *C* is the number of simplices in it—#edges in H_1_ representatives and #triangles in H_2_ representatives. We denote it by |*C*|. The basic idea of the shortening algorithm is defined by the following steps:
  a Compute $d^{*}={\text{max}}_{C_{i},C_{j}\in C}\left\{|C_{i}⊕C_{j}|-|C_{i}|\right\}.$ If d* = 0, then exit the algorithm.
  b Replace *C*_*i*_ by *C*_*i*_ ⊕ *C*_*j*_ if |*C*_*i*_ ⊕ *C*_*j*_| − |*C*_*i*_| = d*. If for a *C*_*i*_ there are multiple such *C*_*j*_, we arbitrarily pick one to shorten *C*_*i*_.
  c Go to step a.
  A straightforward implementation of computing d* is to check |*C*_*i*_ ⊕ *C*_*j*_| for all possible pairs. However, it might not be feasible in run-time if the number of cycles is large. For example, it took around 12 hours to shorten a set of around 100,000 H_1_ representatives using this approach. We optimized the greedy algorithm into different cases (Section 3 in S1 Text for details) such that it took under 20 minutes to shorten the 100,000 H_1_ representatives. We implemented this optimization for H_1_ representatives but not for H_2_. This is because a similar optimization for H_2_ representatives will theoretically require a data structure of size *O*(*n*^3^) in the worst case. However, our aim is to ensure that all data structures are *O*(*n*^2^). The straightforward implementation for H_2_ representatives was computationally feasible in all of our examples because of the small number of H_2_ features.
II Check for connectedness: *C*_*i*_⊕*C*_*j*_ can result in disconnected cycles. We say that two H_*d*_ representatives are disconnected if they have no common *d*-simplex. After the greedy algorithm, we check for the connectedness of every cycle (see Section 4 in S1 Text for details) and record disconnected ones as separate cycles in C.
III Local smoothing: We smooth the shortened cycles by reducing them with trivial topological features. Hence, we reduce H_1_ representatives with triangles and H_2_ representatives with tetrahedrons. We develop algorithms in which we smooth a cycle by iterating over only those trivial features that share a boundary with it (see Section 5 in S1 Text for details).

The following steps presume that a spatial embedding of the data set in a Euclidean space is available.

IV Graphical contraction (see Section 6 in S1 Text for details):
  a Defining covers: For each cycle, we define its local cover as the set of points in and on the smallest hyper-rectangle in Cartesian coordinates that contains inside it all the points of the cycle.
  b Eliminating cycles that cannot be around significant holes: For a cycle we compute PH of the data set which is defined by the embedding of all the points on and inside the cover of this cycle. If the number of significant features is zero, then this cycle cannot wrap around significant features. All such cycles are ignored from subsequent analysis.
  c Reducing number of covers: We define two rules to update the collection of covers such that there is a possible decrease in the number of covers and/or a decrease in the number of points in some covers. First, if cover *A* is a subset of cover *B* and they both contain the same number of significant features, then we remove *B* from the collection of covers. Second, if intersection of two covers *A* and *B* contains the same number of significant features as *A* and/or *B*, then *A* and/or *B* is replaced by *A* ∩ *B*.
V Stochastic shortening: We add stochasticity in two different ways in each cover. In Section 7 in S1 Text we give the details and also provide a rationale for why these might make it possible to discover shorter representative cycles. Briefly, we perturb the locations of the points in the cover to generate multiple perturbed data sets. For each perturbation, we generate multiple permutations of the order of edges of the same length in the coboundary (and boundary) matrix. We implement steps (I) to (IV) for each permutation of perturbation, generating multiple, possibly distinct, sets of representative boundaries. From these sets, we pick the shortest representative boundaries.

### Hi-C analysis for control vs. auxin

We first summarize the methodology of a Hi-C experiment to understand the data obtained. Nuclear DNA from millions of cells is shredded. Pairs of spatially proximal shredded fragments have a high chance to ligate due to their inherent stickiness. Paired fragments are sequenced and counted. The counts are aggregated at a specific resolution, say *r* kb, and reported as follows. The linear chromosomes are partitioned into fixed length segments of *r* base pairs, called *bins*. The Hi-C contact matrix, *M* = [*m*_*ij*_], is constructed where *m*_*ij*_ reports the aggregated counts, or contact frequency, of paired fragments where one fragment is in bin *i* and the other is in bin *j*. The contact matrix is balanced to account for experimental biases. There are multiple methods to balance or normalize Hi-C contact matrices [36]. We implemented iterative cross-entropy (ICE) normalization using Cooler [37] balance. Fragments that are spatially close in the folded genome, are expected to have higher counts or contact frequencies. Hence, we estimated the pairwise-distance matrix by taking the multiplicative inverse of non-zero contact frequencies, i.e. $\hat{D}=\left[{\hat{d}}_{ij}=1/m_{ij}\right].$ The pairwise distance for zero contact frequencies was estimated as infinity. In other words, an edge between bin *i* and bin *j* is not added to the simplicial complex if *m*_*ij*_ = 0, regardless of the threshold of PH computation. ICE normalization resulted in *m*_*ij*_ = NaN for a few pairs. The corresponding edges were not added to the simplicial complex.

After greedy shortening, we lose information about the persistence of topological features that they wrap around. For every cycle, we estimated the theoretically maximum persistence of a feature that it can wrap around. The birth was estimated as the longest edge in the boundary and death was estimated as the maximum of all possible pairwise distances between points of the cycle. Cycles with difference between estimated death and estimated birth lower than *ϵ* were ignored.

### Hi-C analysis for different protocols

#### Data procurement and computing H_1_ loops in chr1 at 10 kb

Hi-C interaction matrices in hic format were downloaded from respective 4DN accession entries, and converted to cool files at a resolution of 10 kb, using hic2cool (https://github.com/4dn-dcic/hic2cool). The resulting single-resolution cooler files were normalized by ICE using Cooler balance using cis-only flag. Analysis was done only for pixels (*b*_*i*_, *b*_*j*_) of the Hi-C interaction matrix where both bins *b*_*i*_ and *b*_*j*_ are in chr1. Pairwise distances between genomic bins were estimated as the multiplicative inverse of the normalized reads and scaled as follows. The mean (*μ*) and standard deviation (*σ*) were computed for the distribution of estimates of bin distance equal to 1. We ignored the top 0.1%-tile of estimates in this distribution which might contain experimental outliers. Estimates were then scaled by $\tilde{x}=\left(x-\mu \right)/\sigma$ and made positive by summing with the most negative value, ${\tilde{x}}_{+}=\tilde{x}+\left|\text{min}\left\{\tilde{x}\right\}\right|.$ Next, the estimates were shifted (*c*) and scaled (*p*) using $\hat{x}={\tilde{x}}_{+}^{p}+c.$ The values of *p* and *c* were chosen to match the 5%-tiles and 95%-tiles across different experiments (see S3 Fig). The shift for all experiments was 0 and the values of *p* were 1.18, 1.05, 0.98, 1.05, and 0.84 for the experiments in Table 2, respectively. Let *D*_*k*_ be the distribution of spatial estimates for all edges (*b*_*i*_, *b*_*j*_) of an experiment where the bin-distance |*b*_*i*_ − *b*_*j*_| is *k*, and *p*_*k*_ be the 95%-tile of *D*_*k*_. Shortened H_1_ loops were computed at three different pairs of parameters (*τ*_*u*_, *τ*) for each experiment—(*p*_2_, *p*_4_), (*p*_3_, *p*_4_), and (*p*_4_, *p*_5_). See S1 Table for parameter values.

#### Computing HiCCUPS peaks at 10 kb

A HiCCUPS peak is a pair of bins *H* = (*b*_1_, *b*_2_). Multi-resolution Hi-C files for all experiments were downloaded from 4DN repository [38]. HiCCUPS peaks were computed for 10 kb resolution for each experiment using cooltools [39]. Default values and settings were used except for two changes. max_nnz was changed to 4 as used by [31]. n_lambda_bins was increased to 50 since the default value of 40 gave an error for some of the data sets. HiCCUPS peaks were considered after the default clustering and filtering methods provided in cooltools. The default value of the clustering radius is 20 kb. We used this as the threshold for matching H_1_ loops with HiCCUPS peaks, as we explain later.

#### Consistency of shortened H_1_ loops across experiments

A H_1_ loop is an ordered set of bins [*b*_0_, …, *b*_*n*_] where (*b*_*i*_, *b*_*i*+1_) is an edge in the loop. Let *A* = [*a*_*i*_]_1≤*i*≤*n*_ and *B* = [*b*_*j*_]_1≤*j*≤*m*_ be two H_1_ loops. We define the distance of *A* from *B* by $d(A,B)={\text{max}}_{a_{i}\in A}\{{\text{min}}_{b_{j}\in B}\{|a_{i}-b_{j}|\}\}$. Distance between loops *A* and *B* was computed as max{*d*(*A*, *B*), *d*(*B*, *A*)}.

#### Matching between H_1_ loops and HiCCUPS peaks

Given a H_1_ loop *A* = [*a*_*i*_]_1≤*i*≤*n*_, we define distance between a bin *b* and $d(L,b)={\text{min}}_{a_{i}\in A}\{|a_{i}-b|\}$. Following this, we define distance between *L* and *H* by *d*(*L*, *H*) = max{*d*(*L*, *b*_1_), *d*(*L*, *b*_2_)}. If *d*(*L*, *H*) ≤ 2, H_1_ loop *L* and HiCCUPS peak *H* were marked as matched.

#### Computing cCREs

A H_1_ loop or a HiCCUPS peak was said to have a cCRE if there existed any bin *b* in them such that *b* or bins adjacent to it contained at least one of DNase-only, DNase-H3K4me3, dELS, pELS, and PLS.

#### Computing biomarker enrichment

For every bin in a H_1_ loop or a HiCCUPS peak, we find open chromatin regions in the bin and bins adjacent to it. A list of all such unique chromatin regions is created using ATAC-seq information. deepTools [40] computeMatrix and plotProfile were used to compute and plot the enrichment profiles for H3K27ac, H3K4me3, and SMC1 at these regions.

#### Classifying range of interaction of H_1_ loops and HiCCUPS peaks

Maximum range of interaction of a H_1_ loop *A* = [*b*_1_, …, *b*_*n*_, *b*_0_], where (*b*_*i*_, *b*_*i*+1_) is an edge in it, was computed as max_1≤*i*≤*n*_{|*b*_*i*_ − *b*_*i*+1_|}. For a HiCCUPS peak (*b*_*i*_, *b*_*j*_), it was computed as |*b*_*i*_ − *b*_*j*_|. Short-range, mid-range, and long-range classifications were based on maximum range of interaction being less than 100 kb, at least 100 kb but less than 1 Mb, and at least 1 Mb, respectively.

#### Computing biomarker enrichment of bins in loops with single and multiple mid-range interactions

Bins were classified as mid-range if they were in at least one mid-range loop and not in any long-range loop. They were further classified as multiple mid-range if they were in at least one loop with multiple mid-range interactions, and single mid-range otherwise. Biomarker enrichment of single and multiple mid-range loops was computed by computing it for single and multiple mid-range bins and bins adjacent to them, respectively.

#### Computing H_1_ loops and HiCCUPS peaks common to all experiments

A graph *G* was defined with nodes as (*e*_*i*_, *L*_*ij*_) where *L*_*ij*_ is a H_1_ loop in experiment *i*. All loops of every experiment were represented as a node of *G*. Edge was defined between (*e*_*i*_, *L*_*ij*_) and (*e*_*k*_, *L*_*km*_) iff *i* ≠ *k* and *d*(*L*_*ij*_, *L*_*km*_) ≤ 2. A clique of size 5 of *G* then indicates loops in the five different experiment such that every pair of loops is close to each other. We computed all maximal cliques of size five of *G* and picked one node from each clique as a representative common loop.

#### Multiple-resolution analysis of chr1

Hi-C counts for the experiment with MNase were binned at 1, 5, and 10 resolutions. They were balanced with cis-only flag. Pairwise spatial distances between bins of chr1 were estimated as the multiplicative inverse of the balanced counts. They were normalized with respect to estimates of bins with bin-distance of one, as was explained previously for 10 kb resolution. For each resolution, birth-threshold was *p*_4_ and PH-threshold was *p*_5_.

### PDB analysis to find homologs with different H_2_ topology

#### Data procurement and computing PH up to and including H_2_.

Spatial coordinates of the backbone of PDB entries were extracted using the Python Prody package [41]. Birth-threshold was *τ*_*u*_ = 10 and the threshold for persistence was *ϵ* = 3.5 for PH computation. These choices were based on the fact that the average bond lengths in protein molecules range from around 1.5 Å to 3 Å. S11 Fig shows births versus persistence of all H_2_ features computed for the 174, 574 PDB entries that were processed. There exists a cluster of H_2_ features with relatively high persistence greater than 2.75 exists between birth 7 and 10. All PH computations were done on x2650 processors.

#### Computing homolog sets

Given a subject sequence, another sequence was classified as its homolog if at least 75% of the sequence length matched the subject sequence with a sequence identity score of at least 75% and the backbone lengths differed by at most 25%. Sequence search at RSCB Protein Data Bank was used to find homologs of all sequences. A script was written to automate this search using the Python package pyPDB [42]. PDB entries that had a residue entry not among the 20 amino acids, were ignored.

#### Computing tight-representatives

There were 77 covers with significant features after graphical contraction. Stochastic shortening of representative boundaries was implemented for each cover with 15 perturbations and 15 permutations per perturbation.

#### Pairwise comparison of H_2_ PDs

For each PD, a list of significant values of persistence (≥ 3.5) sorted in decreasing order was constructed. The number of entries in the two lists was equalized by padding with zeros. The maximum of element-wise difference between these two lists was defined as the L_0_ distance between the two PDs. Two PDs, either one or both containing at least one significant feature, were classified as significantly different if their L_0_ distance was greater than 3. L_0_ distance was compared with Bottleneck, Wasserstein, and sliced Wasserstein distances using persim-0.3.1 Python package.

## Supporting information

S1 Appendix Background and terminology for computing PH using matrix reduction algorithm. (PDF)

S1 Text Details of strategy and algorithms. Explanation of each step of our strategy and pseudocode of our algorithms.
(PDF)

S1 Table Hi-C experiments with different protocols. Data sets of the case-study comparing Hi-C experiments with different protocols. Parameters for PH computation after ICE normalization and rescaling—PH thresholds (*τ*_*i*_) and birth-thresholds (*τ*_*u*,*i*_) to compute tight representatives.
(PDF)

S1 Fig Spatial pairwise estimates from Hi-C matrices provide reasonable estimates of the distance between adjacent bins on a chromosome. (A) Distribution of pairwise estimates for a bin distance of 1 is significantly lower as compared to those of higher bin distances. (B) Means and medians of the distributions increase with an increase in bin distances.
(PDF)

S2 Fig Lengths of cycles decrease at every stage of the algorithm. (A) Interquartile lengths of the set of representative boundaries decreased by multiple log scales using the greedy shortening algorithm. (B) Local smoothing reduces some boundaries to degenerate cycles of length two as is shown by the lowermost humps in the distribution of smooth cycles.
(PDF)

S3 Fig Spatial estimates from balanced Hi-C counts. (A) Distributions of spatial estimates for different genomic distances for experiment 1. (B) 5%-tile and 95%-tile of distribution of spatial estimates at different bin distances. Left panel is before scaling and right panel is after scaling.
(PDF)

S4 Fig Consistency of H_1_ loops, computed at largest threshold, across different experiments. Distances of loops of experiment *i* from loops of experiment *j* were computed for 1 ≤ *i*, *j* ≤ 5. Vertical dashed line shows threshold of 1 bin-distance between loops. Plots (A) to (E) show cumulative percentages of loop-distances computed for experiments 1 to 5 with every other experiment.
(PDF)

S5 Fig Consistency of tight H_1_ at different thresholds. Consistency of computed tight H_1_ loops across different experiments 1 to 5 (left to right) at (*τ*_1_, *τ*_*u*,1_) (top row) and (*τ*_2_, *τ*_*u*,2_) (bottom row).
(PDF)

S6 Fig H_1_ loops and HiCCUPS peaks overlaid on Hi-C interaction matrix. HiCCUPS peaks (teal dots) that do not match a H_1_ loop (black lines) have a lower balanced Hi-C frequency as compared to those that match.
(PDF)

S7 Fig Multiple resolution analysis of H_1_ loops computed in chr1 for experiment 5. The majority of loops in all but one category contain cCREs.
(PDF)

S8 Fig Spatial estimates at 1 kb resolution for Hi-C experiments with different protocols. Distributions of spatial estimates at different bin-distances for experiments 1 to 5 (panel A to E). Distributions for MNase (panel E) are more distinct at lower bin-distances as compared to other experiments.
(PDF)

S9 Fig Maximum, median, and minumum of distributions of computation times binned at unique data set sizes. (A) PD computation times of 174, 574 PDB entries up to threshold of 13.5 Å. (B) PD and tight representation computation times for all significant voids identified in 25 homolog sets.
(PDF)

S10 Fig Comparing Bottleneck, Wasserstein, and sliced Wasserstein with L_0_ metric. (A) Comparing different metrics for H_2_ PDs of pairwise homologs. L_0_ norm agrees with the bottleneck distance. (B) Comparing computation times with respect to L_0_ norm computation. L_0_ metric agrees with Bottleneck distance and is computationally faster by multiple orders or magnitude.
(PDF)

S11 Fig Persistence versus birth of H_2_ features in 174, 574 PDB entries. A cluster of features with relatively high persistence exists between 7 ≤ birth ≤ 10. These features have persistence at least 2.75, shown by the lower dashed red line. Our choice of *ϵ* = 3.5 is shown by the upper dashed red line.
(PDF)

S12 Fig Homologs with significantly different H_2_ topology. Black labels are the number of significant H_2_ features in every PDB in that column.
(PDF)

S13 Fig Computed tight voids in all homologs with significantly different H_2_ topology. (PDF)
