# Supplementary material for: Tight basis cycle representatives for persistent homology of large biological data sets
Source: PLoS Comput Biol. 2023 May 30;19(5):e1010341. doi: 10.1371/journal.pcbi.1010341 (PMC10275456; doi:10.1371/journal.pcbi.1010341)
Supplement: S1 Appendix — (PDF) [file pcbi.1010341.s001.pdf]

1 Background and terminology

Symbol/Term	Description
n-simplex	A set of $n+1$ points
vertex	A set of a single point, 0-simplex
edge	A set of two points, 1-simplex
triangle	A set of three points, 2-simplex
tetrahedron	A set of four points, 3-simplex
boundary ($\partial\sigma$)	All $(n-1)$-simplices that are in the n-simplex σ
coboundary ($\delta\sigma$)	All n-simplices boundaries of which contain the $(n-1)$-simplex σ
diameter of a simplex	Longest edge in it
H_d	Dimension d homology group
H_d^*	Dimension d cohomology group
D	Boundary matrix
V	Matrix that records operations to reduce boundaries
R	Matrix with reduced boundaries
simplicial complex	A set of simplices
filtration (F)	Sequence in which simplices are added to the complex
F_d	Sequence of d-simplices in the filtration
$D^\perp$	Coboundary matrix
$V^\perp$	Matrix that records operations to reduce coboundaries
$R^\perp$	Matrix with reduced coboundaries
$p^\perp$	List of persistence pairs from cohomology computation

Table A: Some basic terminology, symbols, and descriptions.

Given a set of $k+1$ discrete points in Euclidean space $\mathbb{R}^d$, simplicial complexes are used to construct topological structures on them. The following definitions are taken from [1]. A point $x = \sum_{i=0}^{k} \lambda_i u_i$ is an *affine combination* of the points if λ_i sum to 1. The *affine hull* is the set of affine combinations. The $k+1$ points are affinely independent iff the k vector $u_i - u_0$, for $1 \leqslant i \leqslant k$, are linearly independent. An affine combination, $x = \sum \lambda_i u_i$, is a *convex combination* if all λ_i are non-negative. The *convex hull* is the set of all convex combinations. A *k-simplex* is the convex hull of $k+1$ affinely independent points, $\sigma = \mathrm{conv}\{u_0, u_1, ..., u_k\}$. Its dimension is $\dim \sigma = k$. The first few dimensions have special names, *vertex* for 0-simplex, *edge* for 1-simplex, *triangle* for 2-simplex, and *tetrahedron* for 3-simplex. Any subset of affinely independent points is also affinely independent, and therefore a simplex. A *face* of σ is the convex hull of a non-empty subset of the $k+1$ points (denoted by $\tau \leqslant \sigma$) and it is *proper* if it is not the entire set (denoted by $\tau < \sigma$). A set of size $k+1$ has 2^{k+1} subsets, and hence $2^{k+1}-1$ faces (excluding the empty set). The *boundary* of σ, denoted by bd σ is the union of all proper faces, and the *interior* is everything else, int $\sigma = \sigma - \mathrm{bd}\,\sigma$. A point $x_i = \sum \lambda_i u_i \in \mathrm{int}\,\sigma$ iff all λ_i are positive.

A *simplicial complex* is a finite collection of simplices K such that $\sigma \in K$ and $\tau \leqslant \sigma$ implies $\tau \in K$, and $\sigma, \sigma_0 \in K$ implies $\sigma \cup \sigma_0$ is either empty or a face of both. The motivations behind this definition are to have sets of simplices that are closed under taking faces and have no improper intersections. The dimension of K is the maximum dimension of any of its simplices. The *underlying space*, denoted by $|K|$, is the union of its simplices together with the topology inherited from the ambient Euclidean space in which the simplices live. A *triangulation* of a topological space X is a simplicial complex K together with a homeomorphism between $\mathbb{X}$ and $|K|$. A subcomplex of K is a simplicial complex $L \subseteq K$. The *diameter* of a simplex is the supremum over all distances between its points, in the Euclidean space in which it lives. The *mesh* of K is the maximum diamter of any simplex or, equivalently, the length of its longest edge.

In this work, we construct *Vietoris-Rips* complexes (VR-complex) on the point-cloud. At a spatial scale of τ, the VR-complex is defined as the collection of all simplices with diameter at most τ. Topologically distinct loops and voids in a simplicial complex are computed as basis elements of its homology groups H_1 and H_2, respectively [2]. Persistent homology computes changes in the number of basis elements as the spatial scale of observation, τ, increases [3]. These changes are represented as *birth* and *death* of basis elements of the

homology groups. A (birth, death) pair is also called a persistence pair. Nontrivial persistence pairs are plotted with birth along the x-axis and death along the y-axis. These plots are called persistence diagrams (PD). A feature with higher *persistence* = death − birth, will be robust to larger variability in the data set.

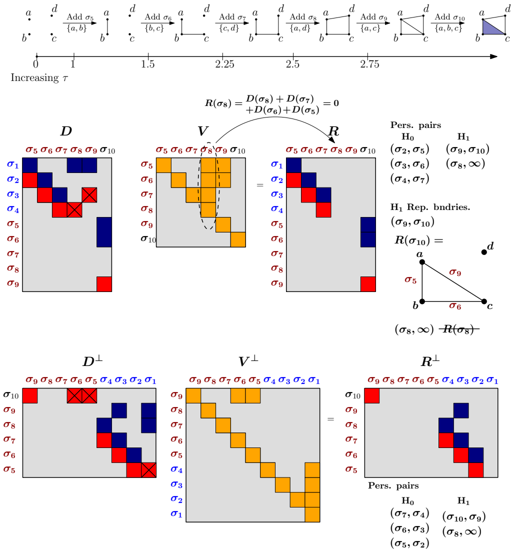

Figure A: Illustrating matrix reduction algorithm.

The matrix reduction algorithm [4, 3] is an established method to compute PD. It also yields a set of representative boundaries. We briefly describe the method and refer to Fig. A as an example. Simplices are denoted by σ_i, where $1 \leqslant i \leqslant N$ is the index of the simplex. They are indexed in the order in which they are added to the simplicial complex. Hence, they are indexed in the order of increasing diameters. Those with the same diameter are assigned unique indices arbitrarily. The *boundary matrix* D is constructed for a simplicial complex as follows. Row and column i of D correspond to simplex σ_i in the simplicial complex. Column i of D has 1 at the boundary-simplices of σ_i (blue boxes in A), and is 0 (gray boxes) otherwise. Boundary-simplices of a n-simplex are all $(n-1)$-simplices that can be constructed using its points. We denote the column corresponding to a simplex σ_i in a matrix M by $M(\sigma_i)$. Every non-zero column of D has a lowest non-zero element (shown by red boxes), called a *low*. The matrix reduction algorithm then dictates to *reduce* columns of D, such that each row of D has at most one low (red box). Each reduction operation is a sum of columns mod p, where p is a prime number. In this work, $p = 2$.

The reduction of D is specifically from left to right as follows. A column of j of D is reduced only when all columns $i < j$ have been reduced. Also, column j is reduced only with a column $i < j$. This is written as a matrix multiplication, $DV = R$, where V records the reduction operations for columns of D that result in the reduced matrix R. Note that V is always an upper triangular matrix because reductions operations are from left to right. There are two kinds of persistence pairs that are determined from R. First, a low (red box) in R at (i, j) implies that a feature was born when σ_i was added to the simplicial complex and it died when σ_j was added. This persistence pair is denoted by (σ_i, σ_j). It is also called a pivot element of R. Also, $R(\sigma_j)$ is a representative homology cycle for this feature. Second, if $R(\sigma_i) = \mathbf{0}$ and σ_i is not in any low of R (σ_8 in the example), then the persistence pair (σ_i, ∞) is a feature that was born when σ_i was added, but it does not die. Such features might exist when PH is computed up to a spatial scale that is less than the maximum of all pairwise distances. There is no representative boundary in R for such a feature.

Another way to compute exactly the same PD is by reducing the coboundary matrix, $D^\perp$. It is the transpose of the secondary diagonal of D. Hence, column and row i of $D^\perp$ correspond to simplex σ_{N-i+1}. Similar to the reduction of boundary matrix, the coboundary matrix $D^\perp$ is also reduced from left to right and written as $D^\perp V^\perp = R^\perp$. Persistence pairs from $R^\perp$ and R have a bijective mapping [5]. If (σ_j, σ_i) is a pivot of $R^\perp$, then (σ_i, σ_j) is a pivot of R. The pair (σ_i, σ_j) is a persistence pair with birth when σ_i is added to the complex and death when σ_j is added to the complex. If $R^\perp(\sigma_i) = \mathbf{0}$ and there is no pivot in row for σ_i, then $R(\sigma_i) = \mathbf{0}$ and there will be no pivot in the row for σ_i in R. Hence, the pair (σ_i, ∞) is a feature

that does not die. In the example there are two H_1 features, $(\sigma_9, \sigma_{10}) \equiv (2.75, 2.75)$ and $(\sigma_8, \infty) \equiv (2.5, \infty)$. Non-zero columns of R form a set of representative boundaries. In the example, $\{R(\sigma_{10}) = \{\sigma_5, \sigma_6, \sigma_9\}\}$ is the only H_1 representative homology boundary. However, there are two H_1 features. Hence, columns of R do not form a comprehensive set of boundaries when there are features that do not die.

References

1. Edelsbrunner H, Harer J. Computational topology: an introduction. American Mathematical Soc.; 2010.
2. Hatcher A. Algebraic topology. Citeseer; 2001.
3. Edelsbrunner H, Harer J. Persistent homology-a survey. Contemporary mathematics. 2008;453:257–282.
4. Carlsson G, Zomorodian A. Computing persistent homology. Discrete Comput Geom. 2005;33(2):249–274.
5. De Silva V, Morozov D, Vejdemo-Johansson M. Dualities in persistent (co) homology. Inverse Problems. 2011;27(12):124003.
