# Supplementary material for: Tight basis cycle representatives for persistent homology of large biological data sets
Source: PLoS Comput Biol. 2023 May 30;19(5):e1010341. doi: 10.1371/journal.pcbi.1010341 (PMC10275456; doi:10.1371/journal.pcbi.1010341)
Supplement: S1 Text — Explanation of each step of our strategy and pseudocode of our algorithms. (PDF) [file pcbi.1010341.s002.pdf]

1 Computing homology

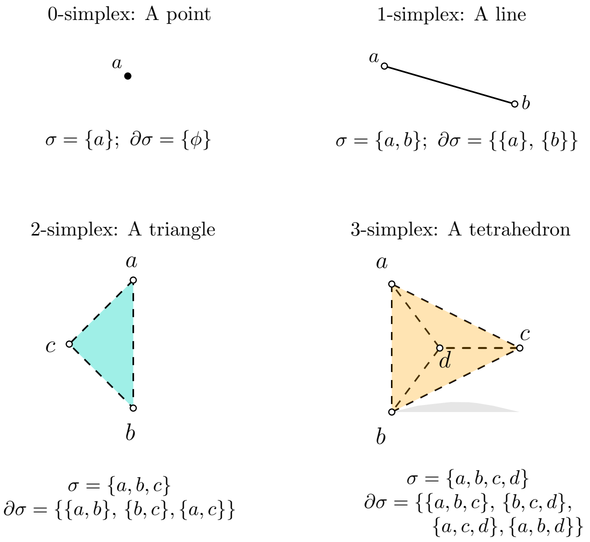

Figure A: Simplices

We first compute the persistence pairs by reducing the coboundary matrix and store them as $p^\perp$. From duality between homology and cohomology, it follows that we need to reduce a column $D(\sigma_i)$ only if (σ_i, σ_j) is a persistence pair in $p^\perp$. Additionally, we provide a way to efficiently determine apparent pairs [1], do not require any reduction. Hence, we reduce column $D(\sigma_i)$ only if (σ_i, σ_j) is a persistence pair in $p^\perp$ and is not a trivial persistence pair.

1.1 Apparent pairs

Suppose t is the smallest triangle in the coboundary of an edge e. Additionally, if e is the diameter of t, then it will be the lowest edge in $D(t)$. Further, since t is the smallest triangle in the coboundary of e, there is no triangle smaller than t that has e in its boundary. Hence, all entries in row e and to the left of column t will be 0. As a result, the lowest entry in column t is the first non-zero entry in row e, making it a pivot entry (first low in the row from left). Hence, column t will not require any reduction operations, and we say that (e, t) is an apparent persistence pair in H_1.

Now, we will show that if (e, t) is an apparent persistence pair in H_1, then (t, e) is a persistence pair in H_1^* such that coboundary of e does not require any reduction operations. Since t is the lowest triangle in the coboundary of e, all entries below row t in column e of the coboundary matrix will be 0. Also, since e is the diameter of t, all entries in row t to the left of column e will be 0, making (t, e) a pivot entry. As a result, column e in the coboundary matrix will not require any reduction operations, and we say that (t, e) is an apparent persistence pair in H_1^*. In Dory, we compute cohomology and store the persistence pairs that are not apparent in $p^\perp$. $p^\perp$. Therefore, to compute H_1, we need to iterate only over the triangles that are in some persistence pair in $p^\perp$ because these triangles are also the ones that are not in an apparent persistence pair of H_1.

Since we do not store apparent persistence pairs, we have to check for them at every reduction step as well. Suppose e is the lowest edge in the partially reduced boundary of a triangle. Then, if (e, t') is an apparent persistence pair, the next reduction has to be with the boundary of t'. So, our aim is to determine whether there is an apparent persistence pair (e, t') for a given edge e. To do so efficiently, we use paired-indexing that we introduced in [2]. Paired-indexing encodes a simplex such that it stores information about its diameter. A triangle t is stored as $\langle k_p, k_s \rangle$ where k_p is its diameter and k_s is the third vertex in the triangle that is not in the edge corresponding to its diameter. Then, for an edge e, if $t' = \langle e, k_s \rangle$ is the lowest triangle in the coboundary of e, then (e, t') is an apparent persistent pair. These checks are computationally feasible

because the number of reduction steps is lowered on account of processing only the triangles that are in some persistence pair in $p^{\perp}$.

Similarly, to compute H_2 we iterate over the tetrahedrons that are in some persistence pair in $p^{\perp}$. As before, we have to check for apparent persistence pairs during reduction because we do not store them in computer memory. Suppose t is the lowest triangle in partially reduced boundary of a tetrahedron. Then, if (t, h') is an apparent persistence pair, the next reduction has to be with the boundary of h'. If h' is the smallest tetrahedron in the coboundary of t and the maximum triangle in boundary of h' is also t, then (t, h') is an apparent persistent pair, and the next reduction has to be with the boundary of h'.

1.2 Final algorithm for homology computation

We reduce the boundaries of simplices in batches using the serial-parallel reduction that we introduced in [2] (SI Figure B). The default batch-size for parallel reduction of boundaries is chosen to be 1000.

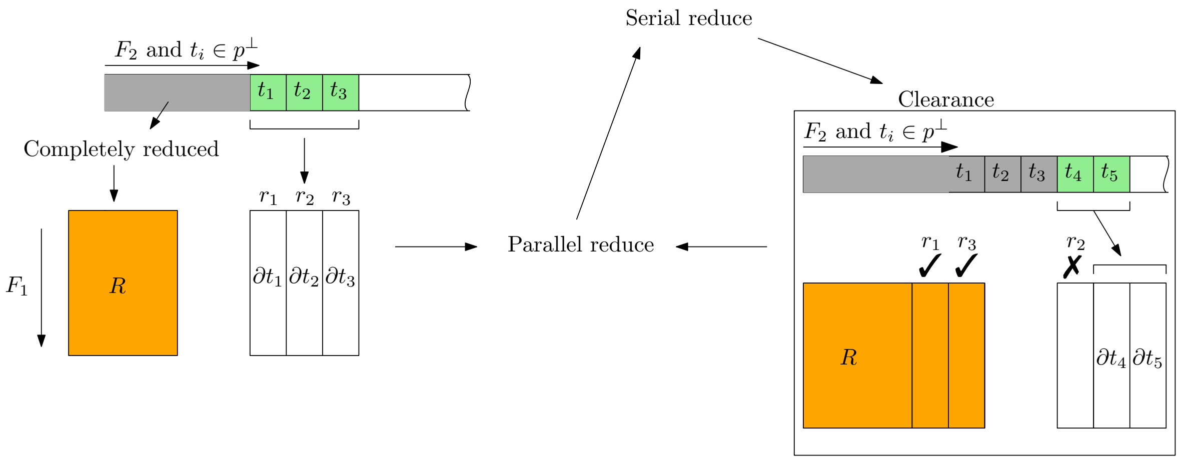

Figure B: Serial-parallel reduction to reduce boundaries of triangles. Only those triangles that are in some persistence pair in $p^{\perp}$ need to be considered for reduction.

2 Recursive algorithm to compute columns of V

We develop a recursive algorithm that computes $V(\sigma)$ without having to store V. We also show that by selectively storing a few columns of V, we can significantly reduce the computation time at a very low cost of additional memory.

2.1 Computing birth-cycles for H_1

We show how we use R to reduce the boundary of any edge e_o in the simplicial complex on the fly. We consider two cases, $R(e_o)$ is non-empty (or non-zero) and $R(e_o)$ is empty (or zero). We first go over the former case since the latter will require the former. The function `FindV` in algorithm 1 computes $V(e_o)$ when $R(e_o)$ is non-empty. We begin by defining $r_{e_o} = \partial e$ and $V(e_o) = [\,]$. We reduce r_{e_o} with R by updating $r_{e_o} \leftarrow r_{e_o} \oplus R(e')$ if $\text{low}(R(e')) = \text{low}(r_{e_o})$ and append e_o to $V(e_o)$. However, we have to append to $V(e_o)$ the reduction operations corresponding to $R(e')$, that is, $V(e')$. This sets up a recursion to compute $V(e')$. It remains to define the termination of recursion. We show that ∂e_o will always reduce to zero. Initially, $r_{e_o} = \partial e_o$. Since $R(e_o)$ is the complete reduction of ∂e_o that exists in R, we know that reduction of ∂e_o with R will eventually make it equal to $R(e_o)$. Hence, $r_{e_o} = R(e_o)$ at some point in the reduction, and the next reduction step will be summing r_{e_o} and $R(e_o)$ because they have the same low. This will result in zero.

We now tackle the latter case of $R(e_o)$ empty. Initially, $r_{e_o} = \partial e_o$. In contrast to the former case, $V(e_o)$ is initialized with $[e_o]$ and is not empty. We reduce r_{e_o} with R by updating $r_{e_o} \leftarrow r_{e_o} \oplus R(e')$ if $\text{low}(R(e')) = \text{low}(r_{e_o})$. Since $R(e')$ will be a non-empty column in R, we use `FindV` (algorithm 1) to compute reduction

Algorithm 1 Compute $V(e_o)$ for edge e_o with $R(e_o)$ non-empty

Input R, e_o
Output $V(e_o)$
$V(e_o) \leftarrow [\,]$
`FindV`$(e_o, V(e_o))$

function `FindV` $(e, V(e_o))$
    Append e to $V(e_o)$
    $r_e \leftarrow \partial e$
    while r_e not empty **do**
        if low$(R(e'))$ = low(r_e) **then**
            $r_e \leftarrow r_e \oplus R(e')$
        if r_e is NOT empty **then**
            `FindV`$(e', V(e_o))$

operations required to reduce $\partial e'$ to $R(e')$ and append them to $V(e_o)$. The reduction ends when r_{e_o} inevitably reduces to zero since $R(e_o)$ is empty. We do not keep track of the coefficients of edges in $V(e_o)$ during reduction to reduce computation time. Hence, we remove edges with zero coefficient (modulo 2) from $V(e_o)$ at the conclusion of recursion since their boundaries will sum to 0, at the end of the reduction loop.

Algorithm 2 Compute $V(e_o)$ for edge e_o with $R(e_o)$ empty

Input R, e_o
Output $V(e_o)$
$V(e_o) = [e_o]$
$r_{e_o} \leftarrow \partial e_o$
while r_{e_o} is not empty **do**
    if low$(R(e'))$ = low(r_{e_o}) **then**
        $r_{e_o} \leftarrow r_{e_o} \oplus R(e')$
        `FindV`$(e', V(e_o))$
Remove edges from $V(e_o)$ with zero coefficient

2.2 Computing birth-cycles for H_2

The algorithm to compute birth-cycles for H_2 is written similarly by recursively reducing the boundaries of triangles at which topological features are born. However, we do not store apparent persistence pairs of H_1 in R, and compute these on the fly. Also, if (e', t') is an apparent persistence pair, we know that $R(t') = \partial t'$. Hence, we do not compute $V(t')$ in such cases (algorithm 3).

2.3 Selective storage for computational optimization

V does not need to be stored in memory in the recursive algorithm. We write each birth-cycle to a file when it is computed. The only additional memory taken is the maximum possible size of $V(\sigma)$. However, recursive reduction can be expensive depending upon the number of reductions and the recursion-depth of every reduction. To alleviate this problem, we note that it is possible for a column $R(\sigma_i)$ to be used multiple times in the recursive computation of birth-cycles. In this case, storing its reduction operations, $V(\sigma_i)$, in computer memory can decrease the computational overhead. It is also possible that the time taken to compute $V(\sigma_i)$ is low and the multiple computations have a negligible computational overhead. Hence, we developed selective storage of columns of V based on two criteria. For notational convenience, we drop the subscript i. First, we consider the number of reductions, inclusive of the reductions carried out recursively, required to compute $V(\sigma)$. We denote this parameter by $n_r(\sigma)$ (#reductions). Second, we maintain a counter

Algorithm 3 Compute $V(t_o)$ for triangle t_o with $R(t_o)$ empty

Input R, t_o
Output $V(t_o)$
$V(t_o) = [t_o]$
$r_{t_o} \leftarrow \partial t_o$
while r_{t_o} is not empty **do**
    if $(\text{low}(r_{t_o}), t')$ is a triv. pers. pair **then**
        $r_{t_o} \leftarrow r_{t_o} \oplus \partial t'$
        Append t' to $V(t_o)$
    else if $\text{low}(R(t')) = \text{low}(r_{t_o})$ **then**
        $r_{t_o} \leftarrow r_{t_o} \oplus R(t')$
        `FindV`$(t', V(t_o))$
Remove triangles from $V(t_o)$ with zero coefficient

function `FindV` $(t, V(t_o))$
    Append t to $V(t_o)$
    $r_t \leftarrow \partial t$
    while r_t not empty **do**
        if $(\text{low}(r_t), t')$ is triv. pers. pair **then**
            $r_t \leftarrow r_t \oplus \partial t'$
        else if $\text{low}(R(t')) = \text{low}(r_t)$ **then**
            $r_t \leftarrow r_t \oplus R(t')$
        if r_t is NOT empty **then**
            `FindV`$(t', V(t_o))$

for the number of times $V(\sigma)$ is requested as the birth-cycles are being computed. We denote this parameter by $n_u(\sigma)$ (#usage). If $n_r(\sigma) > n_r^*$ and when $n_u(\sigma)$ surpasses a user-defined threshold, denoted by n_u^*, then we store $V(\sigma)$ in the memory. Note that an optimal value of n_u^* that can provide the best trade-off between memory usage and run-time can be determined only after computing all of the birth-cycles recursively and analyzing usage of $V(\sigma)$. We chose $n_u^* = 2$ as its default value, and it can be increased if storage of fewer $V(\sigma)$ is desired and the resulting increase in computation time is insignificant.

The benefits of the above strategy will be most significant if n_r is low for most simplices and the ones with large n_r also have a large n_u. The former is desirable because it will reduce the memory taken by selective storage, and the latter means the reduction in computation time by using selective storage will be significant.

3 Greedy algorithm to compute a set of shorter cycles

A birth-cycle corresponding to a topological feature that is born in homology group H_d is a set of d-simplices. We will denote the set of all birth-cycles computed for H_d by $\mathcal{B}_d$. For notational convenience, we drop the subscript d since the analysis follows similarly in any dimension. We denote a birth-cycle by $X_i \in \mathcal{B}$ where X_i is a set of simplices. We define the length of X_i as the number of simplices in X_i, denoted by $|X_i|$. Since the set of birth-cycles forms a basis for the corresponding homology group, we propose a Gram-Schmidt like method to reduce the lengths of cycles. We substitute a cycle X_i by $X_i \oplus X_j = (X_i \cup X_j) \backslash (X_i \cap X_j)$ if such a substitution reduces its length by r_m, the maximum possible reduction in length when all pairs of cycles are considered. The iterations are continued till the sum of no two cycles results in a cycle of shorter length. We know that this condition will be satisfied eventually because every iteration results in a reduction in length of at least one cycle and the lengths are bounded below by 0. Note that X_j is possibly one of multiple cycles that can be added to X_i to reduce its length by r_m. A choice of a different cycle might result in a different set of cycles at the end of the algorithm that may be shorter. Hence, it is not guaranteed that a single run of this algorithm to termination will result in cycles of minimal lengths, and the only condition satisfied is that sum of no two cycles will give a cycle of shorter length. We also note that $X_i \oplus X_j$ might

result in disconnected cycles.

A brute-force implementation that scans all possible pairs is computationally infeasible. N cycles will require $O(N^2)$ comparisons in every iteration and every comparison involves determining $(X_i \cup X_j)\backslash(X_i \cap X_j)$, which can be costly for long cycles. Hence, such a brute-force approach is not feasible to process a large number of cycles.

We make the following observations.

1. If $|X_i| - |X_i \oplus X_j| > r_i$, then both $|X_i|$ and $|X_j|$ have to be greater than r_i.

2. If $|X_i| \geqslant |X_j|$, then $|X_i| - |X_i \oplus X_j| \geqslant |X_j| - |X_i \oplus X_j|$.

To utilize these observations, we index the birth cycles in decreasing order of their lengths. Hence, if $i < j$, then $|X_i| > |X_j|$. Indices of cycles of the same length are defined arbitrarily by the sorting algorithm. Since the relative lengths of cycles might change in an iteration, a re-indexing of cycles by sorting them in decreasing order of lengths is done at every iteration. Then, from observation 2 it follows that for a given a cycle X_i, we only need to consider substitution of X_i by $X_i \oplus X_j$ if $j > i$. Hence, we decrease the number of comparisons required in every iteration.

We begin by initializing two data structures. First, for every cycle X_i we store $f(X_i) = (X_*^i, r_i)$, where X_*^i is the cycle such that updating X_i by $X_i \oplus X_*^i$ decreases its length the most, and the decrease in length is stored as r_i. Second, for every simplex σ, that is in at least one cycle, we maintain a list of all cycles that contain it. We denote it by $g(\sigma) = [X_{i_1}, X_{i_2}, ...]$.

To initialize $f(X_i)$, we first set $r_i \leftarrow 0, X_i = \text{NULL}$, and consider the sum of X_i with all cycles X_j with $j > i$. The following ideas reduce the number of comparisons necessary.

1. If $|X_i| - |X_i \oplus X_j| > r_i$, we update $X_*^i \leftarrow X_j$ and $r_i \leftarrow |X_i| - |X_i \oplus X_j|$. We increment j by 1 and repeat till either $j = N$ or $X_j < r_i$. The latter condition follows from observation 1 because, if $X_j < r_i$, then summing X_i with any cycle X_k for $k \geqslant j$ cannot result in a reduction in length greater than r_i. Since the cycles are indexed by decreasing lengths, we can quit when $X_j < r_i$. This potentially reduces the number of comparisons to be done. Note that if $r_i = 0$ upon termination, then there is no cycle that can be added to X_i to reduce its length.

2. To compute $|X_i \oplus X_j|$, we iterate over elements in X_i and X_j and count the elements that are in either but not in both. To make this operation of order $O(|X_i| + |X_j|)$, we store every cycle as a list of simplices ordered by their index. We quit when the count is more than $|X_i| - r_i$ because that implies that the reduction in length will be less than r_i. In such a case, we do not iterate over all elements in X_i and X_j, reducing the computation time.

We compute the initial $f(X_i)$ for all birth-cycles in parallel using `OpenMP` for-loop with `static` scheduling and a `chunksize` of 1000.

We start the iterative algorithm after $f(X_i)$ and $g(\sigma)$ have been initialized. We denote the maximum r_i in $f(X_i)$ by r_m. If $r_m = 0$, the algorithm terminates. Otherwise, all X_i with $r_i = r_m$ are substituted with $X_i \oplus X_*^i$. For every substitution, $g(\sigma)$ has to be updated as follows—X_i is to be added to $g(\sigma)$ for all $\sigma \in X_*^i \backslash (X_i \cap X_*^i)$ and it has to be removed from $g(\sigma)$ for all $\sigma \in X_i \cap X_*^i$. This information is tracked during the summation of cycles as a list $u(\sigma) = [(X_{i_1}, f_1), ...]$, where $f_1 = 0$ if X_{i_1} has to be removed from $g(\sigma)$ and, otherwise, $f_1 = 1$. Then, $g(\sigma)$ is updated in parallel.

After these substitutions, we update the indices of cycles by re-sorting them in decreasing order of lengths. We also have to determine whether $f(X_i)$ has changed. To reduce the number of comparisons, we consider four different cases for every cycle X_i as follows.

1. X_i was updated: If X_i was updated in this iteration, then we reset $r_i \leftarrow 0$ and check the sum of X_i with each cycle X_j where $j > i$.

2. X_i was not updated, $r_i = 0$: This means that prior to the updates, there was no cycle that could be added to X_i to result in a cycle of shorter length. Hence, after the updates, we need to check the sum of X_i with only the cycles that were updated.

3. X_i was not updated, $r_i \neq 0$, X_*^i was updated: Since X_*^i has been updated, we check the sum of X_i with each cycle X_j where $j > i$.

4. X_i was not updated, $r_i \neq 0$, X_*^i was not updated: In this case we only need to check the sum of X_i with the updated cycles to determine if a reduction in length more than r_i is possible.

The above four cases can be categorized into two scenarios—checking the sum of X_i with all cycles and checking the sum of X_i with only the cycles that were updated in the iteration. These scenarios are implemented using different strategies.

Strategy 1 to check the sum of X_i with all cycles (cases 1 and 3): For every simplex σ in X_i, we iterate over $g(\sigma)$. Then, if $X_j \in g(\sigma)$, we know that $\sigma \in X_i \cap X_j$. Hence, we can compute $|X_i \cap X_j|$ on the fly by initializing it as 0 and increment it by 1 whenever X_j is in $g(\sigma)$ for $\sigma \in X_i$. Since $|X_i \oplus X_j| = |X_i| + |X_j| - 2|X_i \cap X_j|$, we can compute the reduction in length on the fly when X_i is summed with X_j as well. If this reduction is greater than r_i, we update $r_i \leftarrow |X_i \oplus X_j|$ and $X_*^i \leftarrow X_j$.

Strategy 2 to check the sum of X_i with updated cycles (cases 2 and 4): Suppose the $U = [X_{i_1}, X_{i_2}, ...]$ is the list of updated cycles ordered in decreasing order of their lengths. Then, we sum X_i with cycles in U as they are ordered in U. If we get a reduction in length of X_i that is greater than r_i, we update r_i and X_*^i accordingly. For efficiency, we implement the two ideas, 1 and 2, from initialization. Hence, we may need not compare X_i with all cycles in U and also may not need to iterate over all simplices in the two cycles that are being summed.

Strategy 1 and strategy 2 are implemented as embarrassingly parallel over all cycles and updated cycles, respectively. The four cases (case 1 to 4) are implemented separately in parallel for better load balance. We use `OpenMP` for-loop with `static` scheduling and a `chunksize` of 50.

Why do we use two different strategies? The strength of strategy 1 lies in the fact that iterating over $g(\sigma)$ compares only those cycles that have at least one simplex in common. However, it also means that we iterate over every simplex in every cycle X_i that is to be processed. In our experiments with test data sets, we observed that in the first few iterations almost all of the cycles are in cases 2 and 4 and only a few cycles updated. Hence, strategy 1 takes long because it iterates over the entire length of every cycle. As an alternative, we implement strategy 2 that iterates over pairs of cycles and checks their sums. It is significantly faster than strategy 1 because the ideas 1 and 2 reduce the number of pairs that are considered. Now, as iterations of the algorithm reduce the lengths of the cycles, there is a decrease in both the number of simplices in the cycles and the number of cycles that have common simplices. As a result, combined with strategy 1, the algorithm scales efficiently for a large number of cycles. The efficiency is higher if the iterations of the algorithm are accompanied with an increase in the number of cycles in cases 1 and 3.

This algorithm can be implemented for both H_1 and H_2 birth-cycles. However, since the memory requirement for $g(\sigma)$ can be $O(n^3)$ in the worst case, we implement it for only H_1. To shorten H_2 birth-cycles, we simply iterate over all pairs of nontrivial cycles in every iteration. This was computationally feasible for all of the data sets in this work because the number of nontrivial H_2 features was very low.

4 Connectedness

For every shortened H_1 representative, we compute its cycle basis using the networkx Python package and record different basis elements as separate cycles. For every shortened H_2 representative, we compute a connectivity graph, $G = (V, E)$, with the set of nodes V as all triangles in the representative and an edge between nodes is defined if the corresponding triangles have two points in common. We record disconnected components of G as separate H_2 representatives.

5 Smoothing

An H_1 representative can be written as a sequence of 0-simplices, $[v_0, ..., v_n, v_0]$. To smooth it, we simply remove v_i if the pairwise distance between v_{i-i} and v_{i+1} is at most τ_u.

An H_2 representative boundary is a set of triangular faces, $B = \{t_i\}$, and each triangle, t_i, is a set of its three vertices. We define a graph G with set of nodes $V = \{t_i\}$ and set of edges $E = \{(t_i, t_j)$ where $|t_i \cap t_j| = 2\}$,

i.e. an edge between two nodes of G denotes that the corresponding triangular faces share two vertices. For each triangle t_i, we define its diameter as the length of the longest edge in it, denoted by d_i.

Suppose a tetrahedron $h = \{t_1, t_2, t_3, t_4\}$ exists in the embedding, with birth $\leqslant \tau_u$, such that exactly three of its four faces are in B. Then, we remove those three faces from B, add the fourth face of h to B, and add edges between the fourth face and neighbors of the three removed faces. If there are multiple such possibilities, then the algorithm greedily picks a tetrahedron with smallest diameter. We update G and repeat till no update to G is possible. At the end of the iterations, we remove any disconnected tetrahedrons in B. It is not efficient to loop through all valid (with birth $\leqslant \tau_u$) tetrahedrons in the embedding of the entire data set. We developed an algorithm that, instead, is of the order of number of triangular faces in B (Algorithm 4).

Algorithm 4 Smoothing a 2-cycle

```
Input: Boundary $B = \{t_i\}$ (set of triangular faces), birth threshold $\tau_u$
Make graph $G \leftarrow (B, E)$, where $(t_i, t_j) \in E$ if they share two points.
update ← 1
while update do
    update ← 0
    $d_* \leftarrow \infty$
    $\mathcal{C} \leftarrow$ cliques in $G$ with at least 3 nodes/triangular faces
    for $C$ in $\mathcal{C}$ do
        for $(t_1, t_2, t_3)$ in combinations of 3 from $C$ do
            all points $P = t_1 \cup t_2 \cup t_3$
            $t_4 \leftarrow P \backslash \{t_1\}$
            $t_4 \leftarrow t_4 \cup (P \backslash \{t_2\})$
            $t_4 \leftarrow t_4 \cup (P \backslash \{t_3\})$
            if $t_4 \in C$ then
                Skip because tetrahedron $\{t_1, t_2, t_3, t_4\} \in B$
            else
                $d \leftarrow$ diameter of $h = \{t_1, t_2, t_3, t_4\}$
                if $d < d_*$ AND $d \leqslant \tau_u$ then
                    $d_* \leftarrow d$
                    Mark $\{t_1^*, t_2^*, t_3^*\} \leftarrow \{t_1, t_2, t_3\}$ to be removed and $t_4^* \leftarrow t_4$ to be added to $G$
    if $d_*$ is not $\infty$ then
        Add $t_4^*$ to $V$
        $N \leftarrow$ union of neighbors of $\{t_1^*, t_2^*, t_3^*\}$
        for $t \in N$ do
            if $t_4^*$ and $t$ have two points in common then
                Add edge $(t_4^*, t)$ to $E$
        Remove $\{t_1^*, t_2^*, t_3^*\}$ from $B$
        update ← 1
Remove disconnected tetrahedrons from $B$ by removing its components of length $\leqslant 4$
Output: Nodes in $B$
```

6 Covers and graphical contraction

We presume that a spatial embedding $\mathcal{E}$ of the data set $\mathcal{U}$ is available that maps all of its points bijectively to points in either $\mathbb{R}^2$ or $\mathbb{R}^3$ (Cartesian coordinates), $\mathcal{E} : U \to \mathbb{R}^2$ or $\mathbb{R}^3$. Without loss of generality, we will consider embedding to be in $\mathbb{R}^3$.

a. Defining and computing covers of cycles: We denote a local cover of a cycle C in the embedding of the full data set as follows. Let there be k points in a cycle C, denoted by $\{c_1, ..., c_k\} \subset U$. Its embedding is $\mathcal{E}(C) = \{\mathbf{p}_1, ..., \mathbf{p}_k\}$, where $\mathbf{p}_j = (p_j^1, p_j^2, p_j^3), 1 \leqslant j \leqslant k$. Then, the dimensions of the smallest

hyper-rectangle (in Cartesian coordinates) that contains the cycle is $\prod_{d=1}^{3}\left[\min\{p_j^d\}_{j=1}^k, \max\{p_j^d\}_{j=1}^k\right]$. We define a cover of C as the set of all points of the data set that are embedded inside or on this hyper-rectangle. In other words, $\bar{C} = \{c \in U \mid \mathcal{E}(c) \in \prod_{d=1}^{3}\left[\min\{p_j^d\}_{j=1}^k, \max\{p_j^d\}_{j=1}^k\right]\}$ is the cover of C.

b. Eliminating cycles that cannot be around significant holes: We determine whether a cycle $C_i \in \mathcal{C}$ cannot wrap around a significant topological feature. The cover of every cycle C_i is computed, denoted by $\bar{C}_i$. We compute PH up to and including τ for the embedded points $\mathcal{E}(\bar{C}_i)$. The number of significant features is denoted by $n(\bar{C}_i)$. If $n(\bar{C}_i) = 0$, then the embedding of cover $\bar{C}_i$ does not contain any significant feature. Hence, cycle C_i cannot wrap around any significant feature in $\mathcal{E}(U)$. We ignore such cycles in subsequent analysis.

c. Graphical contraction of covers: At this point, $\bar{\mathcal{C}} = \{\bar{C}_i\}$ is a collection of covers that possibly contain at least one significant feature in $\mathcal{E}(\bar{C}_i)$. We define two rules to update this collection such that there is a possible decrease in the number of covers and/or decrease in the number of points in some covers. Given an upper threshold for PH τ_u, threshold for significance ϵ, and $\bar{\mathcal{C}} = \{\bar{C}_i\}$ with $n(\bar{C}_i) > 0$, we implement the following conditions.

   1. Subset check: If $\bar{C}_j \subset \bar{C}_i$ and $n\left(\bar{C}_i\right) = n\left(\bar{C}_j\right)$, then remove $\bar{C}_i$ from $\bar{\mathcal{C}}$.
   2. Intersection check: If $\bar{C}_i \cup \bar{C}_j \notin \{\phi, \bar{C}_i, \bar{C}_j\}$ and $n\left(\bar{C}_i \cap \bar{C}_j\right) = n\left(\bar{C}_i\right)$ and/or $n\left(\bar{C}_j\right)$, then replace $\bar{C}_i$ and/or $\bar{C}_j$ by $\bar{C}_i \cap \bar{C}_j$. Note that we can impose a stronger condition that $\left|\bar{C}_i \cup \bar{C}_j\right| > 3$, because otherwise the intersection cannot have a significant feature.

   We implement this by constructing a graph with nodes as the elements of $\bar{\mathcal{C}}$ (Algorithm 5).

If a spatial embedding is not available, then we can implement multiple permutations and select a set of minimal boundaries as defined above.

7 Perturbations and permutations

The rationale for perturbations and permutations possibly yielding shorter representatives is as follows.

a. Perturbations: Consider a persistence pair (b, d). The algorithm will find a representative boundary around this feature, say C^1, that is born at b, i.e. the length of longest edge in C^1 is b. Suppose that there exists a cycle C^2 in the embedding that is born at $b + \Delta$ $(\Delta > 0)$ and is closer to the hole as compared to C^1. We are interested in discovering C^2 if Δ is sufficiently small $(b + \Delta < \tau_u)$. By perturbing the data set, we allow the possibility that the longest edge in C^2 is smaller than the longest edge in C^1 in the perturbed data set, and hence, the former can be discovered by the algorithm as a representative boundary.

b. Permutations: The indices of the edges that are added to the simplicial complex at the same spatial scale, are permuted. The resulting change in the order of columns of boundary matrices, can give a different set of representative boundaries. Note that, unlike perturbations which are essentially relying on the robustness of meaningful topological features , a permutation preserves the PD.

We add stochasticity in the two different ways in each $\bar{C}_i$.

i. Spatial perturbation of points in the embedding: For every cover $\bar{C}_i$, we construct a user-defined number of perturbations, denoted by $\mathrm{n_{pert}}$. Every point p_j in $\mathcal{E}(\bar{C}_i)$ is perturbed randomly in a ball centered at the point. The radius of this ball is defined as the smaller of (distance of nearest neighbor of p_j)/3 and a maximum perturbation Δ_i that is allowed for all points in $\mathcal{E}(\bar{C}_i)$. Such an upper threshold on the magnitude of a perturbation is required so that the topology does not change significantly as compared to the unperturbed embedding. We call Δ_i the perturbation parameter for $\bar{C}_i$. It is computed as $\Delta_i = \dfrac{\epsilon/3}{2^m}$, where m is the smallest positive integer such that the number of significant features in all the $\mathrm{n_{pert}}$ perturbations is the same as that in $\mathcal{E}(\bar{C}_i)$ (PH computed up to $\tau = \tau_u + \epsilon$).

Algorithm 5 Graphical contraction

Input: $\tau_u, \epsilon, \bar{\mathcal{C}} = \{\bar{C}_i\}$ where $n(\bar{C}_i) > 0$
Set of vertices $V \leftarrow \bar{\mathcal{C}}$
Remove C_i from V if there exists $n(\bar{C}_i) = n(\bar{C}_j)$ AND $\bar{C}_i \subset \bar{C}_j$ ▷ Subset check
Define edge set $E \leftarrow \{(\bar{C}_i, \bar{C}_j)\}$ if $\bar{C}_i \cap \bar{C}_j \notin \{\phi, \bar{C}_i, \bar{C}_j\}$
Intersection graph $G \leftarrow (V, E)$
for component c in components of G **do**
    $G_c \leftarrow$ subgraph G defined by nodes in c
    update $\leftarrow 1$
    while update **do**
        update $\leftarrow 0$
        for (c_i, c_j) in pairs of nodes of G_c **do** ▷ Intersection check
            $c_k \leftarrow c_i \cap c_j$
            Continue/Skip if $c_k \in \{c_i, c_j\}$ AND $|c_k| < 4$
            Compute $n(c_k)$ by computing PD up to $\tau_u + \epsilon$
            if $n(c_k) = n(c_i)$ **then**
                Remove c_i from G_c
            if $n(c_k) = n(c_j)$ **then**
                Remove c_j from G_c
            if c_i and/or c_j removed **then** ▷ Add c_k to G_c
                for c_i in G_c **do**
                    If $c_k \subset c_i$ AND $n(c_k) = n(c_i)$, remove c_i ▷ Subset check
                Update edge set E to add neighbors of c_k
            update $\leftarrow 1$
            break
$G \leftarrow$ composition of all G_c
Output: List of nodes of G

ii. Permutations for every perturbation: We permute the indices of edges that have the same diameter. This results in permutation of columns of the coboundary and the boundary matrices. This stochasticity is merely a change in labels and therefore introduces nothing topologically new into the computation, but helps to overcome the greedy algorithm's local minimum problem. We construct $\mathrm{n_{perm}}$ number of permutations, a user-defined hyperparameter. It is possible that some re-indexed sets result in the exact same indexing of edges. There is a higher chance of this if the number of maximum possible unique indexing is not very large as compared to $\mathrm{n_{perm}}$. Therefore, we discard a permutation if its indexing of edges already exists in the set of permutations.

iii. Processing permutations of perturbations to construct the final set of minimal representatives: We compute representative homology boundaries for the valid permutations of all perturbations of all covers. We then determine minimal representative boundaries from the multiple computed sets. One way can be to compare lengths of the multiple computed boundaries around a topological feature. To do so, first a matching of features across different PDs is required. There are methods to match persistence pairs between two PDs, for example, minimizing bottleneck distance and minimizing Wasserstein distance, to name a few. However, no metric can ensure that two features being matched across PDs will correspond to a significant hole located in a similar region in the embedding. PDs across permutations of the same perturbation will have the same set of persistence pairs, but even then there can be ambiguity if there exist two significant features with the same birth and death.

    Therefore, we do not match persistence pairs and define the set of minimal representative boundaries as follows. We first disregard all representative homology boundaries that do not contain significant features. Then, we pick all sets that minimize the longer of the remaining boundaries as follows. We sort boundaries in each set by decreasing order of length. The number of boundaries is equalized across all sets by inserting boundaries of length 0. Then the list of sets of representative boundaries is sorted

in increasing order of the length of longest boundary as first priority, second longest length as second priority, and so on. We select the sets of representative boundaries with the lowest order in the resulting sorted list. If only one set has the minimum order, then that set of representative boundaries is defined as the minimal set and we are done. If there are multiple sets with minimum order, then we define the union of all simplices in the representatives in these sets as the set of minimal representatives.

References

1. Bauer U. Ripser: efficient computation of Vietoris–Rips persistence barcodes. Journal of Applied and Computational Topology. 2021; p. 1–33.

2. Aggarwal M, Periwal V. Dory: Overcoming Barriers to Computing Persistent Homology. arXiv preprint arXiv:210305608. 2021;.
