# Supplementary material for: Tight basis cycle representatives for persistent homology of large biological data sets
Source: PLoS Comput Biol. 2023 May 30;19(5):e1010341. doi: 10.1371/journal.pcbi.1010341 (PMC10275456; doi:10.1371/journal.pcbi.1010341)
Supplement: S1 Table — Data sets of the case-study comparing Hi-C experiments with different protocols. Parameters for PH computation after ICE normalization and rescaling—PH thresholds (τi) and birth-thresholds (τu,i) to compute tight representatives. (PDF) [file pcbi.1010341.s003.pdf]

Exp.	Cross-linking	Restriction enzyme	4DN accession	$(\tau_{u,1}, \tau_1)$	$(\tau_{u,2}, \tau_2)$	$(\tau_{u,3}, \tau_3)$
1	FA	DpnII	4DNEXRAEERUF	(14.46, 32.47)	(22.83, 32.47)	(32.46, 42.88)
2	FA+DSG	DdeI	4DNEX2SUQP87	(14.21, 34.56)	(23.79, 34.56)	(32.55, 45.66)
3	FA+DSG	DpnII+DdeI	4DNEXSFVDRQD	(13.63, 32.99)	(23.07, 32.99)	(32.98, 43.52)
4	FA+DSG	DpnII	4DNEXQBYXQKH	(13.57, 32.63)	(22.69, 32.63)	(32.63, 43.28)
5	FA+DSG	Mnase	4DNESWST3UBH	(14.5, 31.93)	(23.27, 31.93)	(31.92, 40.53)
