# Supplementary figures and images for: Tight basis cycle representatives for persistent homology of large biological data sets
Source: PLoS Comput Biol. 2023 May 30;19(5):e1010341. doi: 10.1371/journal.pcbi.1010341 (PMC10275456; doi:10.1371/journal.pcbi.1010341)

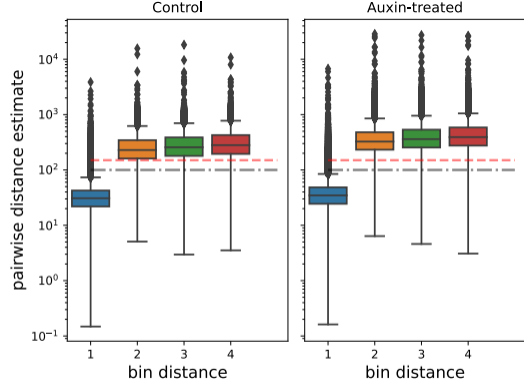

(A)

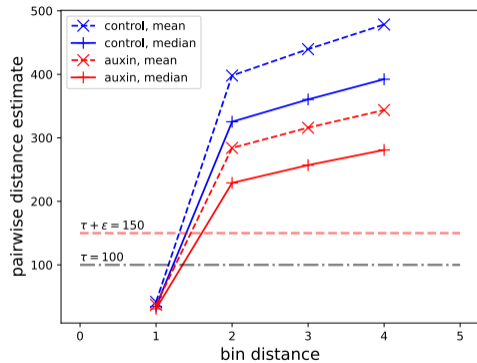

(B)

Supplement: S1 Fig — (A) Distribution of pairwise estimates for a bin distance of 1 is significantly lower as compared to those of higher bin distances. (B) Means and medians of the distributions increase with an increase in bin distances. (PDF) [file pcbi.1010341.s004.pdf]

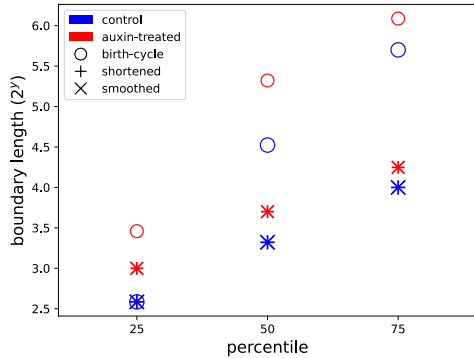

(A)

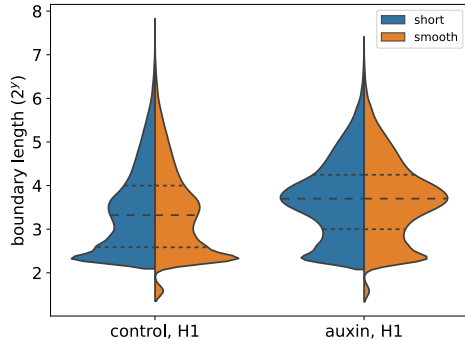

(B)

Supplement: S2 Fig — (A) Interquartile lengths of the set of representative boundaries decreased by multiple log scales using the greedy shortening algorithm. (B) Local smoothing reduces some boundaries to degenerate cycles of length two as is shown by the lowermost humps in the distribution of smooth cycles. (PDF) [file pcbi.1010341.s005.pdf]

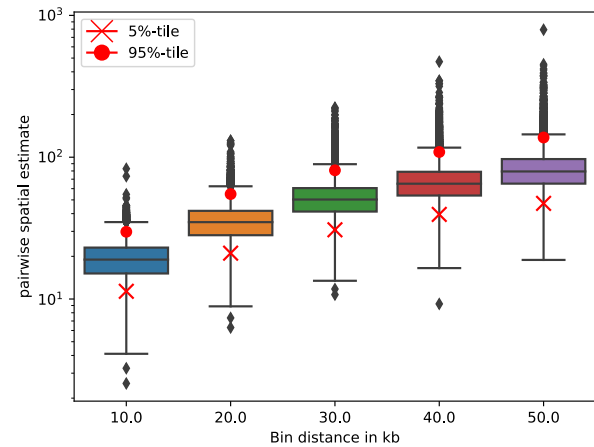

(A)

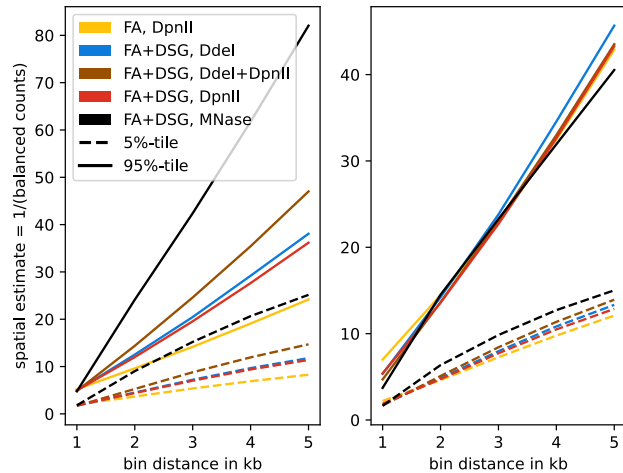

(B)

Supplement: S3 Fig — (A) Distributions of spatial estimates for different genomic distances for experiment 1. (B) 5%-tile and 95%-tile of distribution of spatial estimates at different bin distances. Left panel is before scaling and right panel is after scaling. (PDF) [file pcbi.1010341.s006.pdf]

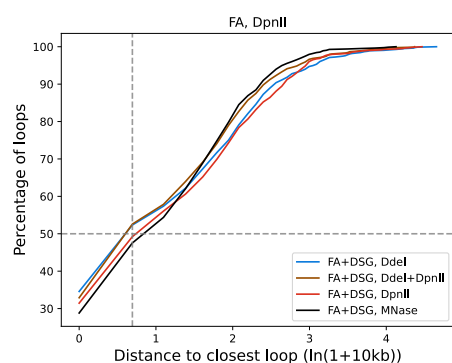

(A)

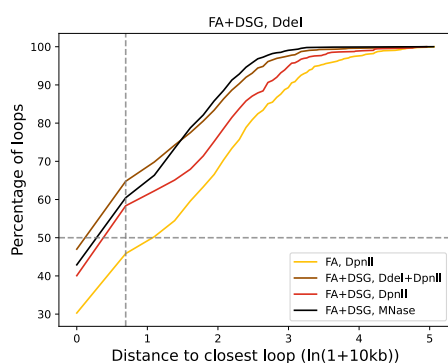

(B)

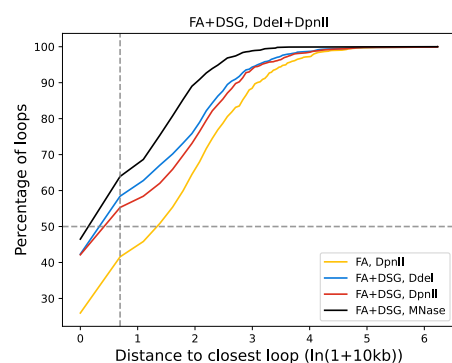

(C)

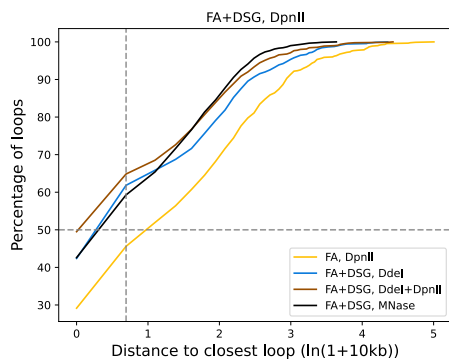

(D)

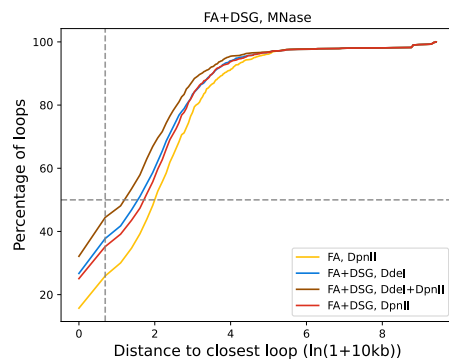

(E)

Supplement: S4 Fig — Distances of loops of experiment i from loops of experiment j were computed for 1 ≤ i, j ≤ 5. Vertical dashed line shows threshold of 1 bin-distance between loops. Plots (A) to (E) show cumulative percentages of loop-distances computed for experiments 1 to 5 with every other experiment. (PDF) [file pcbi.1010341.s007.pdf]

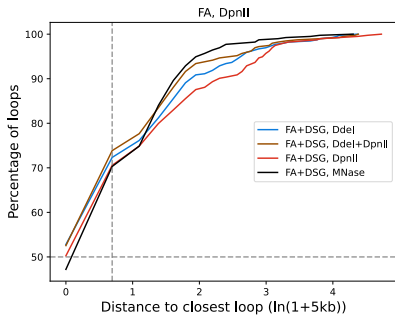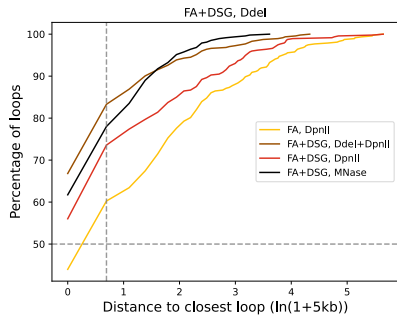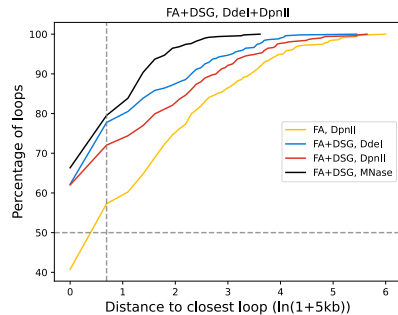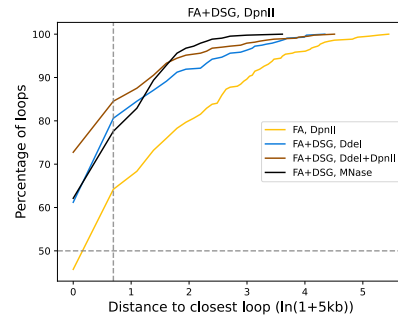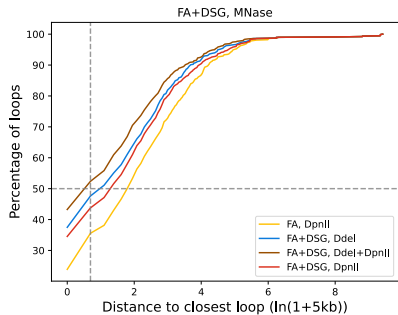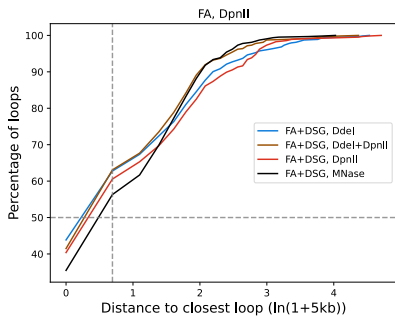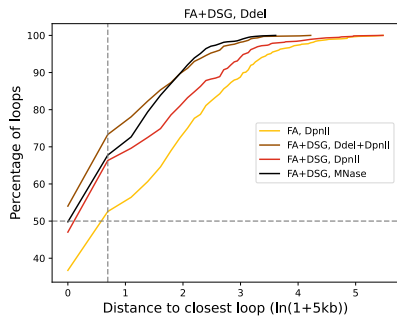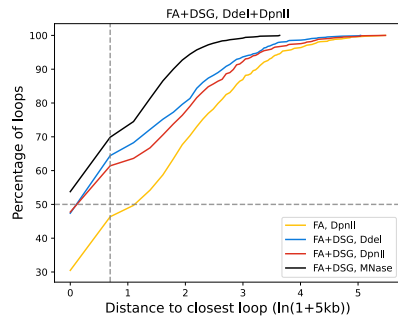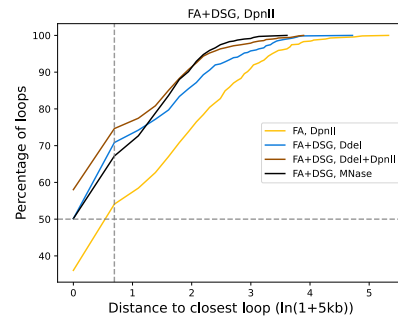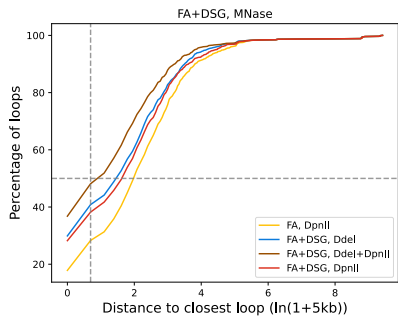

Supplement: S5 Fig — Consistency of computed tight H1 loops across different experiments 1 to 5 (left to right) at (τ1, τu,1) (top row) and (τ2, τu,2) (bottom row). (PDF) [file pcbi.1010341.s008.pdf]

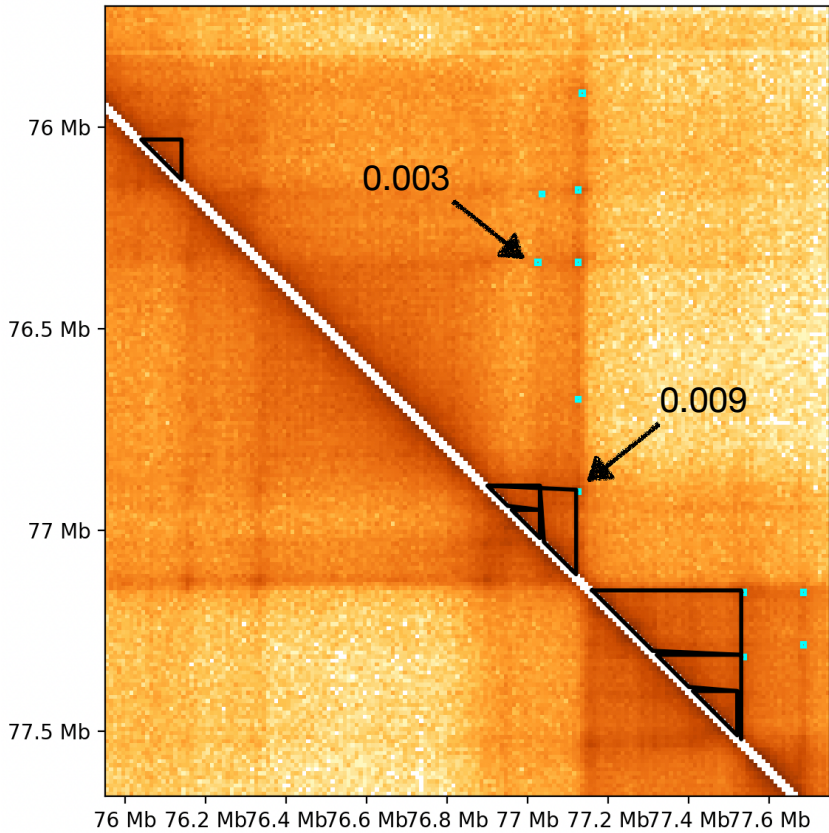

Supplement: S6 Fig — HiCCUPS peaks (teal dots) that do not match a H1 loop (black lines) have a lower balanced Hi-C frequency as compared to those that match. (PDF) [file pcbi.1010341.s009.pdf]

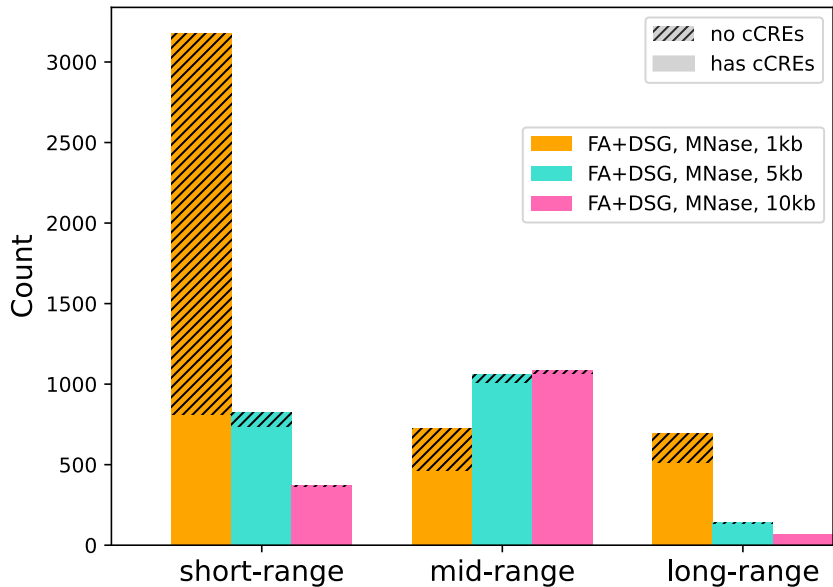

Supplement: S7 Fig — The majority of loops in all but one category contain cCREs. (PDF) [file pcbi.1010341.s010.pdf]

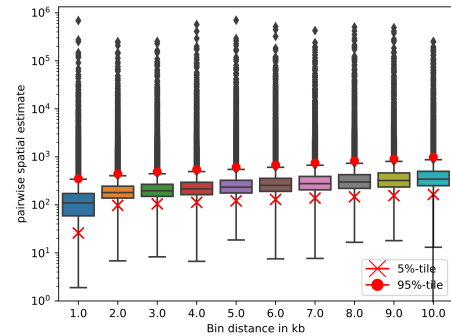

(A)

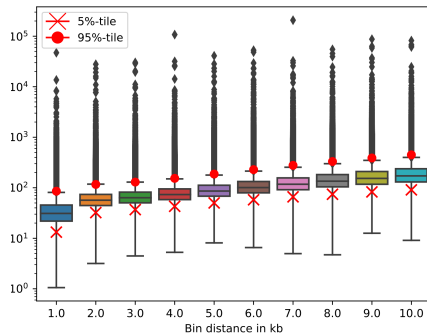

(B)

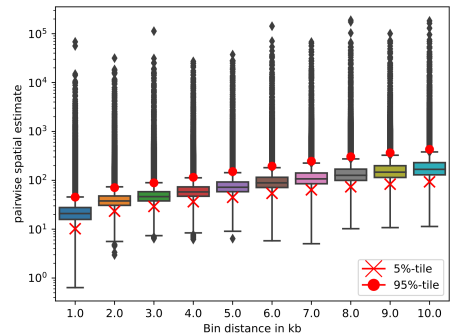

(C)

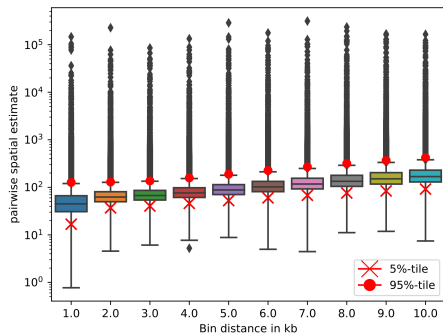

(D)

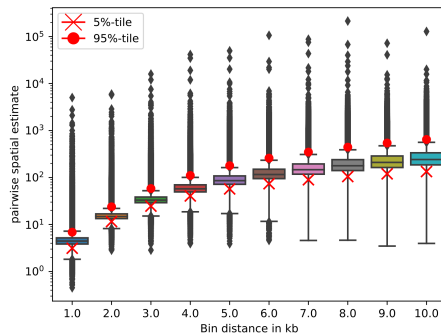

(E)

Supplement: S8 Fig — Distributions of spatial estimates at different bin-distances for experiments 1 to 5 (panel A to E). Distributions for MNase (panel E) are more distinct at lower bin-distances as compared to other experiments. (PDF) [file pcbi.1010341.s011.pdf]

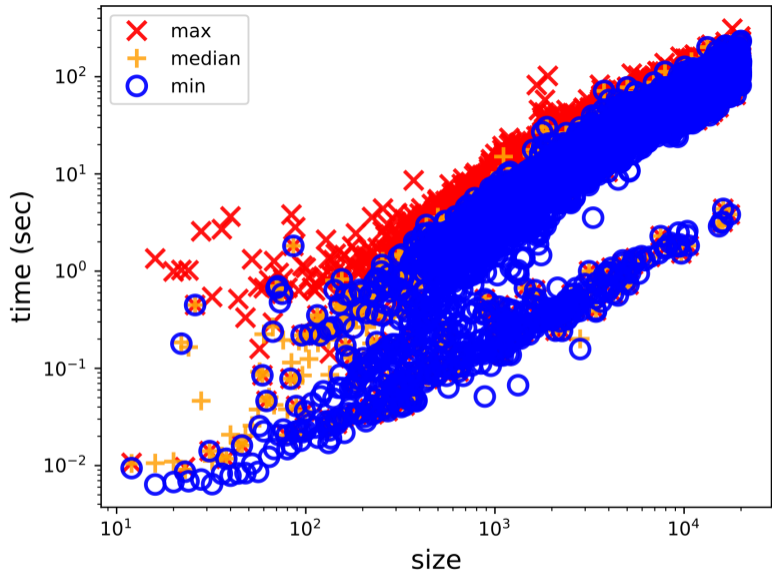

(A)

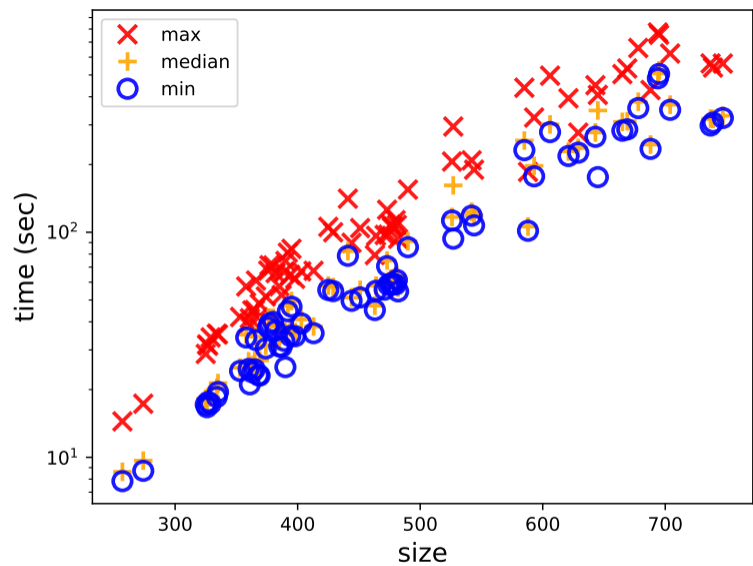

(B)

Supplement: S9 Fig — (A) PD computation times of 174, 574 PDB entries up to threshold of 13.5 Å. (B) PD and tight representation computation times for all significant voids identified in 25 homolog sets. (PDF) [file pcbi.1010341.s012.pdf]

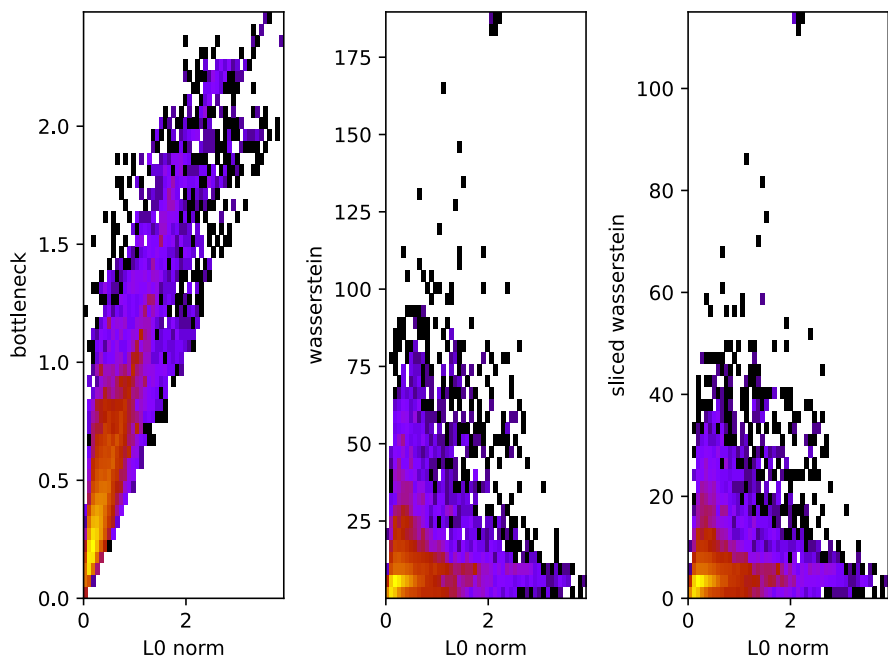

(A)

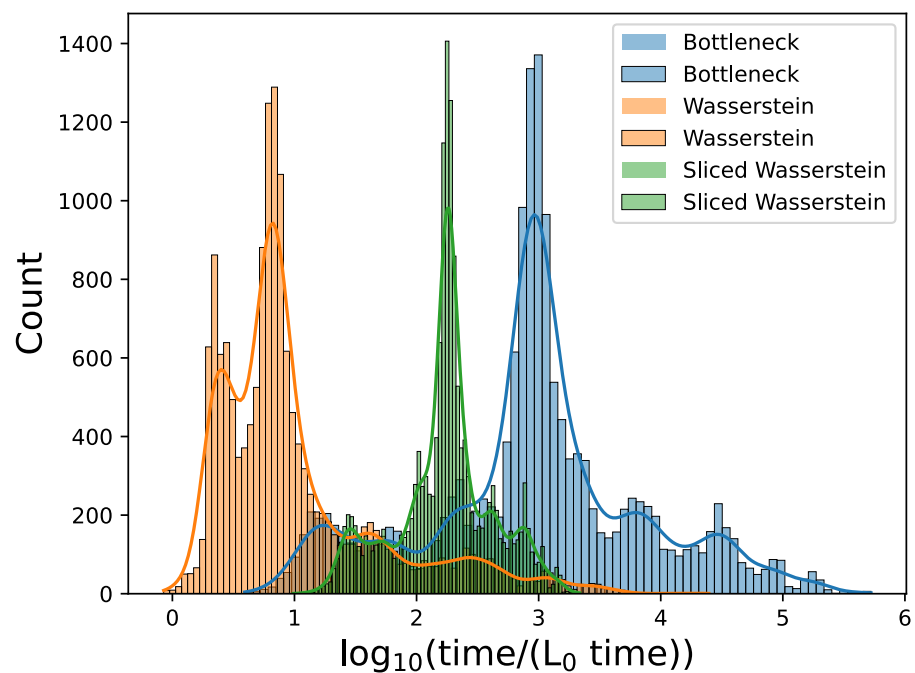

(B)

Supplement: S10 Fig — (A) Comparing different metrics for H2 PDs of pairwise homologs. L0 norm agrees with the bottleneck distance. (B) Comparing computation times with respect to L0 norm computation. L0 metric agrees with Bottleneck distance and is computationally faster by multiple orders or magnitude. (PDF) [file pcbi.1010341.s013.pdf]

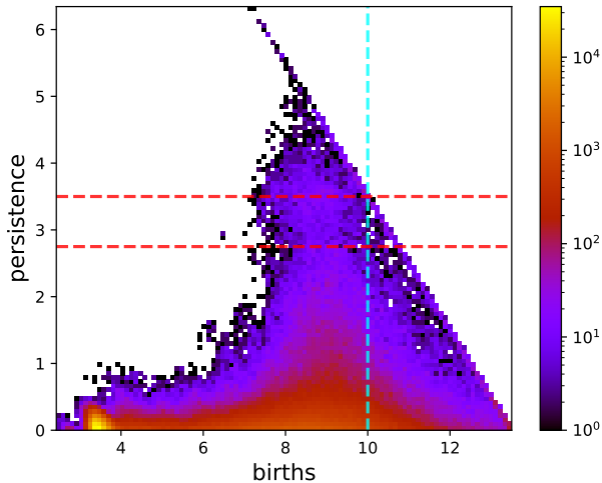

Supplement: S11 Fig — A cluster of features with relatively high persistence exists between 7 ≤ birth ≤ 10. These features have persistence at least 2.75, shown by the lower dashed red line. Our choice of ϵ = 3.5 is shown by the upper dashed red line. (PDF) [file pcbi.1010341.s014.pdf]

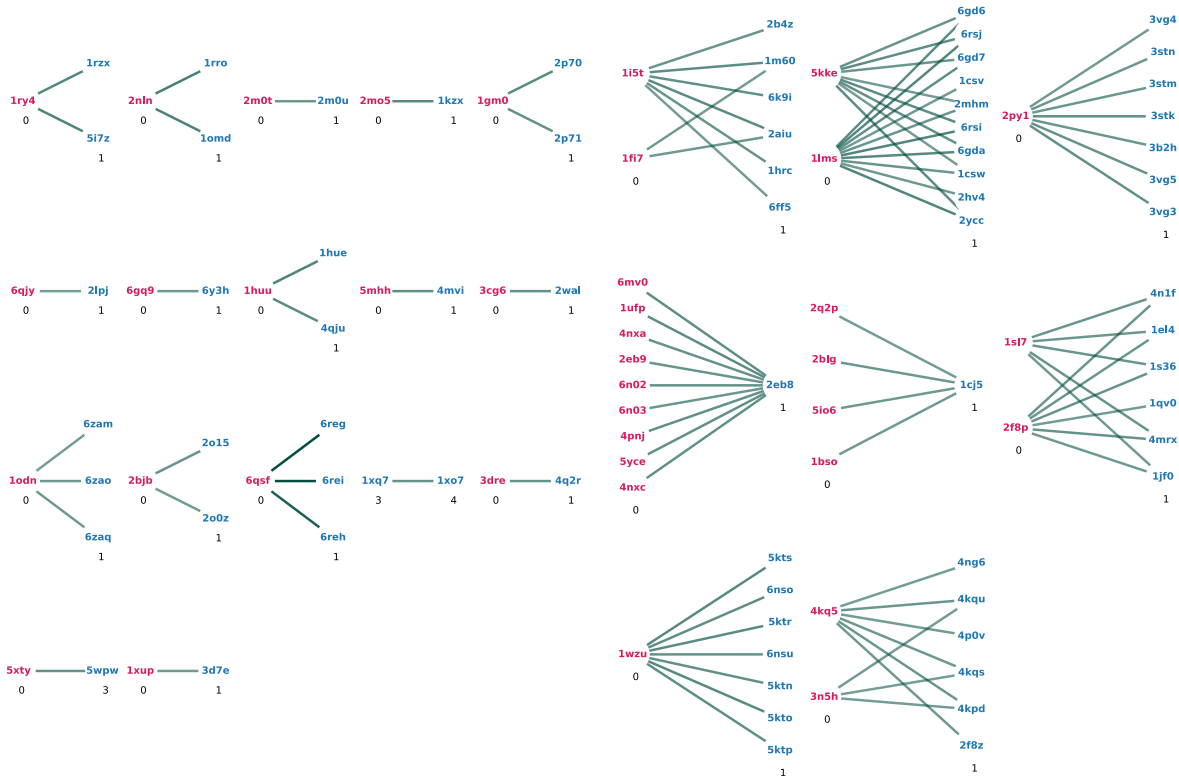

(A)

(B)

Supplement: S12 Fig — Black labels are the number of significant H2 features in every PDB in that column. (PDF) [file pcbi.1010341.s015.pdf]
