# Supplementary material for: Tight basis cycle representatives for persistent homology of large biological data sets
Source: PLoS Comput Biol. 2023 May 30;19(5):e1010341. doi: 10.1371/journal.pcbi.1010341 (PMC10275456; doi:10.1371/journal.pcbi.1010341)
Supplement: S13 Fig — (PDF) [file pcbi.1010341.s016.pdf]

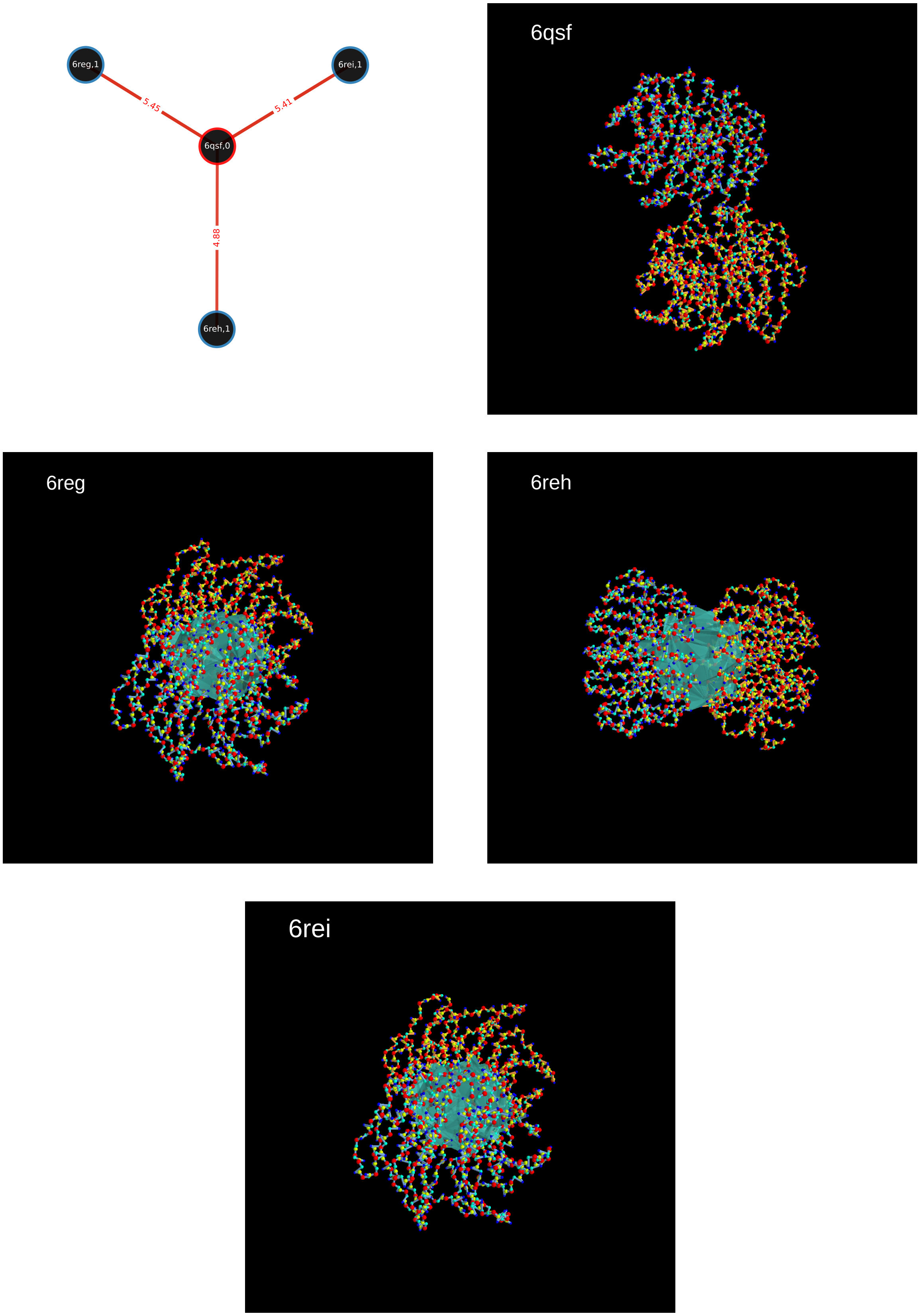

Figure 1

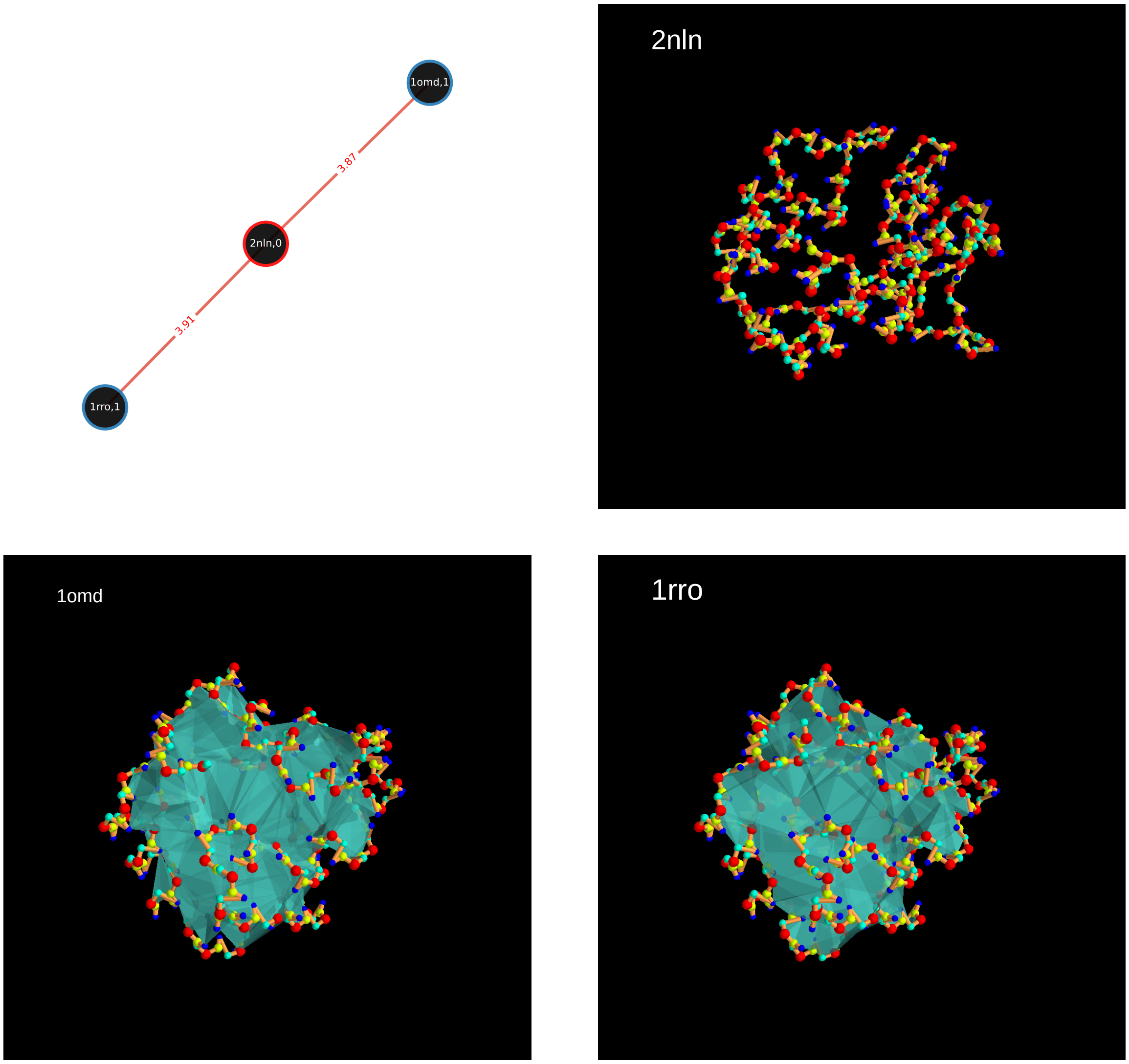

Figure 2

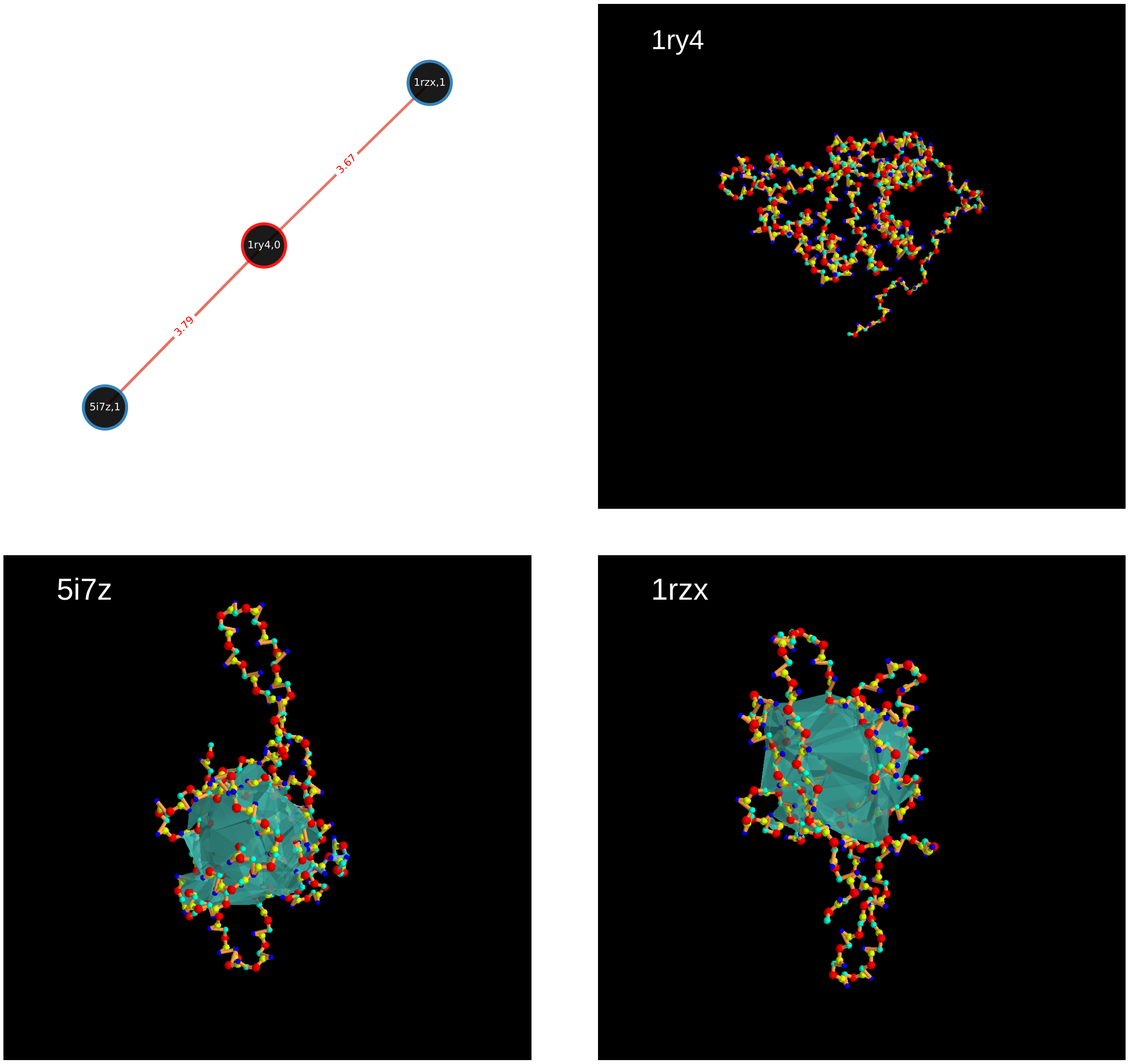

Figure 3

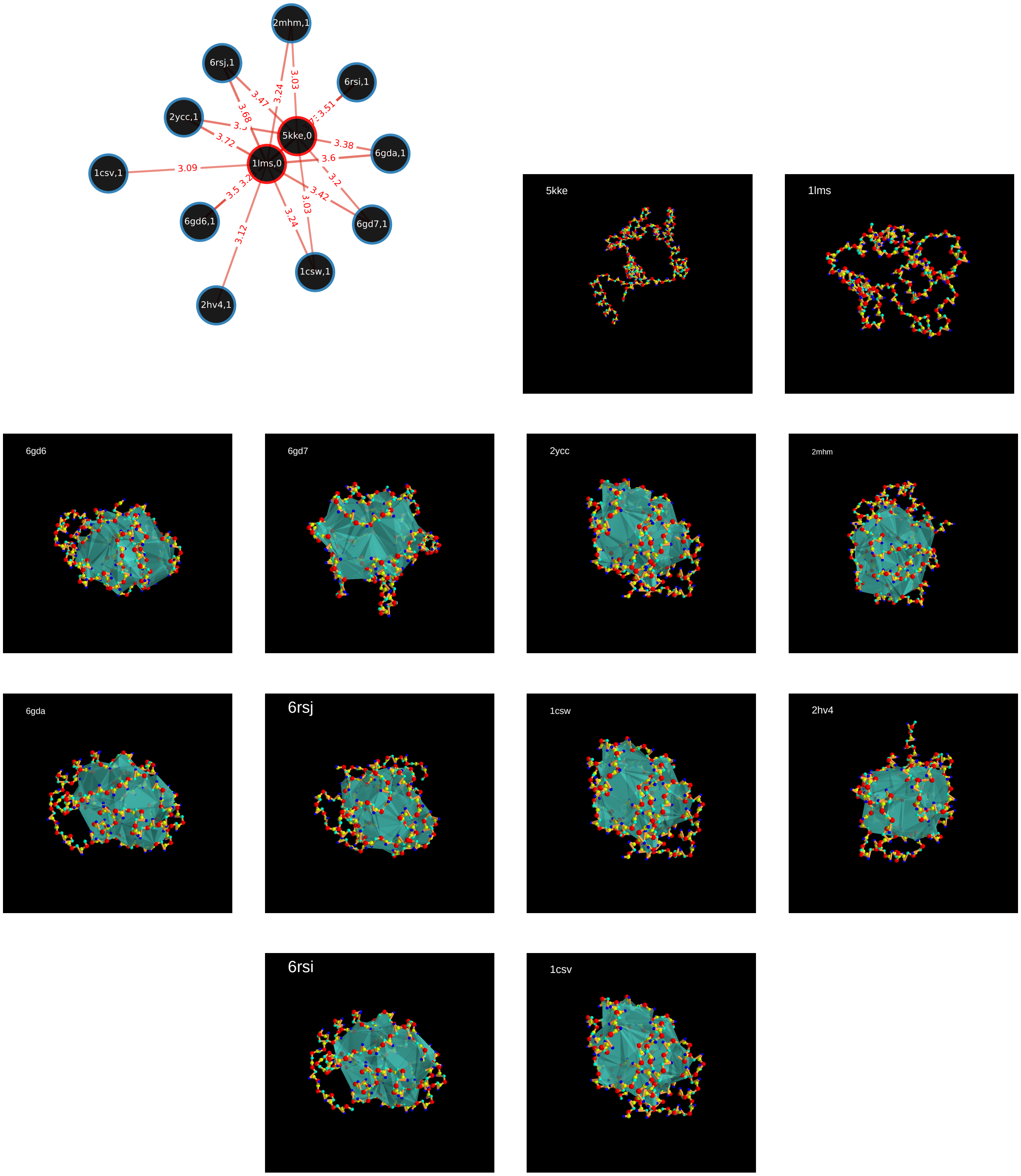
2mhm,1
6rsj,1
6rsi,1
2ycc,1
5kke,0
6gda,1
1csv,1
1lms,0
6gd6,1
6gd7,1
1csw,1
2hv4,1
3.03
3.24
3.47
3.68
3.51
3.72
3.38
3.6
3.09
3.5
3.2
3.42
3.03
3.24
3.12
5kke
1lms
6gd6
6gd7
2ycc
2mhm
6gda
6rsj
1csw
2hv4
6rsi
1csv

Figure 4

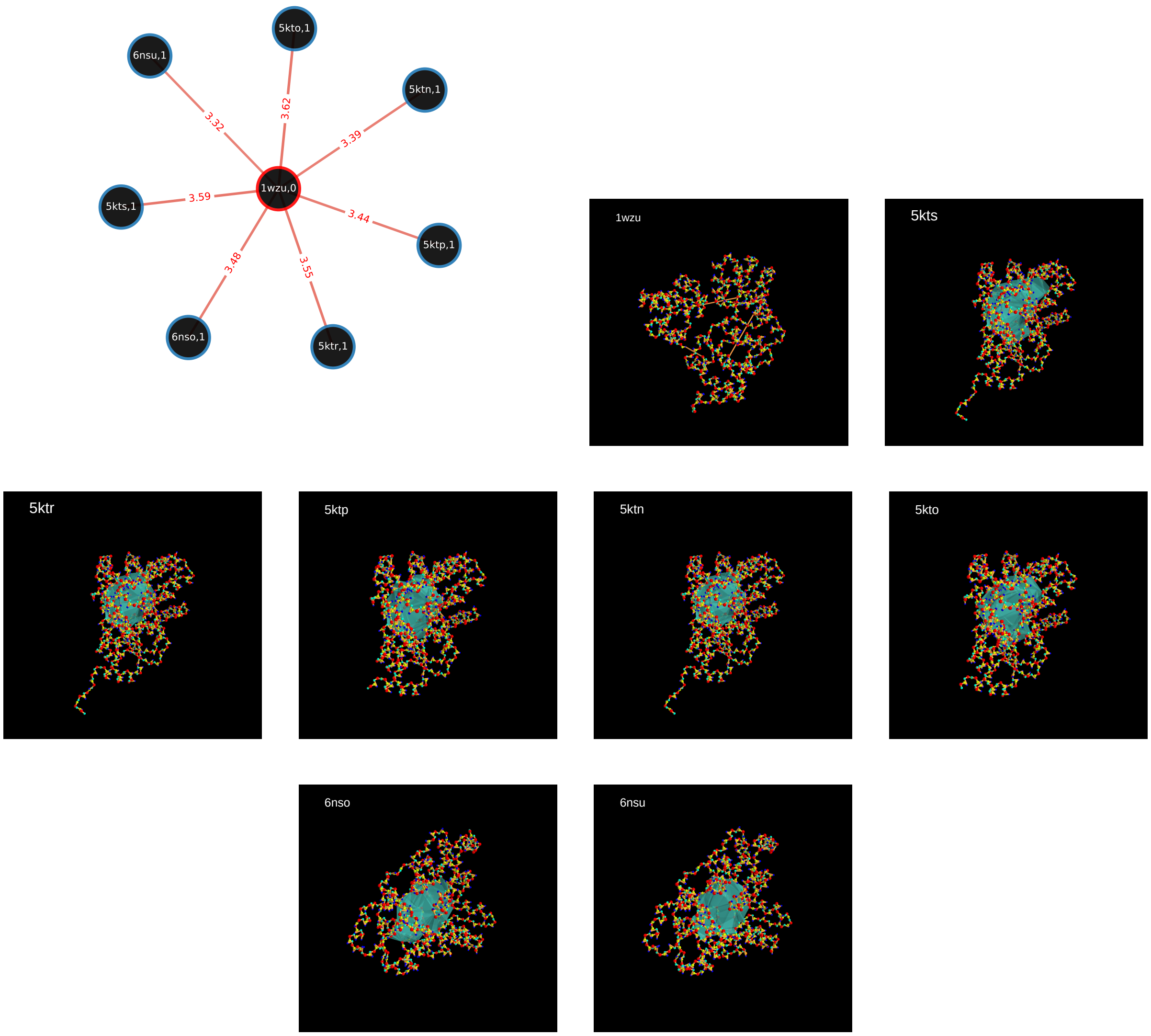
6nsu,1
5kto,1
5ktn,1
5kts,1
1wzu,0
5ktp,1
6nso,1
5ktr,1
3.32
3.62
3.39
3.59
3.44
3.48
3.55
1wzu
5kts
5ktr
5ktp
5ktn
5kto
6nso
6nsu

Figure 5

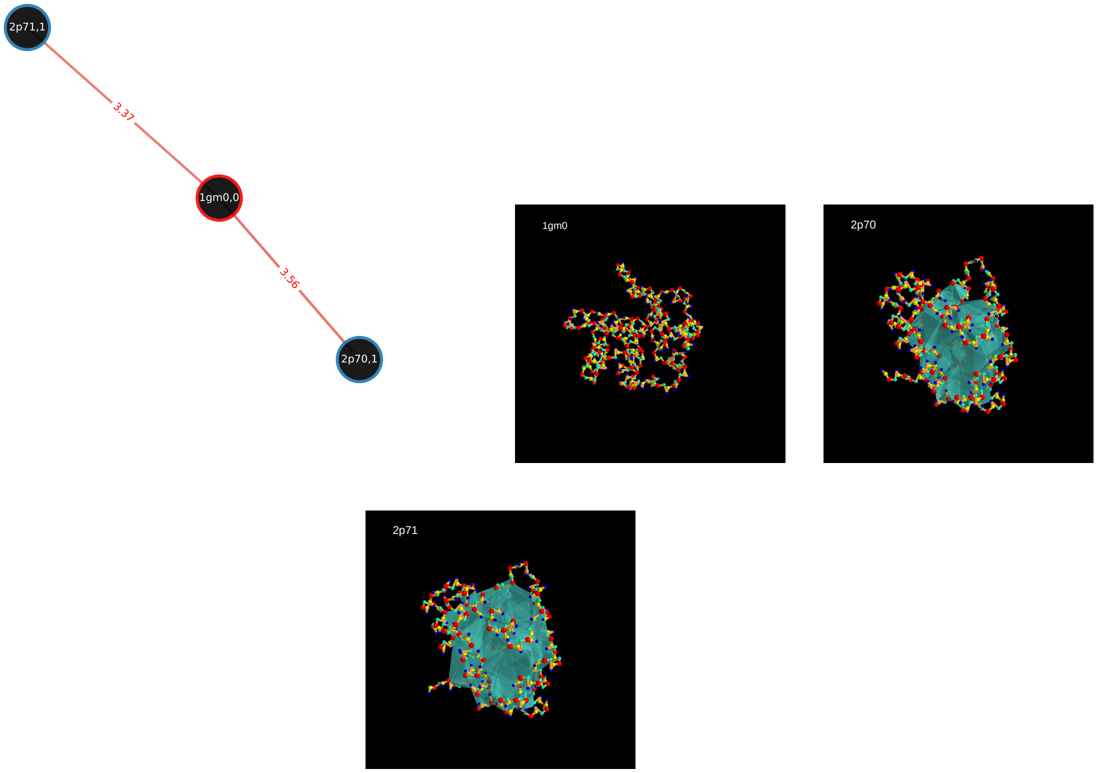

Figure 6

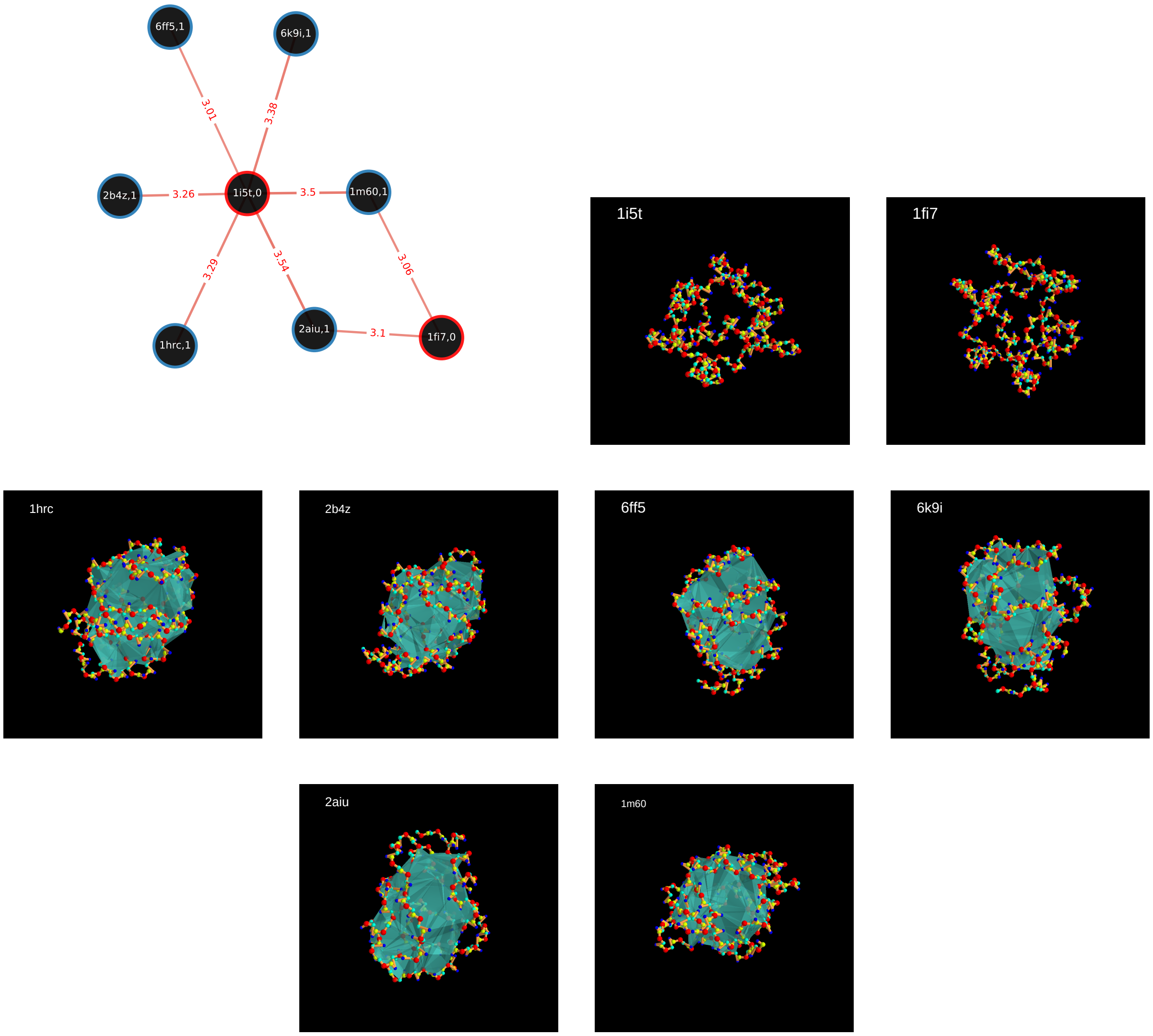
6ff5,1
6k9i,1
2b4z,1
1i5t,0
1m60,1
1hrc,1
2aiu,1
1fi7,0
3.01
3.38
3.26
3.5
3.06
3.29
3.54
3.1
1i5t
1fi7
1hrc
2b4z
6ff5
6k9i
2aiu
1m60

Figure 7

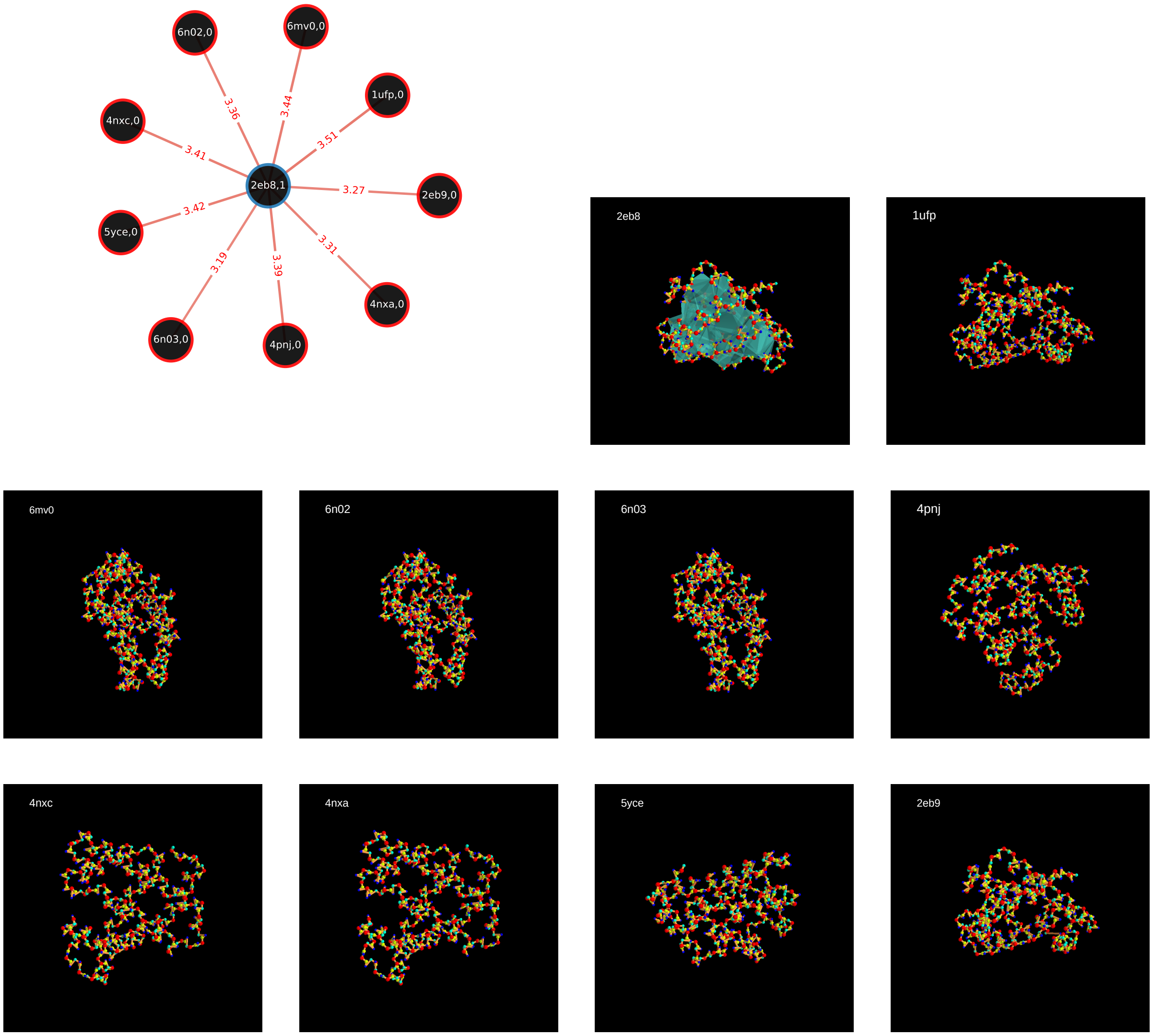
6n02,0
6mv0,0
1ufp,0
4nxc,0
2eb8,1
2eb9,0
5yce,0
4nxa,0
6n03,0
4pnj,0
3.36
3.44
3.51
3.41
3.27
3.42
3.31
3.19
3.39
2eb8
1ufp
6mv0
6n02
6n03
4pnj
4nxc
4nxa
5yce
2eb9

Figure 8

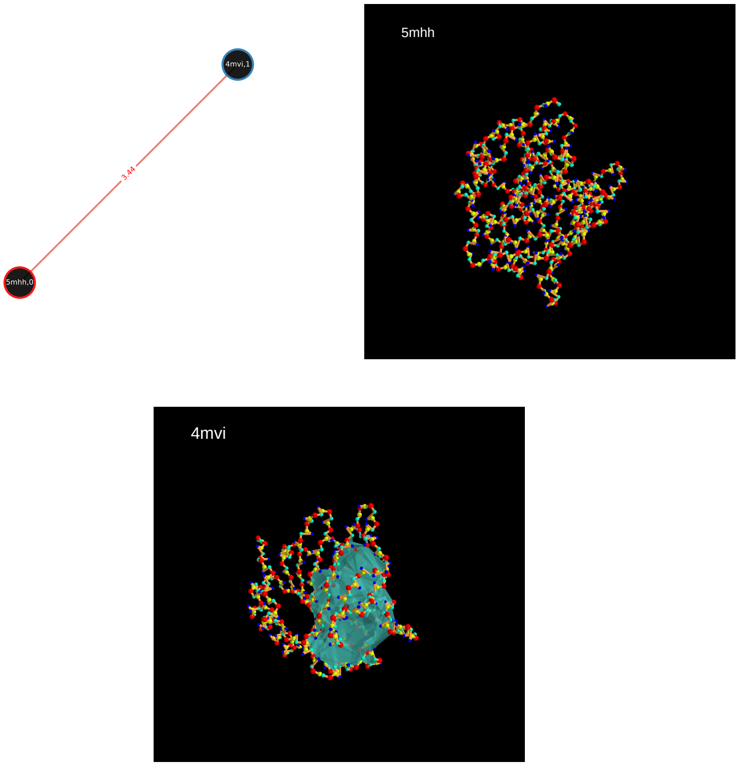

Figure 9

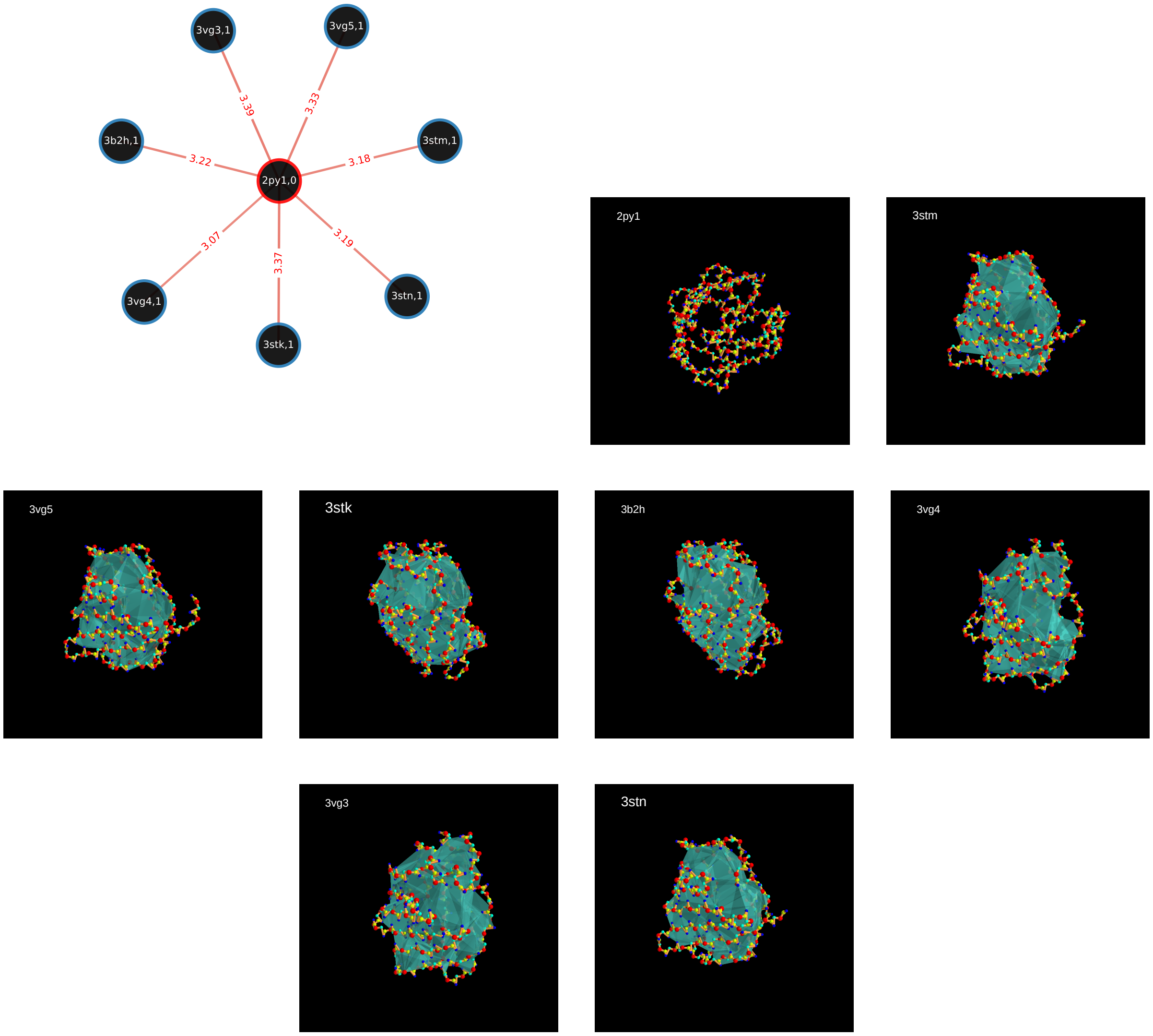
3vg3,1
3vg5,1
3b2h,1
3stm,1
2py1,0
3vg4,1
3stk,1
3stn,1
3.39
3.33
3.22
3.18
3.07
3.37
3.19
2py1
3stm
3vg5
3stk
3b2h
3vg4
3vg3
3stn

Figure 10

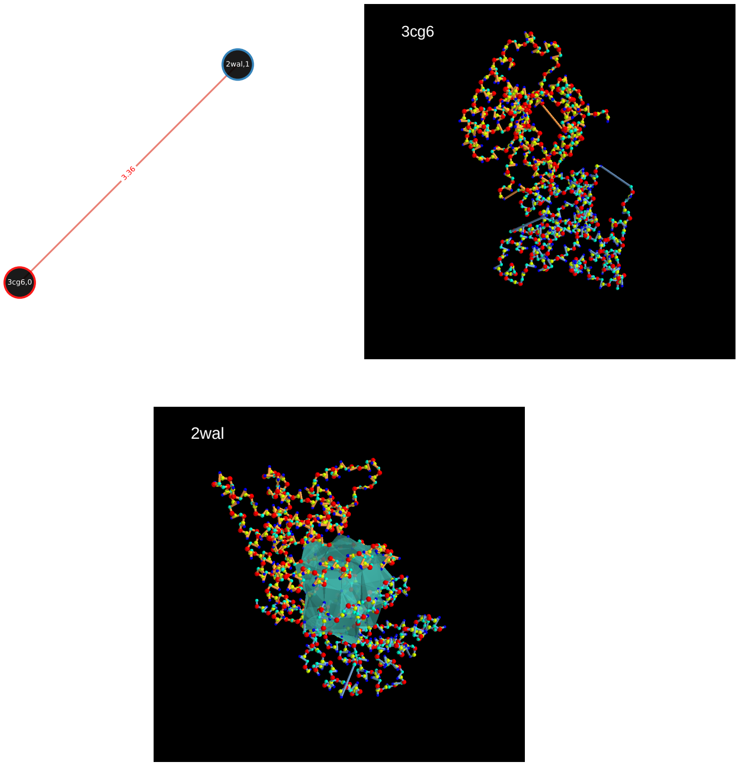

Figure 11

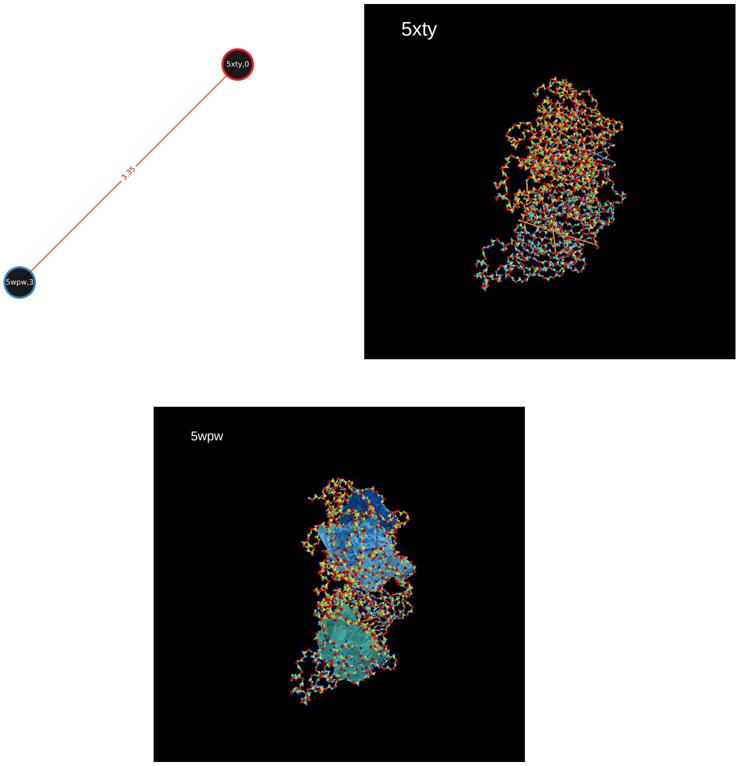

Figure 12

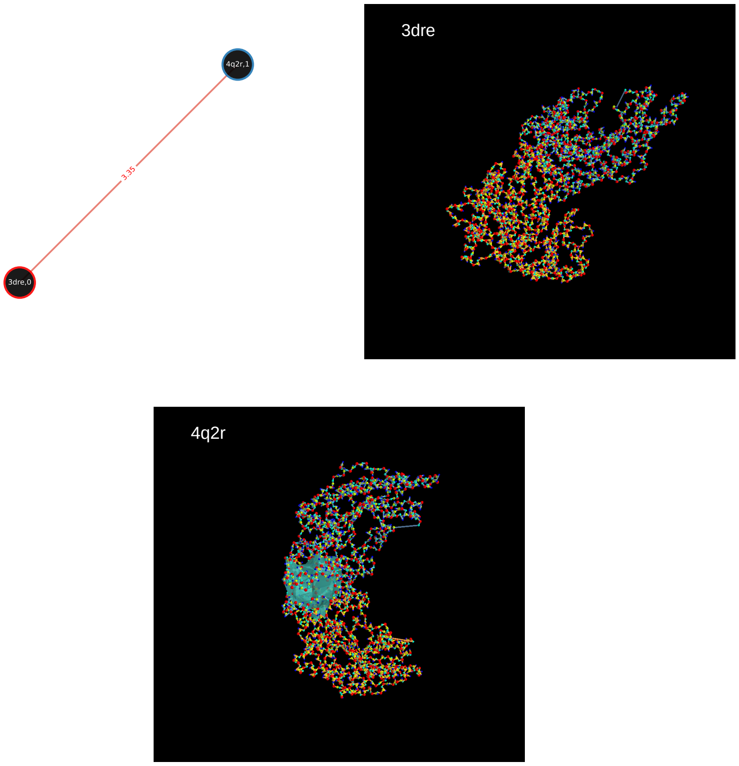

Figure 13

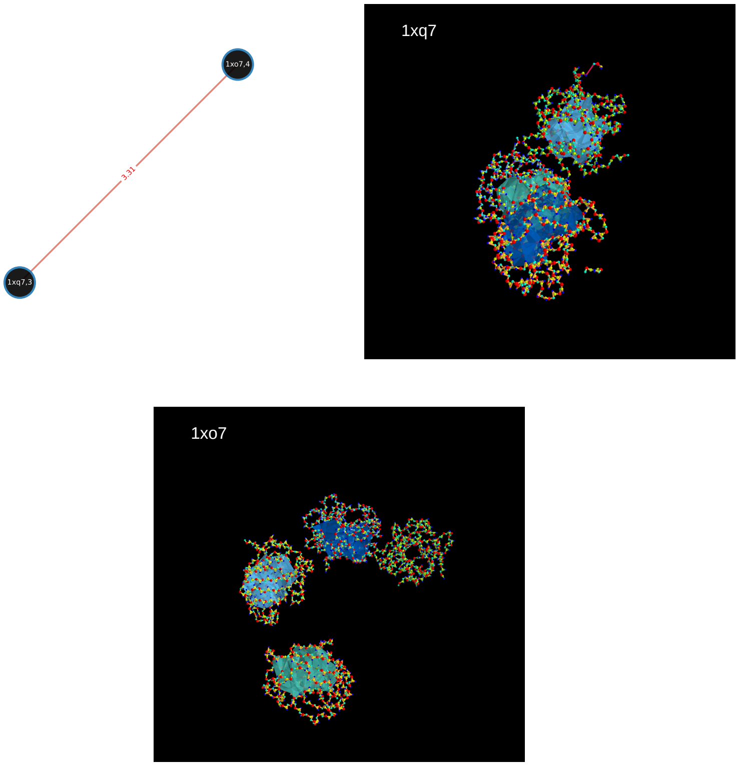

Figure 14

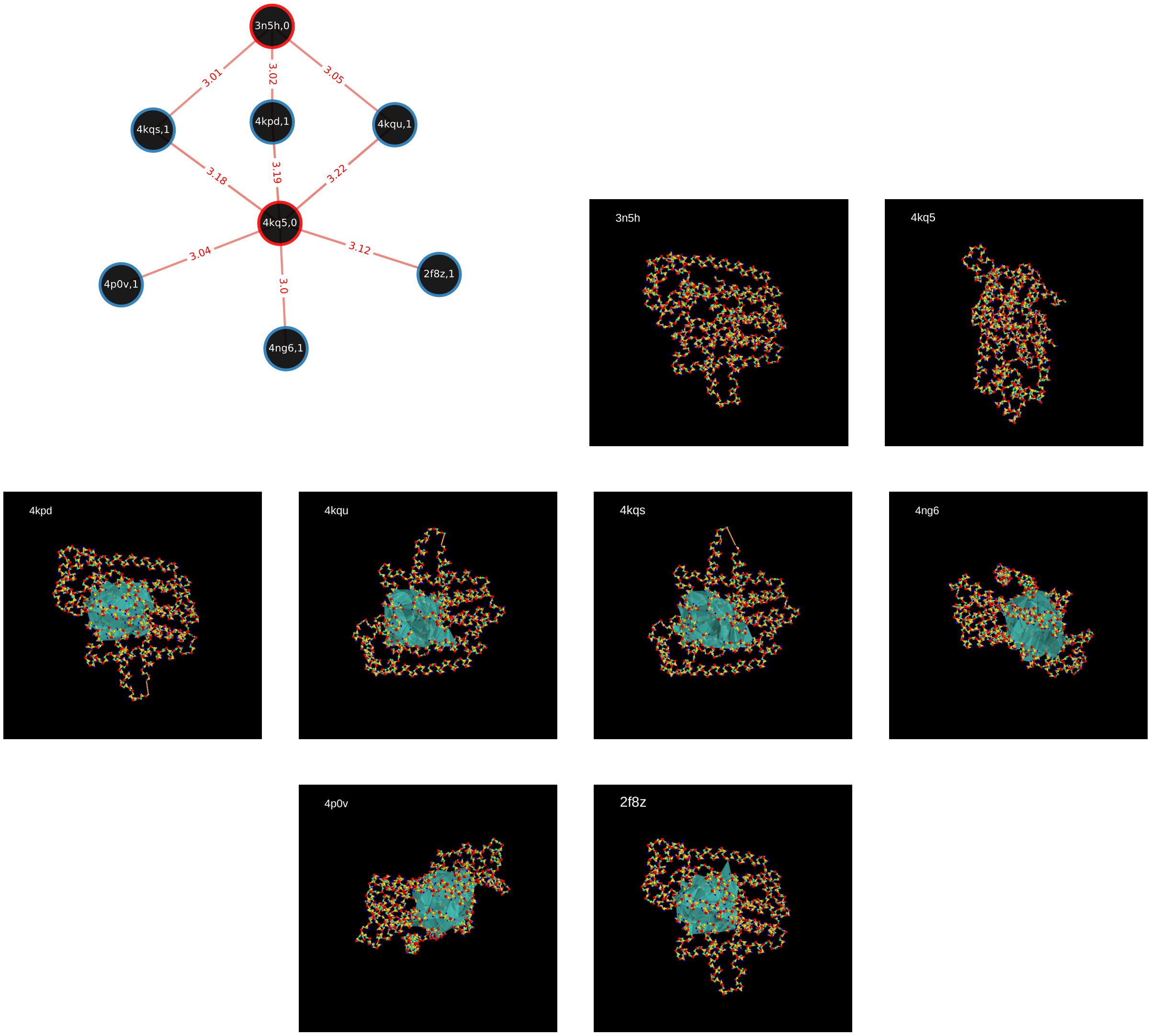
3n5h,0
3.01
3.02
3.05
4kqs,1
4kpd,1
4kqu,1
3.18
3.19
3.22
4kq5,0
3.04
3.12
3.0
4p0v,1
2f8z,1
4ng6,1
3n5h
4kq5
4kpd
4kqu
4kqs
4ng6
4p0v
2f8z

Figure 15

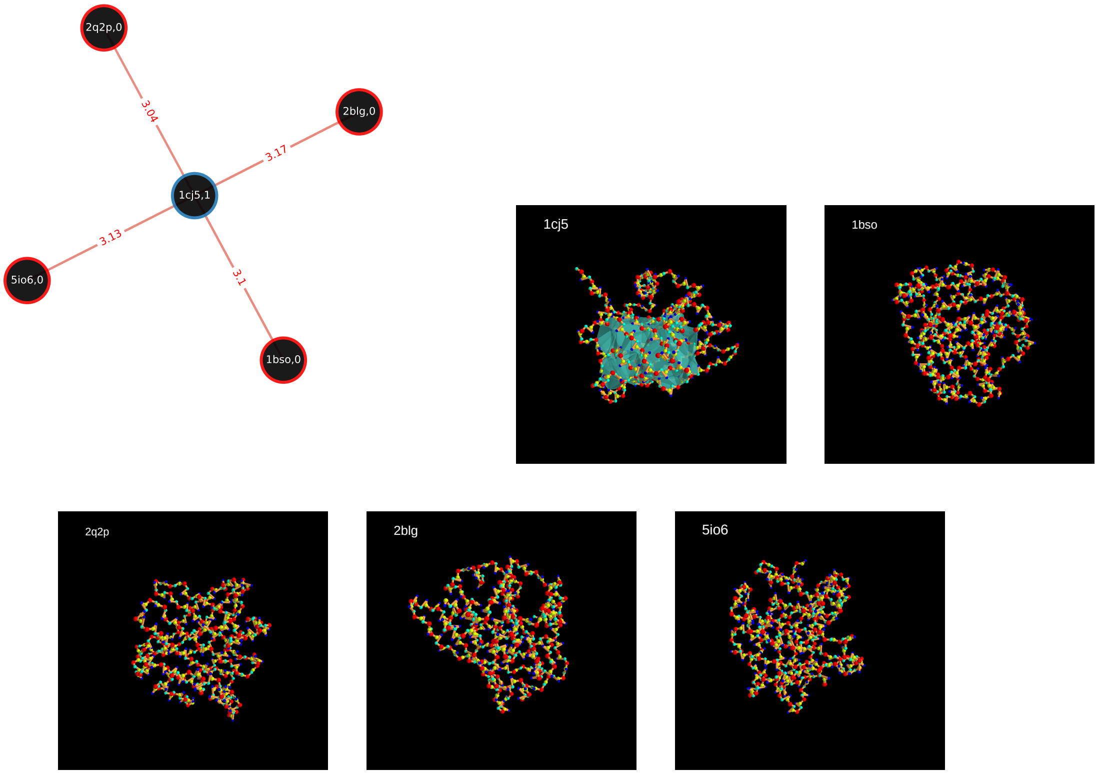
2q2p,0
3.04
2blg,0
3.17
1cj5,1
3.13
5io6,0
3.1
1bso,0
1cj5
1bso
2q2p
2blg
5io6

Figure 16

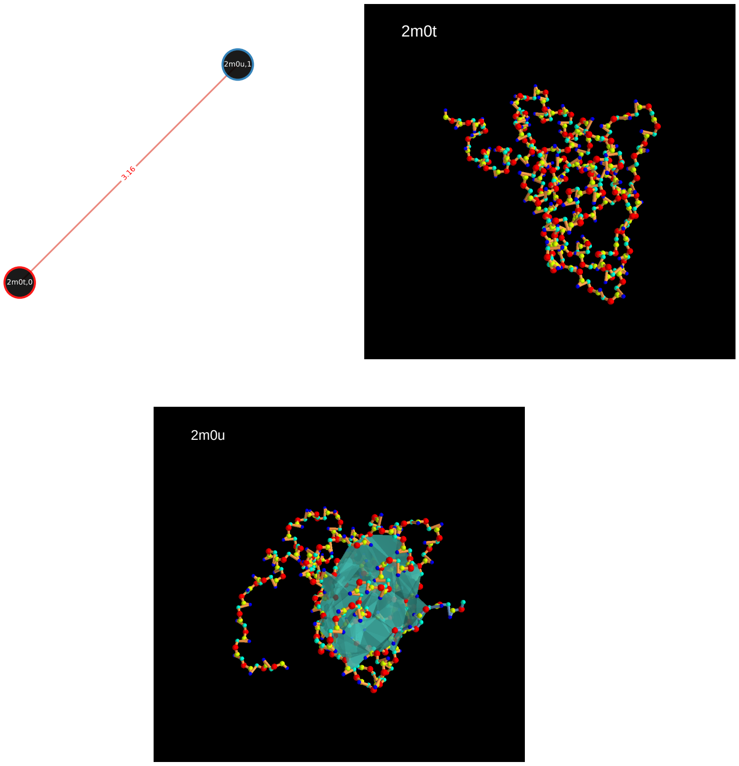

Figure 17

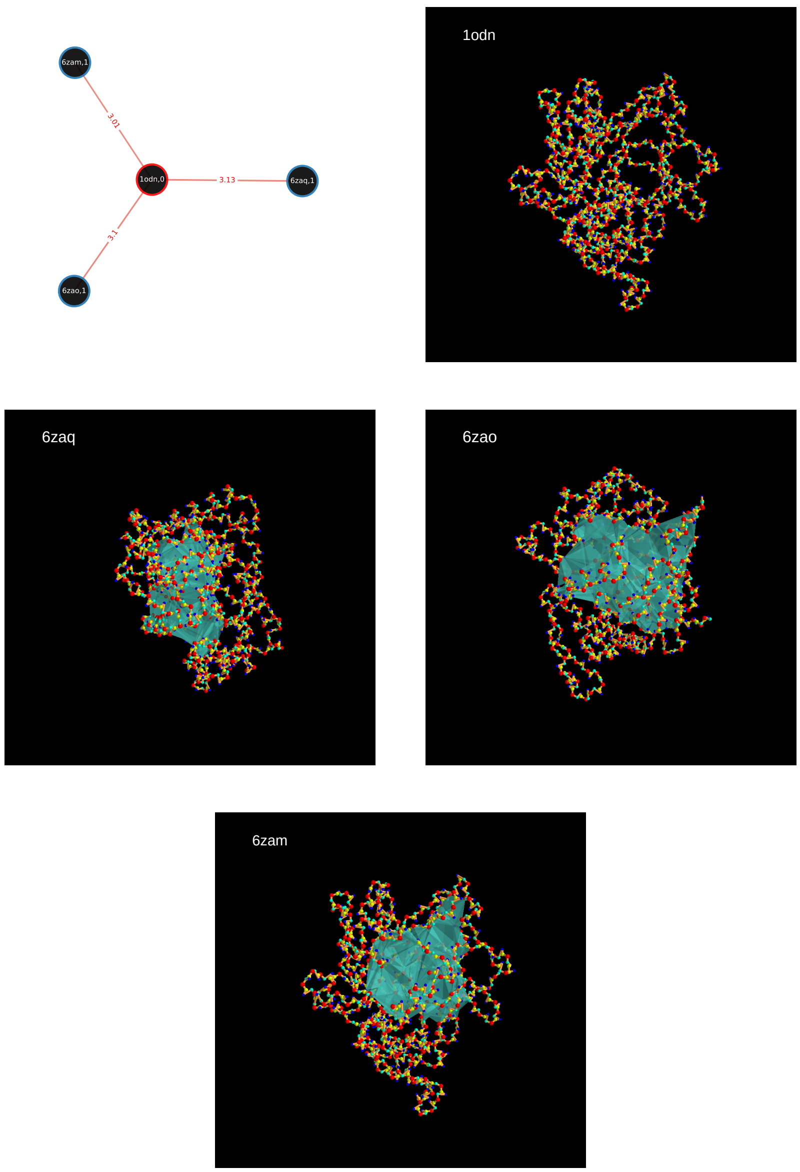

Figure 18

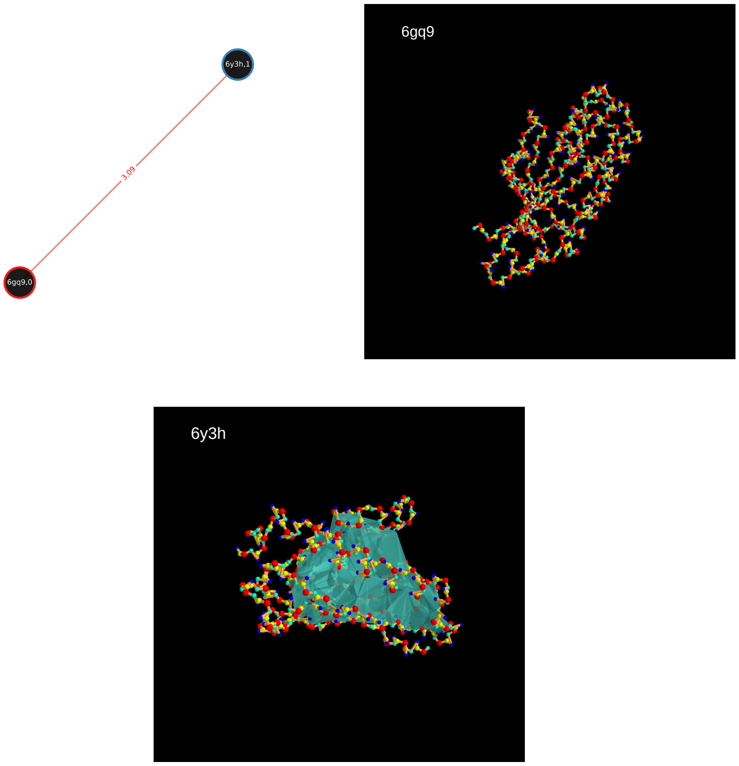

Figure 19

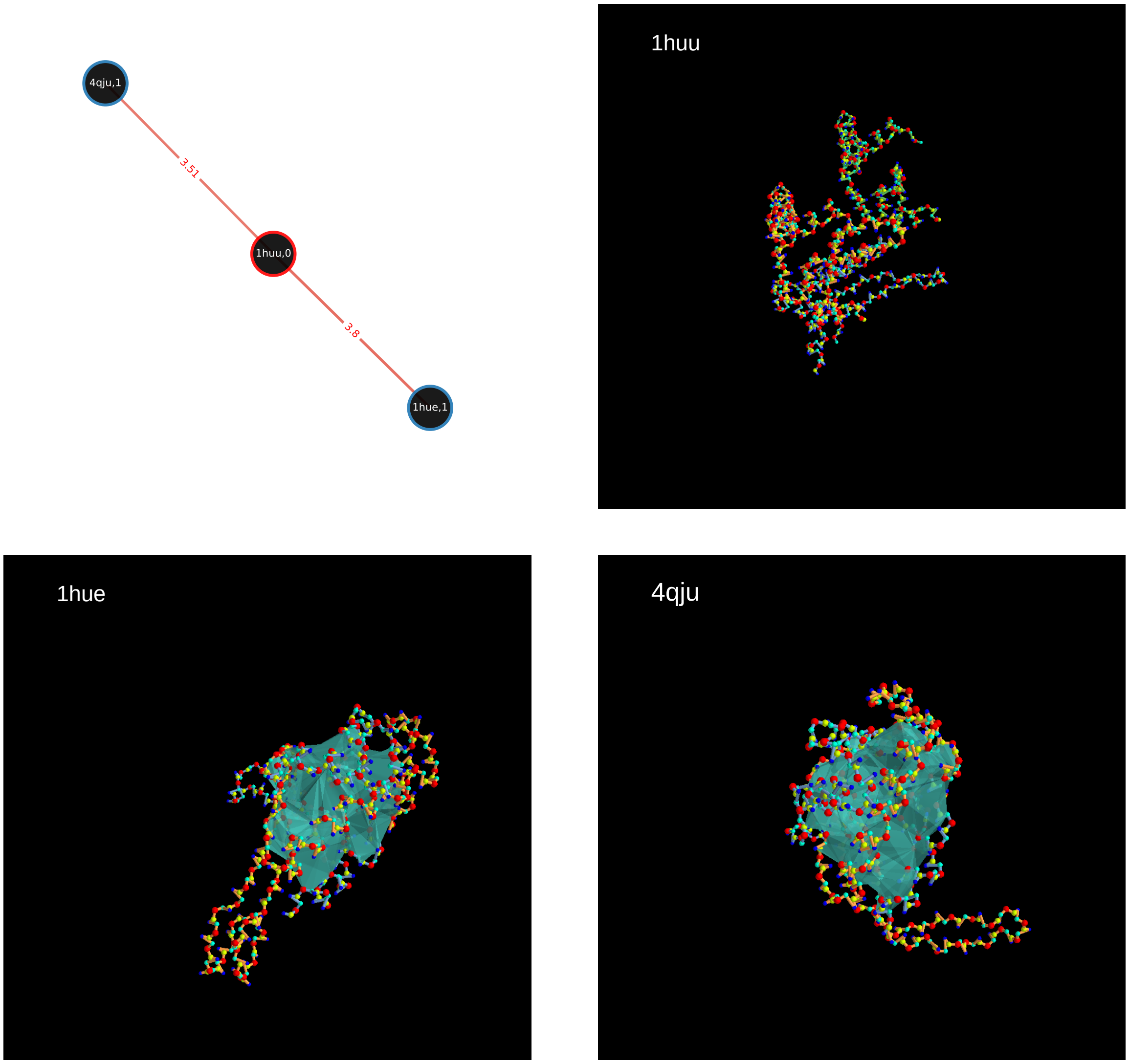

Figure 20

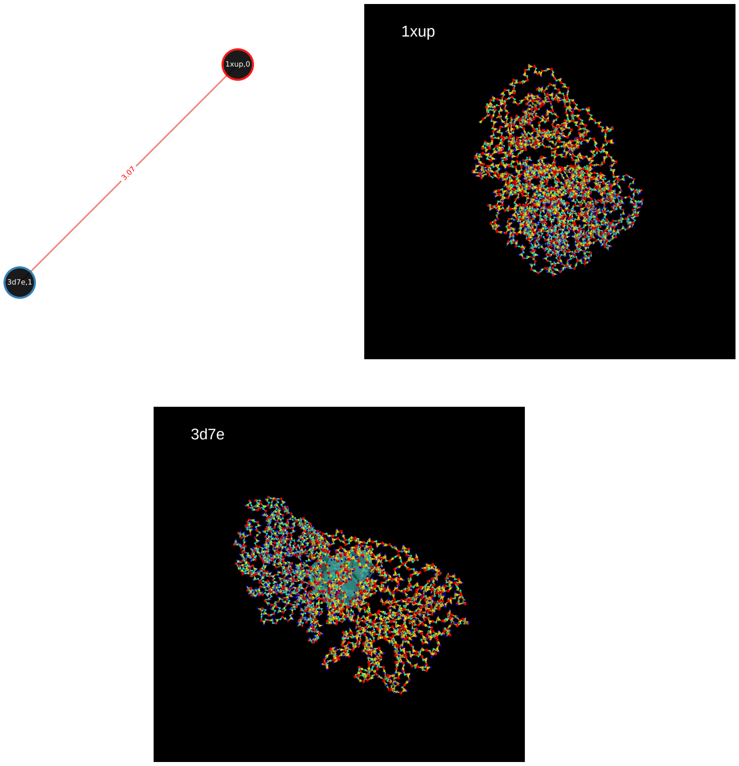

Figure 21

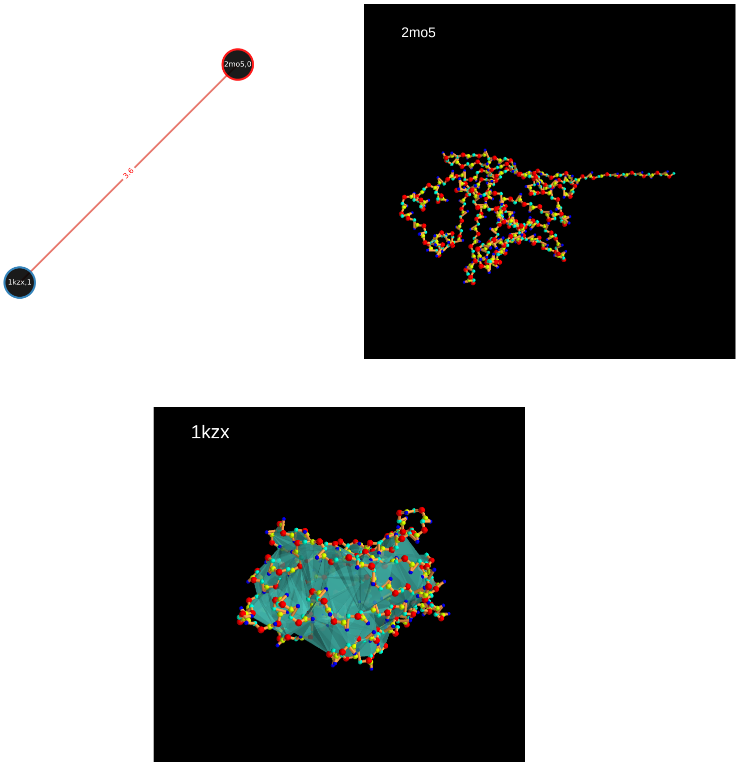

Figure 22

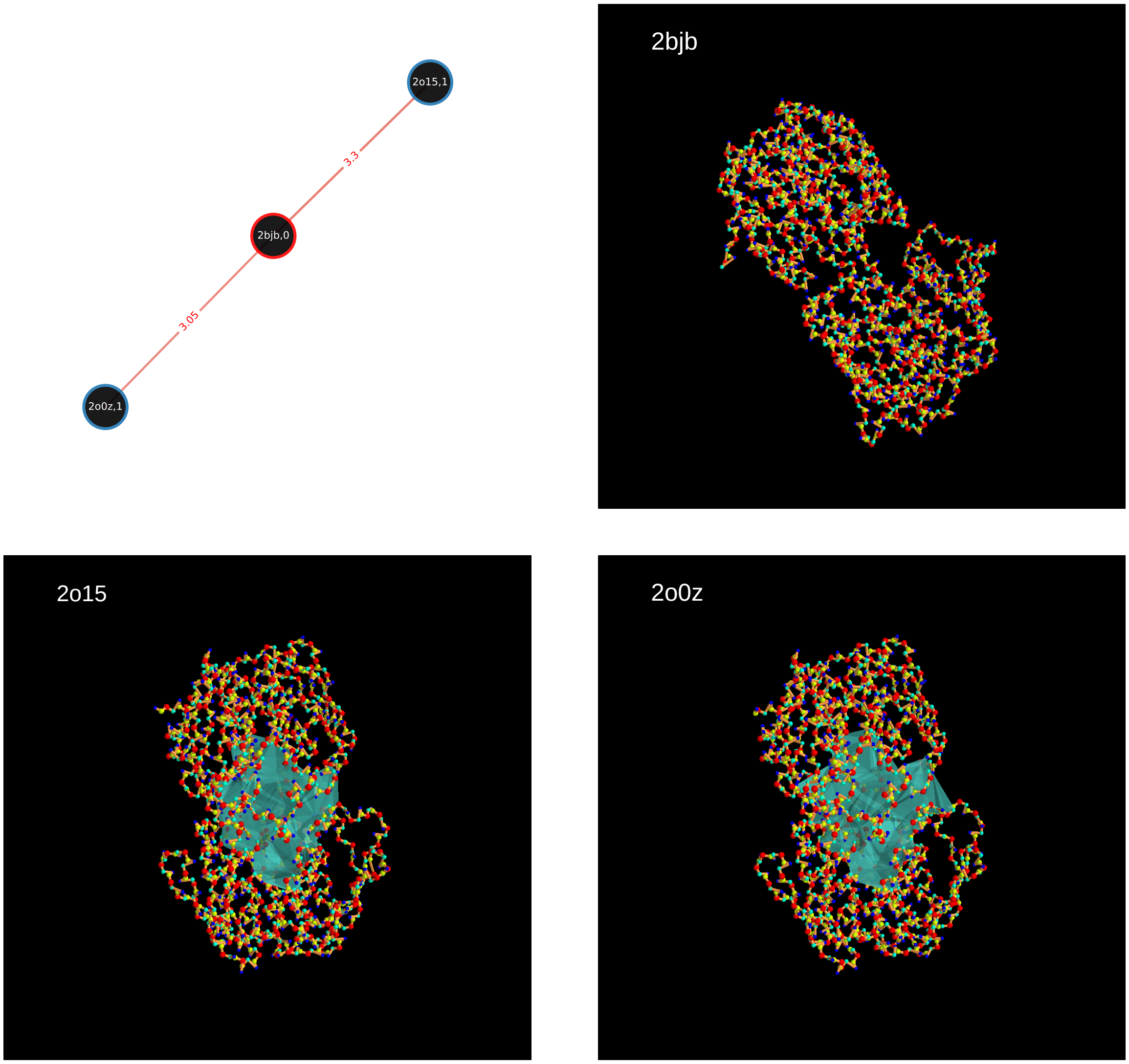

Figure 23

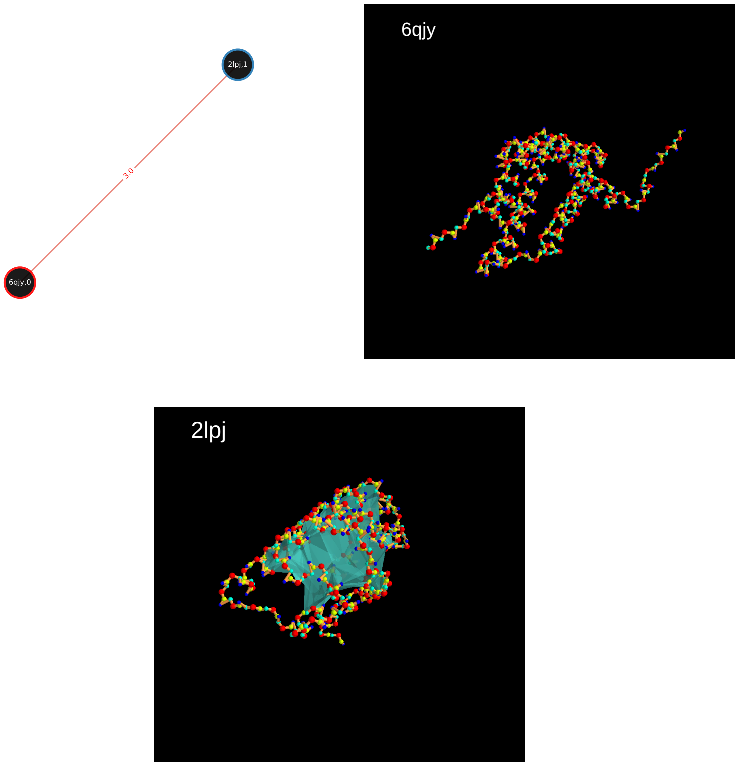

Figure 24

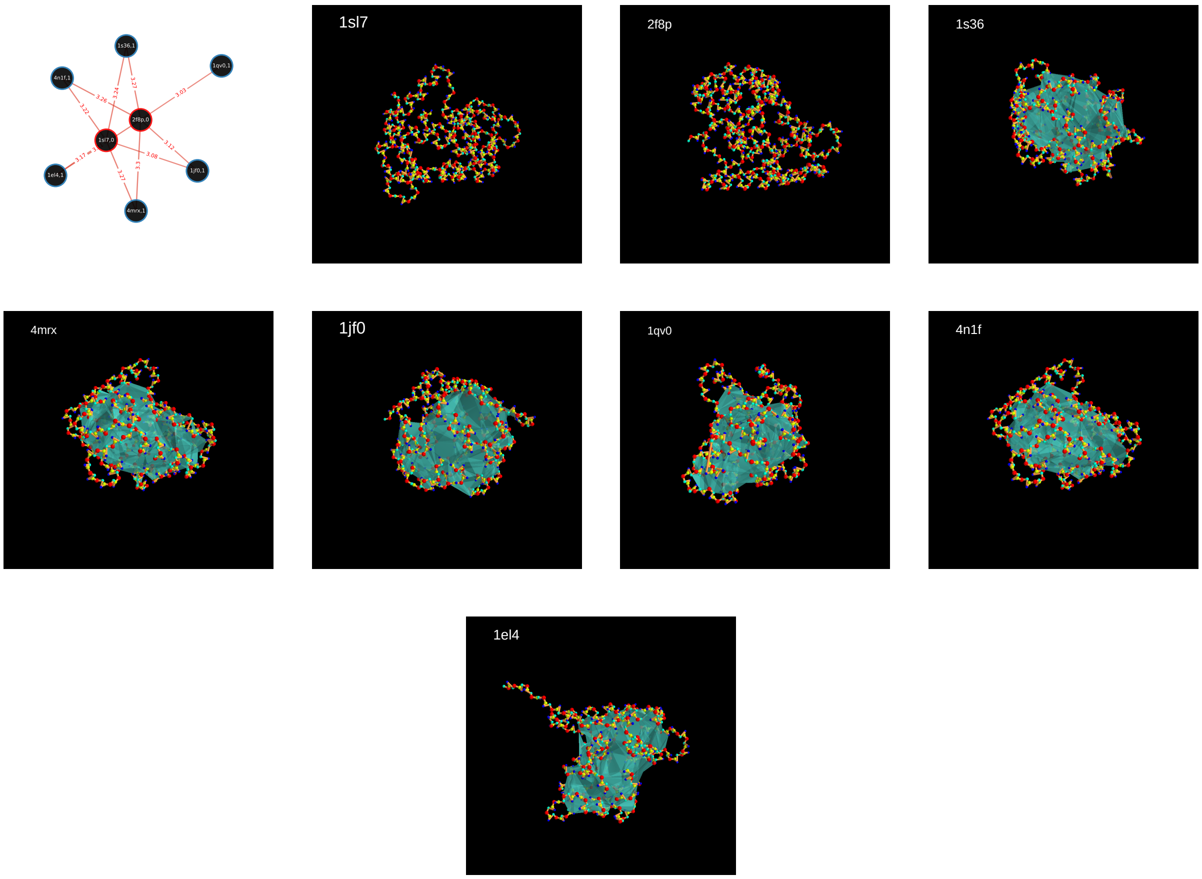

Figure 25
